# Impact of Child Subsidies on Child Health, Well-Being, and Investment in Child Human Capital: Evidence from Russian Longitudinal Monitoring Survey 2010–2017

**DOI:** 10.1007/s10680-023-09653-8

**Published:** 2023-04-20

**Authors:** Alex Proshin

**Affiliations:** 1 Canadian Center for Health Economics, University of Toronto, 155 College St 4th Floor, Toronto, ON M5T 3M6 Canada; 2 Hospinnomics, Paris School of Economics - AP-HP, Paris, France

**Keywords:** Child subsidy, Child outcomes, Household spending, Maternity capital, Regression discontinuity, C52, I11, I31, I38, J18

## Abstract

This study evaluates the impact of introducing the Maternity Capital (MC) program—a child subsidy of 250,000 Rub (7,150 euros or 10,000 USD, in 2007)—provided to mothers giving birth to/adopting a second or subsequent child since January 2007. Eligible Russian families could use this subsidy to improve family housing conditions, fund child’s education/childcare, or invest in the mother’s retirement fund. This study evaluates the impact of MC eligibility on various child health and developmental outcomes, household consumption patterns, and housing quality. Using data from the representative Russian Longitudinal Monitoring Survey 2010–2017, I tested regression discontinuity models and found that MC eligibility may have led to a small improvement in child health status, which could be explained by improved housing conditions, particularly in rural areas. However, children living in MC-eligible families were also more likely to report reduced socialisation. Heterogeneity analysis by child gender, household poverty status, and urban/rural residence suggests that MC incentives may have had a differential impact on some analysed outcomes. Results are robust to different polynomial and nonparametric RDD specifications.

## Introduction and Literature Review

Faced with an aging population and the global trend of decreasing fertility, many governments increasingly regard pro-natalist policies and their impact on households as an issue of primary concern. At the beginning of 2007, the Russian government implemented a reform whereby second and subsequent childbirths/child adoptions were incentivised by a government-sponsored subsidy of around 10,000 USD. This subsidy, called the Maternity Capital (MC), could be spent on three eligible purposes: improving housing conditions, providing education/childcare to children, or investing in the mother’s state pension fund account. This study aims to evaluate how this pro-natalist measure affected multiple measures of child health and well-being, parents’ willingness to invest in child human capital, as well as major household consumption patterns and household financial behaviour.

The results presented in this study indicate that, at the aggregate level, MC eligibility did not significantly affect the patterns of time spent by children, their well-being outcomes, or household diets. Model estimates suggest that children living in MC-eligible households may have benefited from a reduction in the number of chronic health conditions. This effect seems to be more pronounced in rural areas, where MC-eligible families reported a significant improvement in access to essential amenities and public utilities (e.g. central heating and sewerage). On the other hand, results also indicate that children in MC-eligible households could face socialisation issues that manifested themselves, for example, by a more than 30% decrease in the likelihood that the child will be reported by their parents to see their friends at least three times a week. This effect appears to be driven primarily by wealthier households and seems to affect boys more strongly than girls.

Regarding household-level outcomes, MC-eligible families were estimated to make higher bank loan monthly payments, particularly wealthier households and households living in urban areas. Their consumption and spending profiles (e.g. higher spending on essential items and utilities, reduced expenditures on clothing) are consistent with using the MC subsidy to improve housing conditions.

The contribution of our study to the existing economics literature is threefold. First, it studies a broad set of child well-being outcomes, some of which, to the best of my knowledge, have never been investigated in the economics literature. In particular, no earlier study systematically looked at the impact of child subsidies on time spending patterns by children. Some of these metrics, such as time spent on extracurricular study and arts, serve as likely proxies of parental willingness to invest in the child’s human capital. Second, this paper is one of the very few studies that address the link between child subsidies and household diets, household financial behaviour, and housing conditions, which are believed to have long-lasting effects on children’s health and development. Lastly, our study fills a relative lack of research on the impact of pro-natalist reforms in middle-income transitioning countries, whose distinct features (e.g. weaker social security, a higher degree of uncertainty with respect to future income) may create incentives different from those commonly observed in developed countries.

Previous studies on the MC concentrated on its impact on female fertility. For example, Slonimczyk and Yurko (2014) tested a dynamic stochastic model of fertility, wherein women simultaneously make decisions as to whether to have a child and with regard to their participation in the labour market. The authors concluded that the MC subsidy more strongly affected the second childbirths as opposed to the first childbirths, as well as married women. However, the authors did not find sufficient evidence for a differential impact of MC on fertility based on women’s current employment status or urban/rural residence.

As far as analysed outcomes as concerned, few studies concentrated specifically on the effects of child subsidies on child well-being. In 2004, the Australian government introduced a universal $3000 lump-sum all-purpose cash transfer to Australian families who had childbirth or adopted a child, called a Baby Bonus. Similar to the MC, this subsidy was not means-tested. This reform was studied by Gaitz and Schurer (2017) and Deutscher and Breunig (2018), who, using similar difference-in-difference designs and data sources, concluded the reform had little effect on various educational, physiological, and physical health outcomes of pre-school children.

As for child health indicators, empirical research tends to support the idea that material investments made in early childhood could generate benefits in terms of health outcomes (Baughman & Duchovny, 2016; Case et al., 2002; Currie, 2009). Similar effects are observed for child cognitive development and educational attainment, as evidenced, for example, by Dahl and Lochner (2012), who find that the introduction of tax benefits in the USA between 1993 and 1997 helped raise scores in reading and math tests taken by children living in affected households.

The remaining parts of this paper are organised as follows: Section 2 describes the Maternity Capital eligibility conditions and describes the Russian institutional context. Section 3 describes RLMS data used in this study, and Sect. 4 explains the empirical design and provides the main estimation results. Heterogeneity analysis is conducted in Sect. 5. Section 6 discusses the contribution, limitations, and external validity of this study and concludes.

## Maternity Capital Institutional Context

After the collapse of the Soviet Union, Russia entered an acute economic and social crisis that profoundly changed the demographic dynamics of the country. Lack of access to essential goods such as food, disrupted supply chains, mass unemployment, and underfunded healthcare led to a simultaneous increase in mortality and a drastic fall in fertility. To illustrate, in one decade, the total fertility rate in Russia fell from 2.06 in 1989 to an estimated 1.16 in 1999.

In the 2000s, Russia’s economic situation revived, which was accompanied by steady growth in disposable income and an improved living standard. In an attempt to boost falling fertility rates, in January 2007 the Russian government started funding the subsidy called Maternity Capital targeted at mothers with multiple children.

According to eligibility conditions, MC can be claimed by any individual holding Russian citizenship and falling into one of the two categories:

1. women who gave birth to or adopted a second or consecutive child after 1 January 2007
2. men who from 1 January 2007 onward is recognised as the sole adopter of a second, third, or subsequent children, and who did not previously use his right to the Maternity Capital

During the period analysed in this study, MC funds were allowed to be used towards improving housing conditions (through co-funding purchase of the real estate, paying part of the mortgage or repairing/improving current housing), paying for education/childcare expenses of (any) child in the family aged 3–25 years old, and investing in mother’s pension fund.

The MC subsidy certificate has to be granted to an adult family member. For simplicity, I will further refer to potential MC recipients as mothers, leaving the male population outside the scope of the analysis. In general, there is a 3-year delay before an MC subsidy can be issued. However, it does not apply to cases where funds are used to make a downpayment, or fund part of a mortgage, in which case the MC subsidy can be used almost immediately after the eligibility criteria are met. MC funds may be split between multiple purposes and beneficiaries.

In a broader context, the size of MC—250 000 Rubles in 2007 (equivalent to around $9800 US in 2007)—is a very significant sum of money for most Russian families. To put it into perspective, in 2011, the average salary in Russia was estimated to be 23369 Rubles (725 USD in 2011 prices), while the maximum monthly unemployment benefit was set at 4900 Rubles (163 USD in 2011 prices, less than 1/4 of the average salary amount). Thus, MC certificates are approximately worth one year’s average salary amount *(ROSSTAT, 2020)*.

In general, Russian political institutions are considered to be lacking in accountability and the rule of law. Partially as a result of this, a relatively small fraction of the Russian population claims to have trust in public institutions, be they federal or local, executive, legislative or judicial, with one major exception from this rule being the Russian president (Levada Center, 2014). The lack of trust in Russian state institutions plausibly explains the unwillingness of MC-eligible Russian women to invest the subsidy in the pension fund.

## Data and Descriptive Statistics

The primary data source for this study is the Russian Longitudinal Monitoring Survey (RLMS). It is a panel survey conducted every year since 1994 for a representative sample of both Russian individuals and households. RLMS features three core modules: adults, children, and households. The resulting datasets contain an extremely rich set of variables that cover virtually all areas of respondents’ lives (e.g. employment, income, health, education, attitudes towards social issues, and family relationships). RLMS interviews occur every year at the respondent’s home, normally from October to January the following year, as long as participating families continue to live at the same address and are willing to participate in the study.

**Table 1:** Descriptive statistics for the 2nd children (answers from RLMS 2010-2017 respondents with 2nd children born between 2004 and 2010 and aged 6–8 years, in households with two children), by MC eligibility

Variable	All sample				MC eligible		MC ineligible	
	Mean	SD	Min	Max	Mean	SD	Mean	SD
*Child characteristics*								
Age	6.979	0.812	6	8	6.96	0.805	6.998	0.819
Sex (male)	0.481		0	1	0.539***		0.424***	
Year	2013.618	1.889	2010	2018	2015.12***	1.176	2012.118***	1.115
*Health outcomes*								
In good/excellent health	0.772		0	1	0.787		0.757	
Health score (1-highest, 5-lowest)	2.204	0.512	1	5	2.201	0.514	2.207	0.51
Health problem in last 30 days	0.345		0	1	0.373*		0.317*	
Num. of chronic conditions	0.299	0.635	0	5	0.304	0.676	0.294	0.591
*Time spending (in mins per week)*								
Extracurricular study	14.462	54.406	0	720	13.725	55.085	15.159	53.819
Extracurricular arts	120.048	195.486	0	1920	118.366	188.828	121.64	201.826
Extracurricular sports	219.844	336.195	0	2200	201.913	305.726	236.825	362.289
Watching TV/on Internet	121.78	85.596	0	960	120.098	89.797	123.373	81.505
*Child well-being*								
Used a personal computer	0.667		0	1	0.628**		0.704**	
Vacation with parent in 1 yr	0.672		0	1	0.669		0.676	
Num. of excursions/exhibition visits in 1yr	4.382	5.556	0	35	4.299	5.17	4.462	5.912
Sees friends $>2$ times per week	0.623		0	1	0.585**		0.662**	
Received childcare in past 7 days	0.329		0	1	0.333		0.324	
Received childcare from a relative in past 7 days	0.178		0	1	0.187		0.168	
*Mother’s characteristics*								
Age	36.655	5.119	25	53	37.015**	5.619	36.296**	4.544
Urban	0.585		0	1	0.583		0.588	
Single parent	0.145		0	1	0.14		0.151	
In good/excellent health	0.482		0	1	0.509		0.456	
Higher education diploma	0.3		0	1	0.34***		0.26***	
Non-Russian ethnicity	0.122		0	1	0.126		0.118	
Poverty	0.415		0	1	0.436		0.395	
Alcohol cons. $>1$ time per week	0.036		0	1	0.031		0.042	
Subjective life satisfaction (1-excellent, 5-bad)	3.39	1.08	1	5	3.377	1.112	3.403	1.048
Number of observations	1047				515		532	

Stars denote *p*-values for t-tests on equality of means (non-binary variables)/Chi-square tests on equality of proportions (for binary variables): ***− 1 % sign., **− 5% sign., *− 10% sign

**Table 2:** Descriptive statistics for household variables, for households with a second child (answers from RLMS 2010-2017 respondents with second children born between 2004 and 2010 and aged 6–8 years, in households with two children), by MC eligibility

Variable	All sample				MC eligible		MC ineligible	
	Mean	SD	Min	Max	Mean	SD	Mean	SD
*Household consumption (per week)*								
Vegetables/legumes	2.285	4.124	0	30	2.329	4.259	2.243	3.991
Fruit (fresh and canned, in kg)	3.65	3.903	0	26	3.435*	3.593	3.861*	4.179
Meat and poultry (in kg)	3.228	3.497	0	30	3.313	3.538	3.143	3.457
Dairy (in kg)	5.835	4.711	0	33.3	5.927	4.485	5.746	4.923
Vodka and liquors (in litres)	0.169	0.593	0	8	0.148	0.534	0.19	0.646
Refined sugar (in kg)	1.388	2.265	0	20	1.51*	2.378	1.265*	2.141
Candy and high-sugar treats (in kg)	1.679	1.513	0	12	1.692	1.513	1.666	1.513
Starches (in kg)	10.231	9.217	0	69	10.394	9.105	10.068	9.335
*Household spending* *(in Rub per month, 2011 prices):*								
Essential food items	9.555	6.225	0	47.344	9.492	6.112	9.617	6.341
Clothes	4.389	4.266	0	38.185	4.083**	3.867	4.694**	4.614
Purchase of durable goods	0.371		0	1	0.329***		0.412***	
Utilities	3.207	3.26	0	32.175	3.241	3.497	3.173	3.008
Essential services	2.277	3.645	0	58.767	2.293	4.181	2.261	3.02
All basic expenditure	19.427	10.664	0.78	73.632	19.108	10.634	19.744	10.695
Non-essential expenditures	5.121	14.715	0	224.183	4.242*	8.391	5.998*	19.006
*Lending, borrowing, and saving*								
Household income in last 30 days (in 2011 prices)	42.462	37.75	0.5	672.303	42.037	27.975	42.887	45.482
Household savings in last 30 days (in 2011 prices)	1.374	7.718	0	152.742	0.966*	6.066	1.78*	9.059
Loan payments in last 30 days (in 2011 prices)	4.187	7.785	0	60	4.668**	8.805	3.706**	6.586
Savings will last several months	0.203		0	1	0.184		0.221	
Consumer loan in last 12 months	0.177		0	2	0.126***		0.228***	
Borrowed from a private individual in last 12 months	0.085		0	1	0.099		0.071	
Loan to a private individual in last 12 months	0.09		0	1	0.071**		0.109**	
*Spending share in household income, last 30 days*								
Essential food items	0.308	0.287	0	3.635	0.299	0.289	0.317	0.284
Clothes	0.137	0.163	0	2.258	0.125**	0.126	0.149**	0.193
Utilities	0.095	0.149	0	3.774	0.089	0.093	0.101	0.189
Essential services	0.063	0.082	0	0.945	0.06	0.071	0.065	0.091
All basic expenditure	0.602	0.475	0.04	8.312	0.573**	0.405	0.631**	0.535
Non-essential expenditure	0.147	0.528	0	11.21	0.117*	0.266	0.176*	0.697
Savings	0.026	0.129	0	2.4	0.02	0.135	0.031	0.124
Loan payments	0.121	0.254	0	4	0.118	0.204	0.124	0.296
*Housing characteristics*								
Household owns home	0.889		0	1	0.899		0.879	
Hot water supply	0.571		0	1	0.564		0.577	
Central sewerage	0.621		0	1	0.602		0.641	
Central heating	0.586		0	1	0.568		0.605	
Number of observations	1047				515		532	

Stars denote *p*-values for t-tests on equality of means (non-binary variables)/chi-square tests on equality of proportions (for binary variables): ***− 1 % sign., **− 5% sign., *− 10% sign

In total, 16 child outcomes related to health and development, material conditions, and the degree of parental involvement in child upbringing were analysed to evaluate the impact of MC on child well-being. In addition, this analysis is complemented with a household-level study of the MC impact on dietary habits, spending patterns, financial behaviour, and housing conditions, which are likely to affect all family members directly or through spillover effects.

The main analytical sample is restricted to second children born between 2004 and 2009 (i.e. three years before and after the introduction of Maternity capital in 2007) and aged from 6 to 8 years old, whose parents were surveyed in RLMS waves from 2010 to 2017 and living in households with two children. The observations were collected from RLMS waves from 2010 to 2017, with the median year of 2013. However, since the full analytical sample has a 3-year window around the cut-off date of 1 January 2007, the measurements for MC-eligible and MC-ineligible families are separated by, on average, three years (median survey years are 2012 for MC-ineligible families, and 2015 for MC-ineligible ones). A given child and the corresponding household can be observed a maximum of 3 times, when the child is 6, 7, and 8 years old. However, due to sample attrition and restricting the analytical sample only to the cohort of children aged 6–8 and born in 2004–2009, the average number of times a given child/household appears in the sample is 2.26 (25.3%- 1 time, 23.3% - 2 times, 51.4% -3 times). The annualised attrition rate (i.e. the average rate at which children aged 6-7 do not appear in the next RLMS wave, for RLMS waves 2010-2016) in the analysed sample is 19.2%. Additional tables on the time distribution of observations, as well as other descriptive statistics, are provided in Tables 8, 9, and 10 of Appendix 2.

Descriptive statistics on the sample of second children and both MC-eligible and MC-ineligible subgroups are provided in Tables 1 and 2. In total, the analytical sample contains 1047 observations (i.e. a child observed in a given RLMS wave, who could be observed in up to three waves in total), 515 of whom (49.2%) represent children who were born after the MC program was introduced in January 2007. Children born before and after 1 January 2007 had broadly similar characteristics in many health and developmental outcomes. In particular, participating respondents considered that their children were in good/excellent health in around 77% of cases, with an average of 0.3 chronic medical conditions reported per child. However, MC-eligible families also report a slightly higher percentage of boys than girls. After school classes, many children were also involved in extracurricular educational activities, such as private lessons, art classes and extracurricular sports/physical activities (14, 120 and 220 minutes per week on average, respectively).

As for mothers, their average age in the analytical sample is around 36.5 years, with 14.5% of observations living in households with only one parent. On average, MC-eligible and MC-ineligible mothers also shared similar characteristics regarding their ethnicity, urban/rural residency, and self-reported alcohol consumption.

At the same time, there is suggestive evidence that the demographics of mothers who responded to MC stimuli may not completely mirror cohort averages. In particular, MC-eligible families reported, on average, a slightly higher age (37 vs. 36.3 years old) and a higher rate of graduating from a higher education institution (34% vs. 26% in MC-eligible and MC-ineligible families, respectively). This likely results from the fact that higher education graduation rates have been increasing in Russia in the 2010s and the fact that the parents of the 2007–2009 birth cohort, on average, are observed later in the analytical 2010–2017 period.

As for household spending patterns, MC-eligible and MC-ineligible families reported on similar average levels of essential expenditures. However, MC-eligible families were less likely to report the purchase of a durable good (32.9% vs. 41.2%) and had lower spending on clothes (i.e. 4.1K Rub/135 USD vs. 4.7K Rub/155 USD, in 2011 prices) and on non-essential items (i.e. 4.2K Rub/140 USD vs. 6.0K Rub/200 USD, in 2011 prices). In the meantime, MC-eligible households had a heavier monthly bank loan burden (i.e. 4.7K Rub/155 USD vs. 3.7K Rub/125 USD, in 2011 prices) and lower rates of taking out a consumer loan or lending to private individuals in the last one year.

Overall, MC-eligible and MC-ineligible families reported broadly similar diets, with minor differences in fruit and refined sugar consumption. Finally, in terms of reported housing conditions, the vast majority (89%) of Russian families own their housing.

More detailed definitions of child outcomes, spending and consumption categories, and other variables used in this study are provided in Appendix 1.

## Empirical Strategy and Main Results

The empirical strategy of this study MC relies on the fact that MC eligibility is dependent on the precise cut-off date of 1 January 2007. After this date, any family giving birth to second or subsequent children was automatically given MC rights. To ensure that results are comparable among analysed households, I concentrate solely on families with two children, who constitute by far the most common case among MC-eligible families.

The prospect of the MC reform was first announced during the presidential address to the members of the Russian parliament in May 2006. However, no precise timeline for MC implementation was given, which makes it highly unlikely that the fertility decisions of Russians of their reproductive age were affected at this point. Aggregate official figures on childbirth by month published in Demoscope (2019) also suggest that the MC effect on fertility started to take shape only in August 2007.

The focus of the strategy consists in comparing the outcomes of interest in the immediate neighbourhood of the intervention cut-off date, where, theoretically, any observed differences in outcome variables must be solely attributable to the intervention due to near-complete treatment randomisation. To this end, I select children born within 12-, 24-, and 36-month windows around the intervention cut-off date.

It appears possible that mothers’ fertility responses to the MC were not identical across observed socio-demographic characteristics, especially several months/years after the introduction of the MC. Slonimczyk and Yurko (2014) point to the possibility that, for example, married and poorer/less-educated women reacted more strongly to MC incentives but the differences they found were very modest and insignificant. Appendix 2 provides descriptive statistics by the year of birth, and Appendixes 4 and 5 also test RDD models for mother and household covariates around the cut-offs of January and August 2007, respectively. The results are suggestive of the possibility of self-selection into MC based on, for example, the higher education status, which in all likelihood could only affect births that occurred at least 8–9 months after the introduction of the MC.

To estimate the effect of MC on child outcomes, I test regression discontinuity models of the functional form:

$$\begin{aligned} \textrm{Outcome}_{ip}= & {} \alpha + \sum _{k=1}^{3}(\gamma _{k}\textrm{trend}_{t}^{k} + \tau _{k}\textrm{post}_{t} \cdot \textrm{trend}_{t}^{k}) + \beta \textrm{post}_{t} + \lambda ' X_{ip} + \theta ' Z_{ip}+ \nu _p+ \epsilon _{ip}, \end{aligned}$$

where for a child *i* born in year-month *t* and observed in RLMS survey wave *p*, $Outcome_{ip}$ is the analysed outcome of interest, $trend_{t}$ is a linear trend for the month of birth relative to January 2007 and $trend_{t}^{k}$ is its exponent of the $k^{th}$ degree; $post_t$ is an indicator variable for births occurring on and after the 1 January 2007, $X_{ip}$ is a vector of child-specific controls including child’s age (in months), sex and urban (i.e. city or regional centre)^1^; $Z_{ip}$ is a vector of mother and household-specific controls, including mother’s age (in years) and a set of dummies indicating higher education, excellent/good health status, non-Russian ethnicity, self-declared poverty status, frequent alcohol consumption and the indicator that she raises a child as a single parent; $\nu _p$ are RLMS wave dummies; $\epsilon _{ip}$ is a random error term. To account for potential intertemporal and intraregional correlation of error terms between observations, in all tested models error terms are clustered at the regional level. Apart from implementing standard tests for coefficient statistical significance, I also report coefficients that remain significant after the Bonferroni correction for multiple outcome comparisons.

The main coefficient of interest $\beta$ stands for the impact of MC eligibility on analysed outcome variables. A considerable advantage of regression discontinuity specifications lies in the fact that this design makes use of theoretically near-complete randomisation at the immediate neighbourhood of the intervention cut-off threshold, thus providing unbiased estimates of local average treatment effects (LATE). However, it is worth noting that the validity of RDD models critically depends on the correctness of functional form assumptions. This consideration leads me to test for each analysed outcome different sets of models that vary in terms of the functional form of the trend (ranging from linear and to 3rd degree of polynomial), the birth timeline window around the cut-off period of January 2007 (models with 36-, 24-, 12-month windows), as well as the inclusion of other covariates. In addition, to allow for more functional flexibility, nonparametric local polynomial regressions (Lowess) are tested as robustness checks and their results are provided in Appendix 3.1.

### Child Health Outcomes

First, I evaluate the MC impact on a range of reported child health outcomes. There are several channels through which MC may have affected child health. For example, better housing conditions are known to be associated with lower rates of infections (Ali et al., 2018), and better general physical and mental health (Pevalin et al., 2017; Rolfe et al., 2020).

**Table 3:** Regression discontinuity estimates of impact of MC on 2nd child health outcomes (12, 24 and 36 months’ window at 1 January 2007 cut-off birth date)

Variable	RDD 12m	RDD 12m	RDD 12m	RDD 24m	RDD 24m	RDD 36m	RDD 36m	Relative effect (%)
	(1)	(2)	(3)	(4)	(5)	(6)	(7)	
*Health outcomes*								
In good/excellent health	$-$ 0.022	$-$ 0.023	0.057	0.057	0.136	$-$ 0.027	0.071	0.6
	(0.091)	(0.092)	(0.09)	(0.097)	(0.094)	(0.108)	(0.106)	
Health score (1$-$ excellent, 5$-$ bad)	$-$ 0.001	0	$-$ 0.089	$-$ 0.106	$-$ 0.19	0.022	$-$ 0.083	$-$ 1.3
	(0.099)	(0.099)	(0.096)	(0.109)	(0.107)*	(0.12)	(0.119)	
Num. of chronic conditions	$-$ 0.124	$-$ 0.116	$-$ 0.251	$-$ 0.241	$-$ 0.346	$-$ 0.212	$-$ 0.336	$-$ 41.5
	(0.135)	(0.135)	(0.148)*	(0.146)*	(0.147)**	(0.168)	(0.167)**	
Health problem in last 30d	0.072	0.066	0.051	0.005	$-$ 0.035	0.112	0.084	19.3
	(0.119)	(0.118)	(0.117)	(0.128)	(0.128)	(0.144)	(0.143)	
*Time spent on (in mins per week)*								
Extracurricular study	10.011	6.416	8.347	13.689	16.148	11.412	12.938	74.7
	(10.989)	(10.552)	(11.416)	(10.859)	(10.824)	(11.43)	(11.767)	
Extracurricular arts	44.725	44.192	49.803	19.565	59.873	47.605	85.286	33.6
	(44.905)	(42.553)	(44.471)	(44.516)	(45.371)	(48.837)	(48.431)*	
Extracurricular sports	98.888	99.031	80.147	$-$ 8.994	$-$ 8.362	36.249	47.634	34
	(100.029)	(101.277)	(97.677)	(101.366)	(103.439)	(111.056)	(110.659)	
Total productive time	148.677	146.111	137.829	19.789	65.173	88.071	140.138	34.20
	(113.917)	(112.743)	(110.684)	(114.536)	(120.548)	(124.906)	(128.299)	
Watching TV/on Internet	16.7	19.395	5.544	21.714	17.472	22.282	16.404	11.9
	(20.449)	(20.281)	(21.101)	(21.546)	(21.357)	(23.29)	(23.4)	
*Well-being outcomes*								
Used a personal computer	0.111	0.118	0.141	0.173	0.195	0.238	0.194	11.9
	(0.147)	(0.146)	(0.14)	(0.149)	(0.14)	(0.163)	(0.155)	
Vacation with parent in 1yr	0.014	0.005	$-$ 0.024	0.174	0.199	0.27	0.256	$-$ 0.2
	(0.123)	(0.121)	(0.121)	(0.131)	(0.123)	(0.147)*	(0.138)*	
Num. of excursions/exhibition visits in 1yr	$-$ 3.854	$-$ 3.957	$-$ 3.759	$-$ 2.683	$-$ 2.409	$-$ 3.027	$-$ 3.354	$-$ 79.5
	(1.691)**	(1.699)**	(1.579)**	(1.845)	(1.695)	(2.092)	(1.946)*	
Sees friends $>2$ times per week	$-$ 0.233	$-$ 0.228	$-$ 0.205	$-$ 0.313	$-$ 0.324	$-$ 0.34	$-$ 0.358	$-$ 33.4
	(0.118)*	(0.118)*	(0.123)*	(0.124)**	(0.124)***	(0.139)**	(0.14)**	
Received childcare in past 7 days	0.063	0.065	0.066	$-$ 0.055	$-$ 0.012	0.026	0.039	19.4
	(0.11)	(0.111)	(0.109)	(0.117)	(0.112)	(0.132)	(0.128)	
Received childcare from a relative in past 7 days	$-$ 0.029	$-$ 0.026	$-$ 0.004	$-$ 0.109	$-$ 0.059	0.002	0.035	$-$ 10.3
	(0.09)	(0.089)	(0.088)	(0.097)	(0.093)	(0.107)	(0.103)	
*Mother outcome*								
Mothers subjective life satisfaction	0.093	0.086	0.358	0.472	0.603	0.507	0.685	5.2
(1$-$ lowest, 5$-$ highest)	(0.263)	(0.26)	(0.243)	(0.276)*	(0.255)**	(0.31)	(0.286)**	
*Age controls*	NO	YES	YES	YES	YES	YES	YES	
*Other controls*	NO	NO	YES	NO	YES	NO	YES	
*Linear trend*	YES	YES	YES	YES	YES	YES	YES	
*Quadratic trend*	NO	NO	NO	YES	YES	YES	YES	
*Cubic trend*	NO	NO	NO	NO	NO	YES	YES	
*RLMS year controls*	YES	YES	YES	YES	YES	YES	YES	
Num. obs.	328	328	328	727	727	1047	1047	

***− 1 % sign., **− 5% sign., *− 10% sign. Coefficient std. errors are given in parentheses under the coefficient. Error terms are clustered at regional level. Relative effect sizes are calculated relative to sample means in MC-ineligible families, based on average effects in models with a 12-month bandwidth

During the RLMS interview, the most straightforward health measure is provided by parents when they are asked to assess the state of health for each of their children on a scale from 1 (excellent) to 5 (bad). Children who received scores 1 and 2 were deemed to have good/excellent health. These measures are subjective and are likely to be affected by a host of factors, such as parental education, social background, and, generally, parents’ perception of what stands for good and bad health. To partially overcome this issue, these variables are complemented with more objective health metrics that include the number of reported child chronic conditions and the occurrence of a health problem in the last 30 days.

**Fig. 1:**
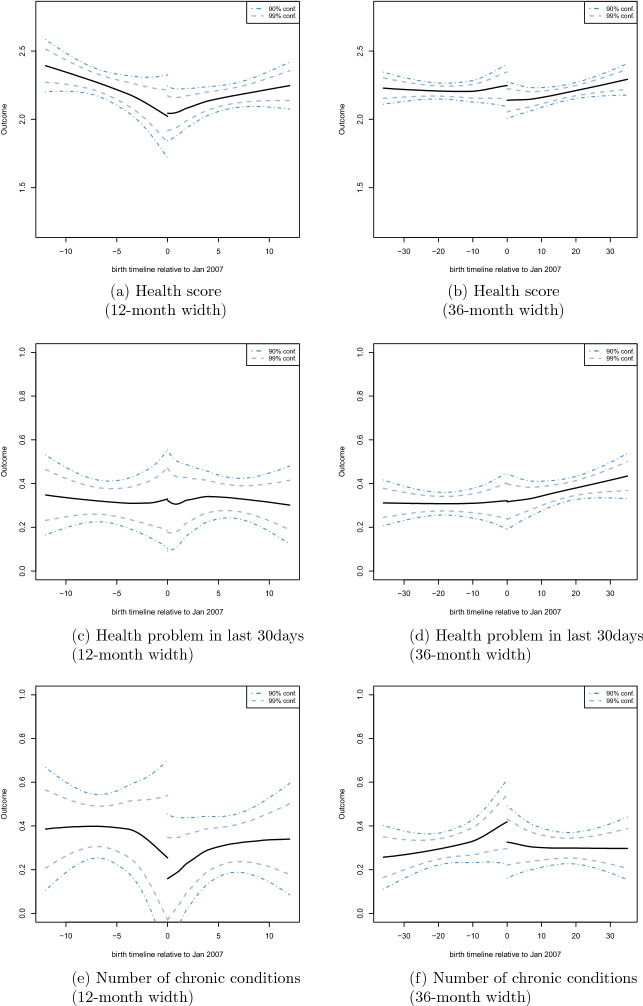
Maternity Capital effect on the 2nd child health outcomes, nonparametric Lowess estimation

Estimation results are presented in Tables 3 and 20 in Appendix 3 and are illustrated by nonparametric Lowess regression fit shown in Figure 1 in Appendix 3. The results suggest that children living in MC-eligible households may have experienced a small overall improvement in general health. Although the coefficients tend to take the sign that is indicative of a positive change, only coefficients for the number of chronic are statistically significant in certain specifications. The MC eligibility is estimated to have reduced the number of reported child chronic conditions by 0.1-0.35. The additional analysis reported in Tables 25, 26, 27 in Appendix 3 suggests that the effect might be driven by rural households who likely invested the MC subsidy into providing central heating in their housing, and from female children. However, due to small sample sizes, heterogeneity analysis estimates tend to be highly volatile. Thus, they should be interpreted as tentative. Additional heterogeneity analysis by the poverty status is provided and by child gender later in this section.

### Child’s Patterns of Time Spending

In this subsection, I evaluate the impact of the MC reform on the formation of human capital and developmentally relevant activities of children. One eligible MC use is funding or co-funding educational and childcare expenses for the child (e.g. private educational classes and programs, private childcare) after they turn three years old. Thus, it may contribute to the child’s human capital formation by providing enriched learning/living environments.

I analyse variables reflecting the amount of time children spend on different out-of-school activities, measured in minutes per week. These activities reflect parental investment in child human capital through providing opportunities for extracurricular activities, most often in the form of private tutoring for a foreign language, other school subjects, extracurricular arts (including evening art school, dance classes) and extracurricular sports activities. The variable total productive time sums all time spent by children on these activities. In addition, I include a variable on the parent-reported amount of time a child spends watching TV/using a computer for non-educational purposes. This outcome serves as a proxy for the time devoted to unproductive leisure activities that generally do not significantly contribute to the formation of the child’s human capital.

**Fig. 2:**
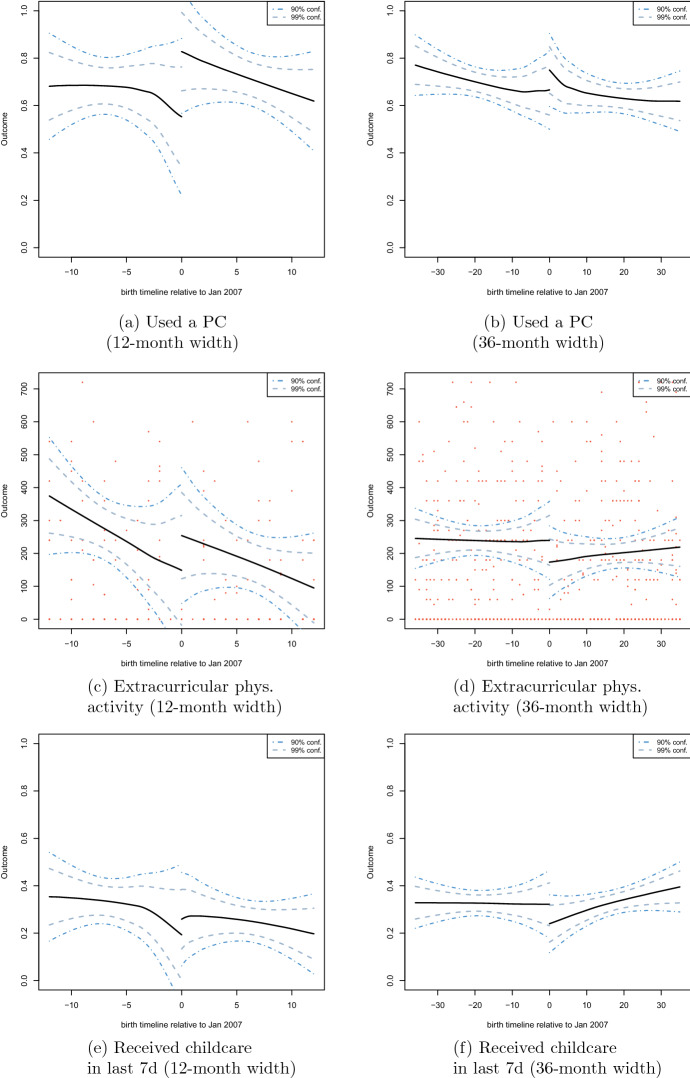
Maternity Capital effect on the 2nd child outcomes, nonparametric Lowess estimation

Estimation results for RDD models with varying sets of covariates and window widths are provided in Table 3 and are visually illustrated by nonparametric Lowess regression fit in Figure 2 in Appendix 3. Overall, outcomes show statistically insignificant results. However, with regard to extracurricular studies and arts, relatively stable coefficient signs and magnitudes across specifications indicate that small shifts may have occurred in these two variables (10–15 and 40–80 min per week increase, respectively).

### Child Well-Being Outcomes

The third set of child outcomes analysed in this section addresses the child’s well-being and parental effort to ensure a sufficient level of child’s material and emotional comfort. To measure factors that are known to influence the level of children’s life satisfaction, I included in the set of studied outcomes: the reported number of attended cultural events/trips (e.g. exhibitions, museums, galleries, cinema theatres) in the past 12 months; an indicator variable for the child seeing their friends regularly at least three times a week; an indicator variable that at least one parent spent the child’s school vacation with the child, and the reported use of a personal computer (for any purposes). In addition, since MC could be spent on private childcare services after the child turns three years old, I included indicator variables for receiving any childcare within the last seven days from any individual who does not belong to the household and a similar indicator for receiving childcare from a family member who does not live in the same household. Finally, due to the presumed significant interdependence between the child’s and the mother’s well-being, I included the reported subjective measure of the mother’s life satisfaction as an outcome variable.

RDD estimates of the MC eligibility effect on the described well-being outcomes are provided in Table 3. Findings suggest that MC may have adversely affected child socialisation by reducing the number of children with frequent contact with friends by 20-30 p.p. The hypothesised channel is that MC allowed certain families to move to other residential locations, which may have impacted the robustness of the child’s social networks. The heterogeneity analysis presented in this Sect. 5 and Appendix 3 suggests that male children and those who lived in an urban environment at the moment of the RLMS interview, as well as wealthier households, were affected more strongly by this effect.

Table 3 also provides evidence that the frequency of attending cultural events may have decreased by around 3–3.5 events per year in MC-eligible households. Heterogeneity analysis in Appendix 3 points to the possibility that this effect may be primarily driven by children living in urban settings and poorer households, and who also tend to be female.

Finally, in some specifications, MC shows a positive effect on mothers’ subjective life satisfaction (around 0.3–0.6 points in models that yield statistically significant estimates). In Table 28 of Appendix 3, this effect appears more accentuated in households living in wealthier families, who, other things equal, may also have had more available options to benefit from the MC program.

### Household-Level Outcomes

The last set of analysed outcomes concentrates on household-level outcomes, including general spending patterns, household diets, household financial behaviour, and housing conditions.

**Table 4:** Regression discontinuity estimates of impact of MC on household characteristics (12, 24 and 36 months’ window at 1 January 2007 cut-off birth date)

Variable	RDD 12m	RDD 12m	RDD 12m	RDD 24m	RDD 24m	RDD 36m	RDD 36m	Relative effects (%)
	(1)	(2)	(3)	(4)	(5)	(6)	(7)	
*Consumption by dietary group*								
Vegetables/legumes (in kg)	$-$ 0.812	$-$ 0.819	$-$ 1.209	$-$ 0.469	$-$ 0.753	$-$ 1.162	$-$ 1.425	$-$ 41.6
	(1.494)	(1.492)	(1.496)	(1.622)	(1.636)	(1.84)	(1.871)	
Fruit (fresh and canned) (in kg)	$-$ 0.71	$-$ 0.721	$-$ 0.618	0.12	0.1	$-$ 0.399	$-$ 0.495	$-$ 18.6
	(1.031)	(1.039)	(1.126)	(0.974)	(1.008)	(1.143)	(1.18)	
Meat and poultry (in kg)	$-$ 0.533	$-$ 0.603	$-$ 1.11	0.479	0.041	$-$ 0.089	$-$ 0.12	$-$ 25.5
	(0.686)	(0.66)	(0.701)	(0.749)	(0.75)	(0.824)	(0.825)	
Dairy (in kg)	1.554	1.495	0.733	1.877	1.147	0.911	0.583	20.6
	(1.139)	(1.127)	(1.053)	(1.219)	(1.112)	(1.383)	(1.249)	
Vodka and liquors (in litres)	$-$ 0.031	$-$ 0.038	$-$ 0.061	0.02	$-$ 0.002	0.009	$-$ 0.002	$-$ 24.5
	(0.127)	(0.125)	(0.125)	(0.144)	(0.146)	(0.166)	(0.168)	
Refined sugar (in kg)	0.441	0.481	0.474	0.685	0.359	0.681	0.794	36.3
	(0.501)	(0.502)	(0.498)	(0.535)	(0.519)	(0.584)	(0.556)	
Candy and high$-$ sugar treats (in kg)	0.174	0.177	0.034	0.429	0.317	0.327	0.241	8.3
	(0.373)	(0.372)	(0.397)	(0.433)	(0.449)	(0.481)	(0.496)	
Starches (in kg)	0.149	0.222	$-$ 1.031	1.477	$-$ 1.204	$-$ 0.551	$-$ 0.944	$-$ 2.5
	(1.978)	(1.982)	(1.794)	(2.231)	(1.947)	(2.453)	(2.101)	
*Household monthly expenditure* *(by category, inflation-adjusted Rub)*								
Clothes (in Rub)	$-$ 2.043	$-$ 2.069	$-$ 1.556	$-$ 1.368	$-$ 1.147	$-$ 1.393	$-$ 1.366	$-$ 38.4
	(0.902)**	(0.9)**	(0.798)*	(1.065)	(0.937)	(1.192)	(1.027)	
Essential food items (in Rub)	0.804	0.644	$-$ 0.17	3.248	2.224	2.796	2.731	4.8
	(1.383)	(1.353)	(1.345)	(1.559)**	(1.55)	(1.717)	(1.721)	
Utilities (in Rub)	0.883	0.756	1.004	1.588	1.939	1.346	1.557	27.9
	(0.981)	(0.915)	(0.934)	(1.056)	(1.009)*	(1.197)	(1.152)	
Essential services (in Rub)	0.166	0.11	0.144	$-$ 0.049	0.078	0.157	0.033	5.7
	(0.879)	(0.873)	(0.756)	(0.86)	(0.859)	(0.916)	(0.923)	
All basic expenditure (in Rub)	$-$ 0.189	$-$ 0.558	$-$ 0.579	3.419	3.094	2.905	2.955	$-$ 2.3
	(2.583)	(2.449)	(2.368)	(2.736)	(2.508)	(3.061)	(2.814)	
All non-essential expenditure (in Rub)	$-$ 0.664	$-$ 0.634	0.362	3.07	3.79	4.092	4.052	$-$ 6.5
	(1.533)	(1.537)	(1.664)	(2.214)	(2.11)*	(2.272)*	(2.298)*	
Purchase of durable goods	$-$ 0.005	$-$ 0.007	$-$ 0.033	0.056	0.051	0.089	0.062	$-$ 3.6
	(0.119)	(0.12)	(0.117)	(0.127)	(0.125)	(0.143)	(0.142)	
*Saving, borrowing, and lending*								
Household savings in last 30 days (in 2011 prices)	$-$ 0.516	$-$ 0.553	$-$ 0.031	0.522	0.942	0.804	0.557	$-$ 20.8
	(1.025)	(1.04)	(1.37)	(1.315)	(1.476)	(1.468)	(1.551)	
Savings will last several months	$-$ 0.118	$-$ 0.119	$-$ 0.084	$-$ 0.075	$-$ 0.069	$-$ 0.016	$-$ 0.023	$-$ 49.9
	(0.102)	(0.102)	(0.104)	(0.108)	(0.106)	(0.12)	(0.119)	
Loan payments in last 30 days (in 2011 prices)	3.336	3.307	3.281	2.251	2.61	3.16	3.204	86.6
	(1.893)*	(1.888)*	(1.852)*	(1.94)	(1.85)	(2.191)	(2.125)	
Consumer loan in last 12 months	0.007	0.012	$-$ 0.01	$-$ 0.02	$-$ 0.023	0.037	0.025	1.8
	(0.087)	(0.086)	(0.089)	(0.1)	(0.101)	(0.11)	(0.111)	
Borrowed from a private individual in last 12 months	0.202 $^{(b)}$	0.206$^{(b)}$	0.2$^{(b)}$	0.139	0.132	0.124	0.116	331
	(0.058)***	(0.058)***	(0.06)***	(0.061)**	(0.062)**	(0.068)*	(0.07)*	
Loan to a private individual in last 12 months	$-$ 0.096	$-$ 0.097	$-$ 0.099	$-$ 0.101	$-$ 0.102	$-$ 0.102	$-$ 0.101	$-$ 88.8
	(0.082)	(0.081)	(0.082)	(0.088)	(0.087)	(0.102)	(0.101)	
*Housing characteristics*								
Household owns home	$-$ 0.059	$-$ 0.055	$-$ 0.075	0.086	0.055	0.033	0.01	$-$ 7.5
	(0.086)	(0.085)	(0.078)	(0.092)	(0.087)	(0.104)	(0.099)	
Hot water supply	0.048	0.033	$-$ 0.012	0.036	0.09	0.107	0.117	3.9
	(0.126)	(0.123)	(0.102)	(0.131)	(0.112)	(0.147)	(0.124)	
Central sewerage	0.124	0.106	0.105	0.192	0.257$^{(b)}$	0.211	0.257$^{(b)}$	17.7
	(0.125)	(0.116)	(0.053)**	(0.126)	(0.077)***	(0.141)	(0.084)***	
Central heating	0.115	0.1	0.072	0.099	0.142	0.145	0.181	16.4
	(0.125)	(0.119)	(0.07)	(0.127)	(0.079)*	(0.142)	(0.084)**	
*Age controls*	NO	YES	YES	YES	YES	YES	YES	
*Other controls*	NO	NO	YES	NO	YES	NO	YES	
*Linear trend*	YES	YES	YES	YES	YES	YES	YES	
*Quadratic trend*	NO	NO	NO	YES	YES	YES	YES	
*Cubic trend*	NO	NO	NO	NO	NO	YES	YES	
*RLMS year controls*	YES	YES	YES	YES	YES	YES	YES	
Num. obs.	328	328	328	727	727	1047	1047	

***− 1 % sign., **− 5% sign., *− 10% sign. Coefficient std. errors are given in parentheses under the coefficient. Error terms are clustered at regional level. Relative effect sizes are calculated relative to sample means in MC-ineligible families, based on average effects in models with a 12-month bandwidth

First, the MC eligibility may have affected the dynamics of household financial decisions. Since the MC could and was frequently used as a mortgage downpayment, it can be expected that a certain percentage of MC-eligible households started accumulating savings to afford a mortgage or even make a direct real estate purchase/exchange. However, after the bank loan/mortgage was taken, households may have had different financial goals and constraints. To meet financial obligations to respective banking institutions, these households would be more likely to resort to borrowing money from private individuals (e.g. family, friends) and, on the other hand, would be less likely to either lend money themselves or hold significant savings before the loan/mortgage is paid off. An outstanding loan/mortgage is also expected to affect household consumption and spending. For example, a higher bank debt burden may push the household toward making more economical decisions. In terms of spending and diets, it may translate into higher consumption of basic and less expensive food items, lower expenses on clothing, and lower non-essential expenditures.

It is known that nutrition has a profound effect on human health. The current evidence base shows that nutritional deficiencies in early childhood are associated with a range of negative consequences, including lower child IQ (Nyaradi et al., 2014), increased somatic morbidity (Uauy et al., 2008), and a higher risk of developing mental health disorders in adulthood (Muscaritoli, 2021). Although dietary guidelines are particularly notorious for being prone to change, there is a general consensus that consumption of certain nutrition groups (notably, vegetables and fruit) is associated with better health outcomes. In contrast, prolonged and excessive consumption of certain other nutrition groups, such as refined sugar, high-sugar treats, and processed foods, leads to an increased long-term risk of morbidity and illness.

The RLMS survey provides extraordinarily detailed information on self-reported household consumption of foods and food categories. I aggregated these variables into eight major consumption groups: vegetables/legumes (both fresh and canned), fruit (both fresh and canned), meat and poultry, dairy (including milk, butter, and cheese), refined sugar, high-sugar treats (tarts, candies, caramels, chocolate), starches (i.e. high-glycaemic crops and foods, such as bread, potatoes, pasta, buckwheat). The last included group—consumption of high-alcohol beverages (e.g. vodka, rum)—was assumed to be destined only for adult consumption and reflects the expenses made by heads of the household for their recreation or sustaining their habits.

Outcomes reflecting household general spending behaviour can be broadly divided into essential goods (essential food items, clothes, essential services, fuel, and utilities), non-essential (luxury) expenditures, and financial outcomes (household savings and reported loan payments). These measures reflect monthly reported household spending (inflation-adjusted, in 2011 prices) on each of these categories. In addition, I include an indicator variable for purchasing a durable good by surveyed adult household members within three months prior to their RLMS interview. It is worth noting that while RLMS provided information on the total household consumption, no data is available on how much was consumed by the child individually. Thus, this analysis relies on the assumption that household spending and dietary habits have a spillover effect on children, although the precise magnitude of such influence is difficult to evaluate for each participating family. A more detailed description of these and other variables is provided in Appendix 1.

Finally, I also analyse a set of variables reflecting household housing conditions. The MC subsidy may have significantly facilitated housing repairs or the provision of basic conveniences, such as centralised hot water supply, heating, and sewerage. It is expected that MC-eligible households needing such services were more likely to afford them with the financial support provided through the MC subsidy.

The RDD estimates of MC impact on household dietary and spending outcomes are presented in Table 4. Overall, the results indicate no robustly statistically significant differences between MC-eligible and MC-ineligible families for most outcomes. However, although statistically insignificant, models for certain food categories yield consistently stable $\beta$ values. In particular, MC-eligible households tend to report a lower vegetable consumption and a higher consumption of refined sugar and sugar treats, which is consistent with a lower quality diet.

As for spending categories, MC-eligible households, in general, report lower spending on clothing (1.3-2K Rub/40-65 USD reduction) and potentially higher spending on essential food items (by 1-3K Rub/30-100 USD), which is more consistent with the hypothesis of a sparing consumption profile observed under the circumstances of an outstanding financial obligation.

Table 4 reports the MC eligibility effect on household financial outcomes and housing conditions. Consistent with the active loan hypothesis, on average MC-eligible households report higher bank loan payments and a higher probability of borrowing money from a private individual in the past 12 months. Results for household loan payments expressed as a share in the total household income are reported in Table 23 in Appendix 3 and provide similar findings. Although statistically insignificant, models also produce stable $\beta$ coefficients pointing to a lower probability of high-volume savings and a lower likelihood of lending money to private individuals in MC-eligible families.

As far as housing conditions are concerned, evidence suggests that at least some households used their MC subsidy toward improving housing conditions by ensuring access to central heating and sewerage in their housing. Of note, some coefficients (i.e. borrowing from a private individual and the presence of central sewerage in the house in select models) stand the test of 5% statistical significance even after the Bonferroni correction for multiple outcome testing.

## Heterogeneity Analysis

### By Child Gender

The MC subsidy could have a differential effect on sub-populations. The fact that the MC subsidy could be spent on improving housing conditions makes this reform similar to the widely known family relocation program called Moving to Opportunity (MTO) which was implemented in several major US cities. Two influential studies on the effect of MTO by Kling et al. (2005, 2007) found that housing vouchers allowing participating families to relocate to less poverty-affected areas had a differential impact on children participating in the experiment. In particular, the authors concluded that young females benefited substantially from MTO participation in terms of their mental health, education, and engagement in risky activities. However, these positive effects on girls were offset by a nearly identical adverse MTO impact on boys.

**Table 5:** Regression discontinuity estimates of the MC impact on the second child outcomes (36 months’ window at 1 January 2007 cut-off birth date)

Outcome	Female			Male			Test $\beta _f=\beta _{m}$
	RDD 12m	RDD 24m	RDD 36m	RDD 12m	RDD 24m	RDD 36m	
*Child health outcomes*							
In good/excellent health	0.158	0.098	0.199	$-$ 0.194	0.063	$-$ 0.024	(0.048)**
	(0.098)	(0.083)	(0.126)	(0.154)	(0.115)	(0.167)	
Health score (1-highest, 5-lowest)	$-$ 0.217	$-$ 0.139	$-$ 0.241	0.183	$-$ 0.133	0.054	(0.049)**
	(0.119)*	(0.098)	(0.153)	(0.162)	(0.123)	(0.177)	
Num. of chronic conditions	$-$ 0.405$^{(b)}$	$-$ 0.163	$-$ 0.485$^{(b)}$	0.15	0.109	$-$ 0.06	(0.021)**
	(0.139)***	(0.122)	(0.185)***	(0.236)	(0.176)	(0.282)	
Health problem in last 30d	0.168	0.059	0.252	0.008	$-$ 0.117	$-$ 0.045	(0.466)
	(0.162)	(0.11)	(0.194)	(0.206)	(0.13)	(0.244)	
*Time spending*							
Extracurricular study (in mins per week)	$-$ 0.199	6.659	15.156	29.289	18.482	7.326	(0.552)
	(9.926)	(8.553)	(11.825)	(26.564)	(15.615)	(23.961)	
Extracurricular arts (in mins per week)	151.307	54.92	241.799$^{(b)}$	$-$ 34.223	7.518	$-$ 50.42	(0.029)**
	(60.564)**	(50.891)	(69.899)***	(62.072)	(49.495)	(75.335)	
Extracurricular sports (in mins per week)	57.835	$-$ 66.802	54.03	$-$ 140.131	$-$ 165.456	$-$ 81.786	(0.419)
	(96.027)	(82.063)	(108.249)	(198.18)	(150.22)	(222.188)	
Total productive time (in mins per week)	208.942	98.218	310.986	$-$ 176.136	$-$ 226.738	$-$ 122.114	(0.161)
	(122.427)*	(120.728)	(141.242)**	(226.674)	(244.048)	(246.745)	
Watching TV/on Internet (in mins per week)	13.166	$-$ 10.101	10.798	2.753	24.022	$-$ 0.483	(0.76)
	(24.75)	(21.061)	(30.482)	(37.387)	(27.473)	(42.378)	
Used a personal computer	0.094	$-$ 0.014	0.272	0.306	0.121	0.266	(0.5)
	(0.184)	(0.126)	(0.197)	(0.264)	(0.18)	(0.248)	
*Child well-being outcomes*							
Vacation with parent in 1yr	$-$ 0.232	$-$ 0.278	0.022	0.33	0.027	0.522	(0.033)**
	(0.171)	(0.111)**	(0.188)	(0.177)*	(0.134)	(0.21)**	
Num. of excursions/exhibition visits in 1yr	$-$ 6.074	$-$ 1.967	$-$ 4.616	1.193	1.363	$-$ 0.196	(0.028)**
	(2.405)**	(1.465)	(2.894)	(1.801)	(1.308)	(2.083)	
Sees friends $>2$ times per week	$-$ 0.046	$-$ 0.114	$-$ 0.272	$-$ 0.451	$-$ 0.135	$-$ 0.376	(0.199)
	(0.171)	(0.113)	(0.201)	(0.178)**	(0.124)	(0.208)*	
Received childcare in past 7 days	$-$ 0.115	$-$ 0.182	$-$ 0.185	0.261	0.163	0.326	(0.088)*
	(0.133)	(0.095)*	(0.166)	(0.181)	(0.118)	(0.196)*	
Received childcare from a relative in past 7 days	$-$ 0.138	$-$ 0.129	$-$ 0.168	0.069	0.047	0.273	(0.229)
	(0.1)	(0.074)*	(0.137)	(0.131)	(0.094)	(0.144)*	
Num of. obs	173	390	543	155	337	504	

***− 1 % sign., **− 5% sign., *− 10% sign. Reported RDD models use specifications with age controls, linear (12- and 24-month windows) and quadratic (36-month window) birth timeline, and time controls. Error terms are clustered at the regional level. Test column shows p-values for the test on equality of coefficients in the RDD model with the 12-month time window

In this subsection, I investigate whether MC eligibility affected boys and girls differently with respect to their health, development, and well-being outcomes. Table 5 and Appendix 3 of this paper provide a summary of estimation results.

Overall, boys and girls appear to have reacted differently to the MC program eligibility in several outcomes. In line with Kling et al. (2005, 2007), I find that girls may have experienced an improvement in their health status. In particular, girls saw an average 0.14–0.24 improvement in their health score and a 0.16–0.48 reduction in the reported number of chronic health conditions. Tests on the equality of $\beta$ coefficients provided in the last column of Table 5 reveal that these differences between girls and boys are statistically significant at 5% level. Surprisingly, girls in MC-eligible families were reported to go to fewer cultural events while spending significantly more time on extracurricular arts (an additional 1–4 hours per week).

While both boys and girls may have experienced a decline in the number of social contacts with their peers, heterogeneity analysis suggests that boys were more strongly affected by it. I hypothesise that a certain proportion of households used the MC as a mortgage downpayment and moved to a new residential address. It appears that boys had a harder time finding new peer networks and substituted previous peer relationships with stronger familial connections and formal childcare. This may be reflected by a significant increase in the share of MC-eligible families where children spent their vacation with parents and a 15-30 point increase in the percentage of children who reportedly received any childcare in the past seven days. Conversely, the need to provide external childcare, both formal and through another family member, appears to have decreased for girls by 10–20 percentage points.

### By Rural/Urban Areas

The nature of the MC subsidy makes it likely that different Russian regions were impacted differentially by this reform. Insofar as the size of the MC certificate—around 10,000 USD in 2007 prices—was not adjusted for local price levels, this subsidy had vastly different purchasing powers across Russia (see Sect. 2 for more context). In particular, concerning fertility effects of the MC, this point was addressed by Sorvachev and Yakovlev (2020), who showed a more sizable impact of the MC in regions with a higher ratio of subsidy size to regional housing prices. While a limited number of observations the RMLS data does not allow me to carry out the analysis at the regional level, I approximate the difference between low- and high-cost areas by examining Russian rural and urban areas. Results for main parametric RDD models (12- and 36-month time window) are summarised in Table 6, and complementary estimates are presented in Tables 25, 26, and 27 in Appendix 3.

**Table 6:** Regression discontinuity estimates of the MC impact (12 and 36 months’ window at 1 January 2007 cut-off), by rural/urban residence and poverty status

Outcome	Rural		Urban		Test $\beta _r= \beta _{u}$	Poor		Not poor		Test $\beta _p= \beta _{n}$
	RDD12m	RDD36m	RDD12m	RDD36m		RDD12m	RDD36m	RDD12m	RDD36m	
*Child’s outcomes*										
In good/excellent health	0.212	0.252	$-$ 0.144	$-$ 0.095	(0.058)*	0.021	$-$ 0.004	$-$ 0.065	$-$ 0.016	(0.675)
	(0.146)	(0.168)	(0.119)	(0.144)		(0.179)	(0.212)	(0.117)	(0.122)	
Health score (1-highest, 5-lowest)	$-$ 0.21	$-$ 0.21	0.103	0.085	(0.119)	$-$ 0.069	0.041	0.079	0.013	(0.49)
	(0.154)	(0.176)	(0.13)	(0.166)		(0.183)	(0.221)	(0.131)	(0.144)	
Num. of chronic conditions	$-$ 0.362	$-$ 0.522	0.051	$-$ 0.315	(0.138)	$-$ 0.213	$-$ 0.428	$-$ 0.059	$-$ 0.091	(0.649)
	(0.211)*	(0.223)**	(0.184)	(0.24)		(0.29)	(0.31)	(0.174)	(0.214)	
Health problem in last 30d	$-$ 0.087	$-$ 0.049	0.154	0.226	(0.33)	0.048	0.067	0.103	0.109	(0.845)
	(0.197)	(0.241)	(0.15)	(0.177)		(0.216)	(0.24)	(0.144)	(0.181)	
School homework/assignments (in mins per week)	$-$ 100.723	$-$ 126.25	44.5	24.975	(0.269)	$-$ 262.869	$-$ 183.016	142.673	122.878	(0.007)***
	(117.241)	(113.313)	(75.899)	(80.276)		(130.983)**	(141.958)	(71.412)**	(73.105)*	
Extracurricular study (in mins per week)	24.04	5.465	$-$ 8.097	22.544	(0.151)	33.372	14.065	5.069	15.199	(0.356)
	(18.301)	(9.31)	(14.578)	(20.748)		(26.406)	(17.045)	(17.08)	(20.605)	
Extracurricular arts (in mins per week)	20.401	82.958	50.158	73.116	(0.739)	11.433	70.779	78.107	144.127	(0.509)
	(47.028)	(67.753)	(66.515)	(75.195)		(93.751)	(101.389)	(60.909)	(70.617)**	
Total productive time (in mins per week)	163.093	127.622	127.148	129.693	(0.836)	384.231	290.642	123.296	234.907	(0.404)
	(158.098)	(201.813)	(155.745)	(174.587)		(242.244)	(244.754)	(154.867)	(176.473)	
Watching TV/on Internet (in mins per week)	2.425	32.464	23.79	8.884	(0.51)	64.834	102.815	$-$ 20.689	$-$ 38.531	(0.106)
	(31.897)	(36.061)	(25.129)	(31.878)		(43.005)	(50.986)**	(25.851)	(28.932)	
Used a personal computer	$-$ 0.125	0.042	0.235	0.235	(0.182)	0.232	0.158	0.076	0.245	(0.617)
	(0.225)	(0.252)	(0.17)	(0.19)		(0.263)	(0.29)	(0.194)	(0.195)	
Vacation with parent in 1yr	$-$ 0.087	0.195	0.053	0.285	(0.588)	0.129	0.246	$-$ 0.024	0.32	(0.555)
	(0.216)	(0.246)	(0.138)	(0.171)*		(0.223)	(0.28)	(0.145)	(0.151)**	
Num. of excursions/exhibition visits in 1yr	$-$ 2.244	$-$ 2.468	$-$ 5.715	$-$ 3.983	(0.216)	$-$ 5.131	$-$ 6.02	$-$ 2.762	$-$ 0.684	(0.527)
	(1.12)**	(1.349)*	(2.554)**	(3.308)		(2.981)*	(3.56)*	(2.362)	(2.546)	
Sees friends $>2$ times per week	$-$ 0.145	$-$ 0.172	$-$ 0.3	$-$ 0.477	(0.512)	$-$ 0.117	$-$ 0.308	$-$ 0.28	$-$ 0.362	(0.553)
	(0.181)	(0.216)	(0.154)*	(0.18)***		(0.21)	(0.25)	(0.154)*	(0.181)**	
Received childcare in past 7 days	0.173	0.076	$-$ 0.021	$-$ 0.001	(0.371)	0.276	0.291	0.019	0.035	(0.266)
	(0.154)	(0.184)	(0.153)	(0.186)		(0.167)	(0.193)	(0.147)	(0.168)	
*Household diet consumption*										
Vegetables/legumes (in kg)	$-$ 1.962	$-$ 3.655	$-$ 0.096	$-$ 0.304	(0.578)	$-$ 2.206	$-$ 3.999	$-$ 0.967	$-$ 1.027	(0.778)
	(3.219)	(4.013)	(1.229)	(1.499)		(3.588)	(4.262)	(1.012)	(1.348)	
Fruit (fresh and canned) (in kg)	$-$ 1.906	$-$ 3.856	$-$ 0.013	1.57	(0.399)	$-$ 1.748	$-$ 1.445	0.003	0.015	(0.425)
	(2.035)	(1.976)*	(0.951)	(1.255)		(1.676)	(2.069)	(1.34)	(1.444)	
Meat and poultry (in kg)	$-$ 2.649	$-$ 2.766	0.675	1.345	(0.017)**	1.064	2.104	$-$ 1.24	$-$ 1.085	(0.11)
	(1.071)**	(1.412)*	(0.787)	(1.032)		(1.276)	(1.448)	(0.857)	(1.078)	
Dairy (in kg)	1.355	$-$ 0.158	1.027	0.962	(0.859)	3.091	1.882	0.911	0.085	(0.328)
	(1.648)	(1.866)	(1.41)	(1.754)		(1.686)*	(1.926)	(1.478)	(1.676)	
Vodka and liquors (in litres)	$-$ 0.009	0.131	$-$ 0.048	$-$ 0.112	(0.856)	0.153	0.164	$-$ 0.154	$-$ 0.106	(0.133)
	(0.048)	(0.113)	(0.201)	(0.279)		(0.089)*	(0.188)	(0.183)	(0.236)	
Refined sugar (in kg)	0.465	0.645	0.572	1.128	(0.863)	$-$ 0.417	0.647	0.864	0.24	(0.24)
	(1.037)	(1.143)	(0.425)	(0.515)**		(0.932)	(1.027)	(0.67)	(0.732)	
Candy and high-sugar treats (in kg)	$-$ 0.388	$-$ 0.194	0.585	0.51	(0.207)	$-$ 0.214	0.237	0.313	0.46	(0.587)
	(0.661)	(0.883)	(0.432)	(0.607)		(0.855)	(1.087)	(0.376)	(0.553)	
Starches (in kg)	0.021	1.367	0.689	$-$ 1.62	(0.878)	$-$ 3.864	2.036	2.473	$-$ 3.309	(0.141)
	(3.818)	(3.862)	(2.064)	(2.486)		(3.186)	(3.614)	(2.707)	(2.976)	
*Household consumption and spending*										
Essential food items (in Rub)	$-$ 1.316	$-$ 0.435	2.021	5.238	(0.278)	0.928	4.665	0.157	0.596	(0.665)
	(2.552)	(3.283)	(1.627)	(2.087)**		(2.46)	(2.776)*	(1.644)	(2.13)	
Clothes (in Rub)	$-$ 1.034	$-$ 0.643	$-$ 2.662	$-$ 1.305	(0.35)	$-$ 1.778	$-$ 1.38	$-$ 2.035	$-$ 1.956	(0.864)
	(1.18)	(1.442)	(1.234)**	(1.415)		(1.367)	(1.588)	(1.177)*	(1.301)	
Utilities (in Rub)	$-$ 0.647	$-$ 0.893	1.574	2.854	(0.164)	1.503	0.902	0.868	2.036	(0.753)
	(0.563)	(0.817)	(1.425)	(1.775)		(0.965)	(0.955)	(1.398)	(1.766)	
Essential services (in Rub)	$-$ 2.14	$-$ 1.914	1.579	1.736	(0.024)**	1.605	0.579	$-$ 0.278	0.26	(0.337)
	(1.051)**	(1.404)	(1.23)	(1.222)		(1.771)	(2.023)	(0.838)	(0.901)	
All basic expenditure (in Rub)	$-$ 5.136	$-$ 3.885	2.512	8.523	(0.125)	2.252	4.768	$-$ 1.288	0.935	(0.443)
	(3.492)	(4.3)	(3.345)	(3.773)**		(3.833)	(4.097)	(3.396)	(3.767)	
All non-essential expenditure (in Rub)	$-$ 0.553	0.473	$-$ 0.695	5.341	(0.984)	0.356	5.396	0.218	5.073	(0.967)
	(2.193)	(3.603)	(2.315)	(3.165)*		(1.806)	(5.131)	(2.368)	(3.398)	
*Saving, lending, and borrowing*										
Household savings in last 30 days (in 2011 prices)	$-$ 0.2	$-$ 0.552	$-$ 0.683	1.699	(0.837)	$-$ 3.851	$-$ 3.164	0.602	1.089	(0.181)
	(2.196)	(2.53)	(0.838)	(1.952)		(2.341)	(2.311)	(1.899)	(2.443)	
Savings will last several months	$-$ 0.383	$-$ 0.402	0.05	0.241	(0.054)*	$-$ 0.113	$-$ 0.181	$-$ 0.078	0.033	(0.219)
	(0.182)**	(0.211)*	(0.127)	(0.139)*		(0.175)	(0.198)	(0.132)	(0.153)	
Loan payments in last 30 days (in 2011 prices)	$-$ 0.013	$-$ 1.938	5.52	7.184	(0.109)	1.558	0.779	5.288	7.255	(0.247)
	(1.562)	(1.894)	(3.085)*	(3.33)**		(2.119)	(2.569)	(3.129)*	(3.596)**	
Consumer loan in last 12 months	$-$ 0.224	$-$ 0.08	0.166	0.123	(0.026)**	$-$ 0.085	$-$ 0.082	0.089	0.153	(0.337)
	(0.134)*	(0.174)	(0.114)	(0.145)		(0.175)	(0.219)	(0.109)	(0.138)	
Borrowed from a private individual in last 12 months	0.294	0.168	0.165	0.092	(0.327)	0.18	0.062	0.193$^{(b)}$	0.104	(0.968)
	(0.111)***	(0.127)	(0.067)**	(0.089)		(0.102)*	(0.121)	(0.07)***	(0.082)	
Loan to a private individual in last 12 months	$-$ 0.28	$-$ 0.282	0.042	0.057	(0.049)**	$-$ 0.177	$-$ 0.135	0.004	$-$ 0.056	(0.292)
	(0.136)**	(0.168)*	(0.093)	(0.105)		(0.143)	(0.182)	(0.101)	(0.128)	
*Housing characteristics*										
Household owns home	0.104	0.168	$-$ 0.143	$-$ 0.094	(0.171)	0.012	0.106	$-$ 0.111	$-$ 0.062	(0.56)
	(0.116)	(0.153)	(0.119)	(0.13)		(0.174)	(0.1895)	(0.099)	(0.126)	
Hot water supply	$-$ 0.272	$-$ 0.182	0.191	0.265	(0.028)**	0.157	$-$ 0.013	$-$ 0.026	0.144	(0.471)
	(0.149)*	(0.187)	(0.126)	(0.158)*		(0.226)	(0.236)	(0.153)	(0.153)	
Central sewerage	0.088	0.415$^{(b)}$	0.079	0.178	(0.952)	0.529$^{(b)}$	0.387	$-$ 0.039	0.228	(0.019)**
	(0.082)	(0.135)***	(0.058)	(0.085)**		(0.194)***	(0.149)***	(0.146)	(0.108)**	
Central heating	0.235	0.357	$-$ 0.035	0.073	(0.04)**	0.485	0.185	$-$ 0.046	0.167	(0.033)**
	(0.093)**	(0.129)***	(0.082)	(0.099)		(0.201)**	(0.153)	(0.149)	(0.107)	
Num. of observations	127	434	201	613		138	433	191	614	

***− 1 % sign., **− 5% sign., *− 10% sign. Reported RDD models use specifications with age controls, linear (12-month window) and quadratic (36-month window) birth timeline, and time controls. Error terms are clustered at the regional level. Test column shows p-values for the test on equality of coefficients in the RDD model with the 12-month time window

Heterogeneity analysis suggests that rural and urban households reacted differently to MC eligibility across several dimensions. Estimation results indicate reduced consumption amounts of meat and poultry (by around 2.5kg), fruit (by 2–4 kg), and possibly vegetables, reported by rural MC-eligible families. However, since analysed consumption volumes only reflect purchased amounts of these food categories, part of this decrease may be accounted for by subsistence farming in rural areas.

As for household financial behaviour, although MC-eligible rural households did not report increased bank loan payments, they were significantly more likely to borrow from private individuals and had a much lower likelihood of holding significant savings or lending money. This behaviour is consistent with using the MC subsidy to improve housing conditions by conducting housing repairs without resorting to significant bank loans. As for child outcomes, estimation results in Tables 6 and 25 in Appendix 3 suggest that observed health improvements are also driven primarily by rural households. Altogether, obtained estimates point to the possibility that improved access to basic conveniences (e.g. central heating and sewerage) may have played a positive role in preventing child morbidity. Of note, the descriptive statistics in Table 14 in Appendix 2 show a stark difference between rural and urban Russian households regarding access to basic conveniences and essential public utilities. Only about 20% of rural Russian households reported having access to hot water supply, central heating, and sewerage in their housing, compared to 80–90% of families living in urban areas.

As for urban MC-eligible households, RDD estimation results indicate a higher bank loan burden compared to MC-ineligible households, both in absolute terms and as a share of expenses in the total household income (see Tables 26 in Appendix 3). These families also tend to report higher spending shares on essential food items, basic expenditure, and utilities, as well as lower spending on clothes. In addition, MC-eligible urban households and a higher propensity to resort to borrowing from private individuals. This behaviour is consistent with a more sparing consumption type characteristic of a household having a significant outstanding financial liability, such as a mortgage.

### By Poverty Status

Households with different levels of material wealth can be expected to react differently to MC incentives. The fact that in most cases the MC subsidy size was insufficient to purchase real estate directly can render the benefits of the MC reform partly inaccessible to poorer households that cannot afford a mortgage and do not have enough savings to purchase real estate immediately. This possibility is studied in the present subsection, wherein the poverty status is self-reported by RLMS survey respondents, who were asked whether they experienced difficulty providing themselves with essential consumer goods in the last 12 months (see Appendix 1 for more details on the RMLS questionnaire). Results of the RDD model estimation are summarised in Tables 6 and 28, 29, and 30, in Appendix 3.

RDD estimates provide tentative evidence that poorer MC-eligible households made some adjustments to their diets. In particular, these households tended to report higher consumption of dairy (by 2–3 litres) and strong alcoholic beverages (by around 0.15 litres). Although generally statistically insignificant, estimated coefficients for wealthier households tended to be of the opposite sign.

As far as housing conditions are concerned, poorer MC-eligible families had a higher likelihood of living in dwellings equipped with central sewerage and central heating, which appears to be the result of investing the MC subsidy in improving housing conditions. Of note, poorer households were also more likely to borrow from private individuals than from banking institutions.

Children in poorer MC-eligible families also, on average, report more time spent watching TV/browsing the Internet for non-educational purposes than their MC-ineligible counterparts (by 40-100 mins per week). Wealthier MC-eligible households, on average, reported more problems with child socialisation proxied by seeing friends more than two times per week. I hypothesize that these problems may result from adaptation difficulties encountered by children whose families could have moved to a new residential address. Consistent with earlier estimation, wealthier MC-eligible households also report higher bank loan monthly payments (both absolute and expressed as a share of total household income) and higher rates of borrowing from private individuals than similar MC-ineligible families.

## Discussion and Conclusion

This study evaluates the impact of the Maternity Capital (MC) child subsidy of 250.000 Rub (7.150 euros or 10.000 USD, in 2007 prices) that was introduced on 1 January 2007. To be eligible, a woman had to give birth to/adopt a second or a subsequent child. The MC could be spent in a limited number of ways, namely to improve family housing conditions, sponsor children’s education/childcare, or invest it in the mother’s retirement fund.

Using RLMS individual and household representative panel surveys from 2010 to 2017, I test regression discontinuity models whose aggregate level estimates point to the possibility that children in MC-eligible households had a moderately lower incidence of chronic health conditions (by around 0.15, or more than 40 % compared to MC-ineligible families) and a lower percentage of children who were reported to see their friends at least three times a week (i.e. a more than 30 % decrease relative to MC-ineligible families). Additional effect heterogeneity analysis suggests that the estimated improvements in the health status may be driven primarily by female children and poorer, rural MC-eligible households who were more likely to report that their housing had basic conveniences and public utilities (e.g. central heating and sewerage). At the same time, the reduction in child socialisation was more pronounced in male children, as well as in MC-eligible urban and wealthier families whose consumption and spending profiles are more consistent with having a significant outstanding financial obligation (e.g. a mortgage).

As for the heterogeneity of the MC eligibility impact across different sociodemographic groups, the most important and visible distinctions tended to arise when comparing rural/urban and poor/wealthier households. In particular, MC-eligible rural households were significantly less likely than MC-ineligible families to report a high level of savings or resort to consumer loans. Instead, to obtain external funding, these families tended to turn to borrowing from private individuals. In the meantime, compared to MC-eligible rural households, MC-eligible families living in urban areas were more likely to have higher loan liabilities and higher savings. Such behaviour is suggestive of different financial goals and liabilities across these subpopulations: rural and poorer households exhibited behaviour that is consistent with a one-time financial investment (e.g. improving housing conditions by equipping the house with central heating and sewerage), while the behaviour of urban and wealthier households was more consistent with using the MC subsidy to take out a mortgage or save up for a downpayment.

Overall, MC-eligible families also tended to report higher volumes of monthly bank loan payments, a higher likelihood of borrowing money from private individuals, and a lower probability of acting as a lender. These results are consistent with the hypothesis that a significant percentage of MC-eligible households used their subsidy to improve housing conditions.

However, due to small sample sizes, the estimates obtained in the effect heterogeneity analysis are generally volatile with respect to functional form specifications, the choice of window width around the intervention cut-off date, and the inclusion of additional covariates. 5Thus, evidence from the heterogeneity analysis should be interpreted as tentative.

Another limitation of the empirical strategy consists in the possibility of mothers’ self-selection into the MC eligibility in specifications using wide time windows around the MC eligibility cut-off date. In particular, RDD models for covariates reported in Appendix 5 point to the possibility that self-selection might have occurred in specifications with a 24- and 36-month bandwidth based on income and the self-identified poverty status. To address these insignificantly concerns, when interpreting RDD estimation results, the specifications using a 12-month bandwidth were considered as the main ones, even despite a potentially lower efficiency of derived estimates and a lower statistical power of the associated t-tests due to restricted sample sizes.

Since RLMS data are of survey type where respondents tend to provide their subjective evaluations, these data are likely by various factors related to respondents’ personality traits, past experiences, and unobserved socio-economic characteristics. While not affecting the unbiasedness of RDD estimates, it likely brings additional noise to both outcome and covariate variables. This results in a decreased efficiency of obtained estimates.

The contribution of this paper is threefold. First, it concentrates on a middle-income transitioning country, for which little evidence regarding pro-natalist policies is currently available. Second, it features a number of outcomes reflecting child well-being and human capital formation that, to the best of my knowledge, no other study had attempted to investigate, including patterns of time spent by children. Lastly, the RLMS survey allowed me to incorporate household-level outcome variables that no other study had attempted to evaluate systematically, including patterns of household lending, borrowing, and housing quality.

Child and household outcomes analysed in his paper were likely to be affected by the MC eligibility through several channels. First, since the MC is known to be frequently used as a mortgage downpayment, the financial behaviour of some households is expected to be affected by an ongoing financial liability. For example, to meet financial obligations to respective banking institutions, these households could be more likely to resort to borrowing money from private individuals (e.g. family and friends) and, on the other hand, would be less likely to either lend money themselves. This channel plausibly affected urban MC-eligible populations more strongly with regard to their borrowing behaviour, inasmuch as they reported a higher level of credit liabilities and a higher propensity to take out loans both from financial institutions and from private lenders. In addition, an outstanding loan, mortgage, or a significant prospective investment are expected to push households—at least temporarily— toward making more economical decisions. In terms of household spending and diets, it may translate into higher consumption of basic and less expensive food items, lower expenses on clothing, and potentially lower general and non-essential expenditures. The estimation results provide tentative evidence of a more austere consumption profile observed in rural families whose spending likely diminished after becoming eligible for the MC, as evidenced by consistently negative, albeit generally insignificant, coefficients across most consumption groups.

Second, in purely financial terms, the MC provides a very sizable income supplement that can relax, at least in the short term, the household budget constraint in MC-eligible families. As a result, the affected families may respond to MC incentives by re-calibrating their household spending behaviour. In particular, MC eligibility could affect households’ spending decisions regarding the share of the resources to be invested in children’s human capital (e.g. child education and childcare) vs. family members’ short-term (e.g. current consumption) and long-term needs (e.g. investing in better housing conditions). As far as investment in child human capital is concerned, there is tentative evidence that wealthier families benefitted the most from the MC, insofar as they reported better children’s involvement in extracurricular arts and a longer time spent on scholarly activities at home.

Lastly, the MC reform was plausibly accompanied by a broader shift in social policy priorities. The Russian state stood behind some public campaigning aimed at encouraging child adoptions, as well as preventing alcoholism, household abuse, and other forms of deviant behaviour that are not uncommon in Russian families. In particular, the World Health Organization (2014) survey on the prevalence of adverse childhood experiences among young (aged 16–25) people in the Russian Federation estimated that the prevalence of physical abuse stood at 14%, emotional abuse—at 37.9%, physical neglect—at 53.3%, and emotional neglect—at 57.9%. A total of 17.5% respondents reported experiencing four or more adverse childhood events, which is significantly higher than figures reported in similar surveys conducted in Europe. It is plausible that improved housing conditions and better access to child care may have in part mitigated these societal issues, although the extent to which this channel affected child outcomes across different family demographics is challenging to evaluate.

The results of this study could be generalised to institutionally, demographically, and economically close countries, most notably Belarus. Arguably to a lesser extent, these conclusions can be used for policy recommendations in other largely similar countries, which, however, differed from Russia in terms of the type of state institutions (e.g. Poland, Hungary, and Bulgaria), prevalent societal norms with regard to family and religion (for instance, predominantly Muslim but economically similar Kazakhstan, Turkey, and Malaysia).

This study can be complemented in a number of ways. For example, additional outcomes related to family relationships can be investigated in more depth. Since the MC certificates are tied to mothers, this subsidy can provide a higher degree of their financial independence. Thus, MC stimuli can potentially affect such outcomes as the probability of divorce, spending on personal items, and the patterns of time spent by mothers and other household members.

## Figures and Tables

**Fig. 3:**
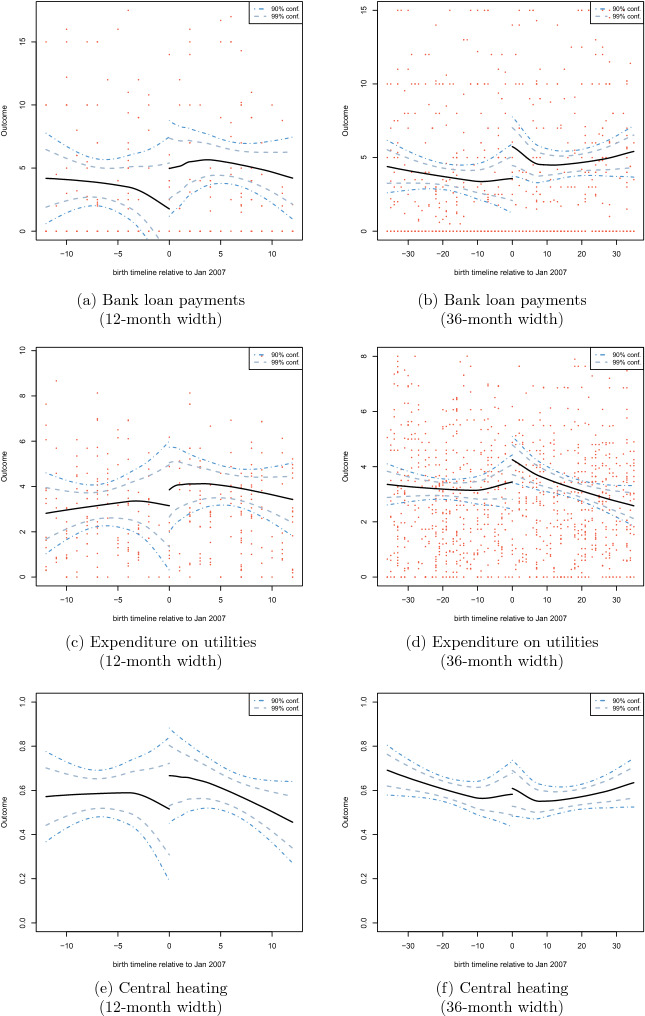
Maternity Capital effect on the 2nd child educational and time spending outcomes, nonparametric Lowess estimation

**Fig. 4:**
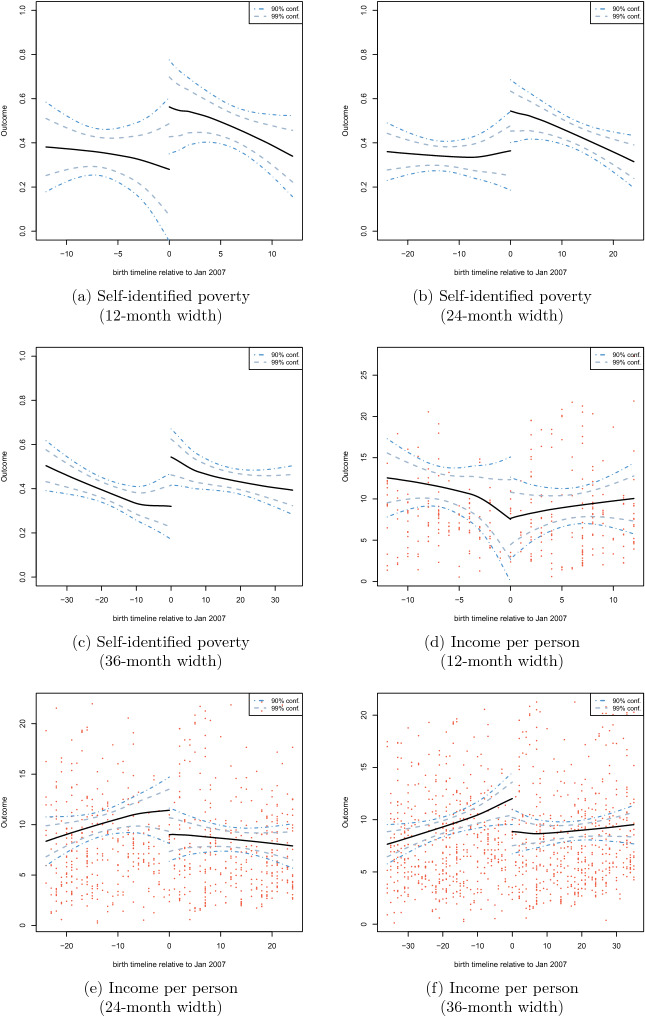
RLMS 2010–2017 characteristics of mothers having two children, nonparametric Lowess estimation of sample differences between mothers who gave the 2nd childbirth before and after January 2007

**Fig. 5:**
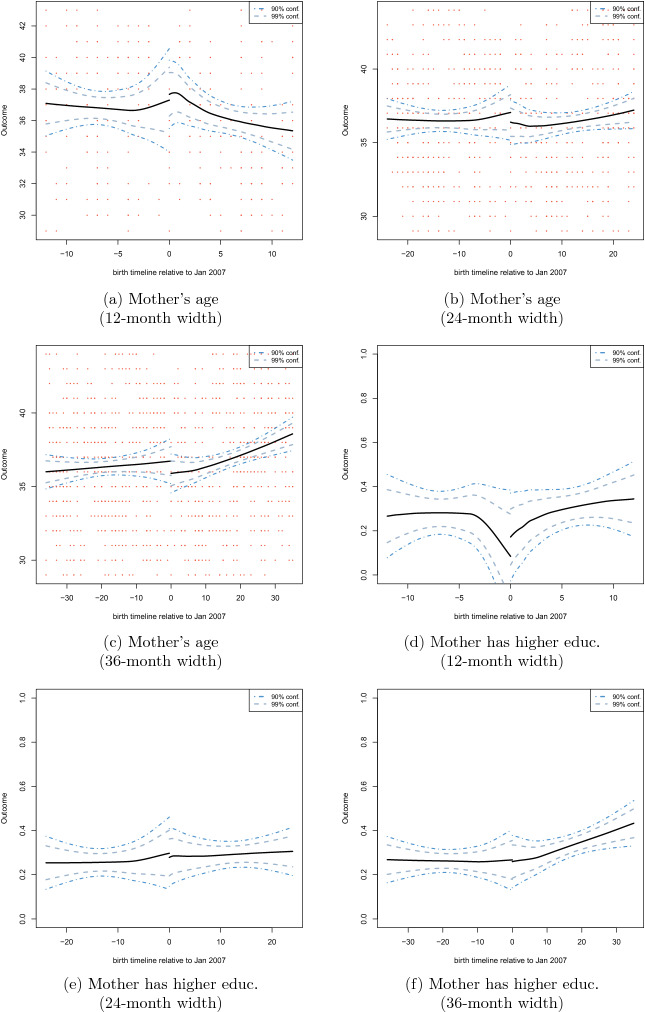
RLMS 2010–2017 characteristics of mothers having two children, nonparametric Lowess estimation of sample differences between mothers who gave the 2nd childbirth before and after January 2007

## Data Availability

Data used in this study are publicly available and can be downloaded from https://www.hse.ru/en/rlms/downloads. The code used in this study is re-deposited at https://github.com/alexproshin/RLMS.

## Appendix 1. RLMS Questionnaire

See Table 7.

**Table 7:** RLMS survey question on child and household outcomes

Outcome variable	RLMS survey question	Values
*Child health outcomes*		
Health score	How would evaluate health of your child? Is it	1- Very good 2- Good 3- Average, neither bad nor good 4- Bad 5- Very bad
Excellent/Good health	Idem. Note: value 1 if answers ’1’ or ’2’, 0 - otherwise	
Number of chronic conditions	Does your child have any chronic illness related to: heart, lungs, liver, kidneys, GI, spine, otorhinolaryngology, neurology, eyes, allergies or other chronic conditions	Sum of answers ’yes’
Health problems in last 30d	In the last 30 days, did your child have any health problems?	1- Yes, 2- No
*Child’s time spending patterns*		
School homework/assignments	How much time does your child spent on school assignments under parents’ or adult relatives’ supervision?	Time spent by parents or adult relatives, in minutes per week
Extracurricular study	Before or after school, how long does your child spend on: programming or computer technology, such as learning how to work with programs, programming languages and web technology -foreign language -other subjects, chosen by the parent or child, to get additional knowledge in, for example, math, biology, history	Time spent by child, in minutes per week
Extracurricular arts	Before or after school, does your child, how long does your child: -practice music or drawing/painting -practice dancing, photography, theatre and drama or other types of arts? -go to a young scientist club (in Russian: ’junyj tekhnik’), technical modelling club, naturalist club, practice handcraft, pottery, clay sculpting, or other types of technical and applied arts?	Idem
Extracurricular sports	Before or after school, does your child, how long does your child: –practice gymnastics, martial arts –play sport, practice athletic sports –practice other physical activity	Idem
Watching TV/on Internet	Before or after school, how long does your child: –watch TV, video, play video or computer games –browse the Internet or a local web network	Idem
*Child well-being*		
Used a personal computer	In the last 12 months, did your child use a personal computer for study?	1-Yes, 2-No
Vacation with parent in 1yr	In the last 12 months, did anyone among the child’s parents spend a vacation with him/her?	1-Yes, 2-No
Went to excursions/gallery	In the last 12 months, did your child go to a theatre, museum, exhibition, zoo, circus, or other cultural and entertainment events? (several RLMS questions for each activity type)	Sum of answers ’yes’
Sees friends $>2$ times a week	How often does your child meet his/her peers outside school or childcare facility? (Note: value 1 if answers ’4’ or ’5’, 0 - otherwise)	1: 1 to 3 times per month; 2: 1 time per week; 3: 2 times per week; 4: 3-4 times per week; 5: every day
Received childcare in past 7 days	In the last 7 days, has someone who is not a ember of your household provided childcare to your child? (includes friends, childcare facilities, nannies, relatives who life separately)	1-Yes, 2-No
*Household consumption*	How much of the following products did your household buy in the last 7 days?	
Vegetables/legumes	Cucumbers (including pickled), tomatoes (including pickled), beets, carrots, zucchini, pumpkin, and other vegetables.	In kilograms per week
Fruit (fresh and canned)	Watermelon, melon (including dried and pickled), fruit and berry conserves, fresh berries, fresh fruit, dried fruit and berries, nuts and seeds	Same
Meat and poultry	Meat preserves, fresh beef and veal, fresh mutton and goat meat, pork, animal giblets, and poultry	Same
Dairy	Milk preserves, powdered milk, fresh milk, sour milk products (kefir, yogurt etc.), sour cream and cream, cottage cheese, cheese	Same
Refined sugar	Refined sugar	Same
Candy and high-sugar treats	Ice cream, candy, chocolate, honey, jam, cookies, cakes, tarts, waffles, pryaniks, pastries	Same
Starches	White bread, rye bread, rice, pasta, potatoes, etc.	Same
Vodka and spirits (liquors)	Vodka, wine and liquors	In litres per week
*Household spending*		
Essential food items	Bread, starches, flour, pasta, vegetables (fresh and pickled), fruit (fresh, dried, and canned), nuts, meats (fresh and preserves), poultry, deli, dairy, eggs, vegetable oils, sugar, chocolate, honey, cookies, fish (fresh and preserves), other seafood products. Expenditures are calculated for a 1-month period	In Rub, inflation-adjusted (2011 prices)
Clothes	How much did you and other members of your household spend on clothes and shoes? (both adult and child clothes/shoes)	Same
Purchase of durable goods	In the last 3 months, did your household purchase: a TV, digital appliances, kitchen appliances, furniture, a car, a motorbike, a parking garage, real estate, a bike and or similar sports equipment	Same
Essential services	In the last 30 days, how much did your family spend on: public transport, tailor services, repairs of different appliances/devices, laundry, banya (sauna), hairdresser’s, postal services, phone and Internet bills, cable TV.	Same
All basic expenditure	Essential food items, clothing, utilities, essential services	Same
Non-essential expenditures	Alcoholic beverages, tobacco, personal vehicle, sports equipment, private classes, leisure travel, cultural events, perfume and cosmetics.	Same
*Household saving, borrowing, and lending*		
Bank loan payments	In the last 30 days, how much did your family spend on loan payments?	In Rub, inflation-adjusted (2011 prices)
Savings will last several months	If all members of your household lose all sources of their income, how long will your family be able to maintain your current standard of living, without cutting expenses or selling any of your property, and using only your personal savings? Value 1 of the respondent answers 1 or 2.	1-half a year or longer, 2- several months, 3- no more than one month, 4- no more than two weeks, 5- no more than one week, 6- not even one day
*Housing characteristics*		
Central sewerage	Cucumbers (including pickled), tomatoes (including pickled), beets, carrots, zucchini, pumpkin, and other vegetables.	In kilograms per week
Central heating	Watermelon, melon (including dried and pickled), fruit and berry conserves, fresh berries, fresh fruit, dried fruit and berries, nuts and seeds	Same

## Appendix 2. Descriptive Statistics

See Tables 8

**Table 8:** Distribution of observations in the analytical sample, by child’s year of birth

	All sample	MC eligible	MC ineligible
Birth 2004	168	0	168
Birth 2005	200	0	200
Birth 2006	147	0	147
Birth 2007	164	164	0
Birth 2008	194	194	0
Birth 2009	174	174	0
Total	1047	515	532

, 9

**Table 9:** Distribution of observations in the analytical sample, by year of interview

	All sample	MC eligible	MC ineligible
Year 2010	40	0	40
Year 2011	107	0	107
Year 2012	192	0	192
Year 2013	180	56	124
Year 2014	157	109	48
Year 2015	166	162	4
Year 2016	137	137	0
Year 2017	68	68	0
Total	1047	515	532

, 10

**Table 10:** Distribution of observations in the analytical sample, by RLMS wave

	All sample	MC eligible	MC ineligible
RLMS wave 2010	49	0	49
RLMS wave 2011	108	0	108
RLMS wave 2012	185	0	185
RLMS wave 2013	184	59	125
RLMS wave 2014	150	106	44
RLMS wave 2015	173	169	4
RLMS wave 2016	130	130	0
RLMS wave 2017	68	68	0
Total	1047	515	532

, 11

**Table 11:** Descriptive statistics (sample mean and standard deviation) for child, mother, and household characteristics, by year of birth of the 2nd child

Variable	Born in 2004	Born in 2005	Born in 2006	Born in 2007	Born in 2008	Born in 2009
*Child’ characteristics*						
Age	7.071	6.99	6.932	6.945	6.99	6.937
	(0.823)	(0.814)	(0.816)	(0.831)	(0.801)	(0.791)
Sex (male)	0.53	0.405	0.327	0.573	0.531	0.511
	(0.501)	(0.492)	(0.471)	(0.496)	(0.5)	(0.501)
Year	2011.232	2012.14	2012.993	2014.055	2015.113	2016.069
	(0.862)	(0.827)	(0.872)	(0.928)	(0.868)	(0.809)
*Mothers’ characteristics*						
Age	36.006	36.27	36.803	35.72	37.093	37.994
	(4.295)	(4.759)	(4.576)	(5.163)	(5.914)	(5.417)
Urban residence	0.667	0.49	0.626	0.616	0.464	0.69
	(0.473)	(0.501)	(0.486)	(0.488)	(0.5)	(0.464)
Single parent	0.125	0.16	0.177	0.146	0.144	0.121
	(0.332)	(0.368)	(0.383)	(0.355)	(0.352)	(0.327)
In good/excellent health	0.387	0.51	0.476	0.549	0.541	0.42
	(0.488)	(0.501)	(0.501)	(0.499)	(0.5)	(0.495)
Higher education diploma	0.274	0.24	0.279	0.287	0.304	0.42
	(0.447)	(0.428)	(0.45)	(0.454)	(0.461)	(0.495)
Ethnicity other than Russian	0.137	0.12	0.082	0.134	0.175	0.075
	(0.345)	(0.326)	(0.275)	(0.342)	(0.381)	(0.264)
Poverty	0.491	0.33	0.356	0.5	0.385	0.443
	(0.501)	(0.471)	(0.481)	(0.502)	(0.488)	(0.498)
Alcohol cons. $>1$ time per week	0.048	0.04	0.041	0.024	0.026	0.04
	(0.214)	(0.196)	(0.199)	(0.155)	(0.159)	(0.197)
Mothers subjective life satisfaction (1-lowest, 5-highest)	3.304	3.475	3.442	3.442	3.281	3.402
	(1.098)	(0.997)	(1.054)	(1.049)	(1.132)	(1.143)
*Household characteristics*						
Household income in last 30 days (in 2011 prices)	37.538	42.397	50.48	42.806	37.705	45.499
	(26.064)	(35.786)	(69.123)	(29.811)	(25.671)	(27.74)
Household savings in last 30 days (in 2011 prices)	1.43	2.169	1.76	1.22	0.564	1.126
	(5.198)	(8.528)	(12.782)	(7.167)	(2.641)	(7.392)
Savings will last several months	0.206	0.245	0.214	0.161	0.168	0.218
	(0.406)	(0.431)	(0.412)	(0.369)	(0.375)	(0.414)
Consumer loan in last 12 months	0.286	0.216	0.17	0.165	0.077	0.156
	(0.491)	(0.425)	(0.395)	(0.372)	(0.268)	(0.364)
Borrowed from a private individual in last 12 months	0.065	0.08	0.061	0.128	0.124	0.046
	(0.248)	(0.272)	(0.241)	(0.335)	(0.33)	(0.21)
Loan to a private individual in last 12 months	0.132	0.095	0.11	0.091	0.046	0.075
	(0.339)	(0.295)	(0.313)	(0.289)	(0.211)	(0.264)
Loan payments in last 30 days (in 2011 prices)	4.397	3.014	3.818	5.509	2.65	6.109
	(7.459)	(5.842)	(6.558)	(10.08)	(5.453)	(9.924)
Vegetables/legumes	2.495	1.927	2.277	2.744	1.806	2.604
	(3.891)	(3.57)	(4.061)	(5.054)	(3.818)	(4.31)
Fruit (fresh and canned)	4.057	3.847	3.673	3.545	3.213	3.581
	(4.97)	(3.884)	(3.647)	(3.456)	(3.573)	(3.684)
Meat and poultry	3.264	3.246	2.94	3.123	3.137	3.624
	(3.646)	(3.831)	(2.703)	(3.901)	(3.397)	(3.264)
Dairy	6.033	5.24	6.131	6.004	5.4	6.387
	(5.098)	(4.691)	(5.101)	(4.443)	(4.673)	(4.206)
Vodka and liquors (in litres)	0.166	0.226	0.177	0.134	0.11	0.197
	(0.728)	(0.661)	(0.537)	(0.437)	(0.431)	(0.686)
Refined sugar	0.983	1.467	1.282	1.263	1.688	1.565
	(1.647)	(2.355)	(2.339)	(1.725)	(2.844)	(2.309)
Candy and high-sugar treats	1.576	1.814	1.549	1.684	1.724	1.675
	(1.441)	(1.588)	(1.505)	(1.242)	(1.824)	(1.35)
Starches	9.71	11.192	8.979	10.928	10.553	9.67
	(7.577)	(11.432)	(8.05)	(9.136)	(9.273)	(8.675)
Essential food items	9.759	9.902	8.956	10.114	8.875	9.695
	(6.189)	(6.918)	(5.487)	(6.405)	(6.282)	(5.732)
Clothes	3.888	5.234	4.923	4.106	4.122	4.015
	(4.29)	(5.207)	(4.058)	(4.564)	(3.575)	(3.423)
All basic expenditure	19.122	20.472	19.503	20.567	18.038	18.928
	(10.014)	(11.811)	(9.932)	(12.912)	(9.759)	(8.873)
Non-essential expenditures	5.091	7.685	4.832	5.083	3.592	4.189
	(18.368)	(23.301)	(12.692)	(9.537)	(9.352)	(5.344)
Purchase of durable goods	0.423	0.395	0.415	0.341	0.309	0.351
	(0.495)	(0.49)	(0.494)	(0.476)	(0.463)	(0.479)
Utilities	3.499	2.949	3.162	4.087	2.76	2.925
	(2.873)	(3.062)	(3.115)	(5.279)	(2.303)	(1.983)
Essential services	1.977	2.387	2.462	2.26	2.28	2.294
	(1.626)	(3.815)	(3.094)	(3.691)	(5.23)	(3.074)
N	168	200	147	164	194	174

Sample standard deviations are in parentheses

, 12

**Table 12:** Descriptive statistics (sample mean and standard deviation) for child, mother, and household characteristics, by year of birth of the 2nd child (continued)

Variable	Born in 2004	Born in 2005	Born in 2006	Born in 2007	Born in 2008	Born in 2009
Sh. Essential food items	0.335	0.318	0.288	0.317	0.309	0.277
	(0.308)	(0.264)	(0.285)	(0.314)	(0.249)	(0.306)
Sh. Clothes	0.142	0.154	0.151	0.128	0.14	0.106
	(0.231)	(0.141)	(0.21)	(0.139)	(0.135)	(0.093)
Sh. Utilities	0.134	0.085	0.085	0.108	0.084	0.077
	(0.313)	(0.075)	(0.077)	(0.128)	(0.079)	(0.06)
Sh. Essential services	0.066	0.067	0.062	0.062	0.057	0.061
	(0.089)	(0.106)	(0.073)	(0.07)	(0.056)	(0.084)
Sh. All basic expenditure	0.678	0.623	0.586	0.615	0.59	0.522
	(0.74)	(0.361)	(0.458)	(0.461)	(0.334)	(0.412)
Sh. Non-essential expenditure	0.194	0.172	0.158	0.151	0.105	0.104
	(0.926)	(0.598)	(0.518)	(0.369)	(0.245)	(0.138)
Sh. Savings	0.029	0.037	0.028	0.027	0.018	0.014
	(0.1)	(0.14)	(0.13)	(0.2)	(0.1)	(0.076)
Sh. Loan payments	0.165	0.104	0.102	0.136	0.077	0.149
	(0.414)	(0.252)	(0.163)	(0.222)	(0.163)	(0.218)
Household owns home	0.849	0.934	0.837	0.878	0.851	0.971
	(0.359)	(0.248)	(0.371)	(0.328)	(0.357)	(0.168)
Hot water supply	0.677	0.49	0.585	0.573	0.474	0.655
	(0.469)	(0.501)	(0.494)	(0.496)	(0.501)	(0.477)
Central sewerage	0.737	0.565	0.633	0.604	0.505	0.713
	(0.442)	(0.497)	(0.484)	(0.491)	(0.501)	(0.454)
Central heating	0.69	0.545	0.585	0.598	0.474	0.649
	(0.464)	(0.499)	(0.494)	(0.492)	(0.501)	(0.479)
N	168	200	147	164	194	174

Sample standard deviations are in parentheses

, 13

**Table 13:** Descriptive statistics for child and mother demographic and socioeconomic characteristics, by RLMS wave

Variable	RLMS 2010	RLMS 2011	RLMS 2012	RLMS 2013	RLMS 2014	RLMS 2015	RLMS 2016	RLMS 2017
*Child’s characteristics*								
Age	6.02	6.472	6.951	7.043	6.9	6.965	7.377	7.824
	(0.143)	(0.52)	(0.803)	(0.842)	(0.817)	(0.792)	(0.626)	(0.384)
Sex (male)	0.469	0.463	0.449	0.435	0.48	0.549	0.508	0.515
	(0.504)	(0.501)	(0.499)	(0.497)	(0.501)	(0.499)	(0.502)	(0.503)
Year	2010.184	2011.093	2012.016	2013.038	2014	2015.04	2016	2017.015
	(0.391)	(0.291)	(0.127)	(0.192)	(0)	(0.198)	(0)	(0.121)
*Mothers’ characteristics*								
Age	35.306	35.667	36.427	36.522	36.22	36.624	37.923	38.794
	(4.244)	(4.449)	(4.53)	(4.944)	(5.337)	(5.445)	(5.722)	(5.27)
Urban	0.653	0.565	0.578	0.603	0.56	0.584	0.546	0.676
	(0.481)	(0.498)	(0.495)	(0.491)	(0.498)	(0.494)	(0.5)	(0.471)
Single parent	0.082	0.111	0.178	0.168	0.14	0.145	0.138	0.118
	(0.277)	(0.316)	(0.384)	(0.375)	(0.348)	(0.353)	(0.347)	(0.325)
In good/excellent health	0.408	0.5	0.438	0.484	0.54	0.509	0.508	0.382
	(0.497)	(0.502)	(0.497)	(0.501)	(0.5)	(0.501)	(0.502)	(0.49)
Higher education diploma	0.327	0.269	0.259	0.261	0.28	0.347	0.338	0.397
	(0.474)	(0.445)	(0.44)	(0.44)	(0.451)	(0.477)	(0.475)	(0.493)
Ethnicity other than Russian	0.061	0.111	0.124	0.114	0.153	0.127	0.154	0.059
	(0.242)	(0.316)	(0.331)	(0.319)	(0.362)	(0.334)	(0.362)	(0.237)
Poverty	0.531	0.38	0.38	0.418	0.426	0.413	0.431	0.426
	(0.504)	(0.488)	(0.487)	(0.495)	(0.496)	(0.494)	(0.497)	(0.498)
Alcohol cons. $>1$ time per week	0.02	0.065	0.049	0.033	0.02	0.017	0.038	0.059
	(0.143)	(0.247)	(0.216)	(0.178)	(0.14)	(0.131)	(0.193)	(0.237)
Mothers subjective life satisfaction (1-lowest, 5-highest)	3.265	3.296	3.47	3.527	3.315	3.32	3.341	3.471
	(1.114)	(1.079)	(1.069)	(0.94)	(1.103)	(1.158)	(1.1)	(1.139)
N	49	108	185	184	150	173	130	68

Sample standard deviations are in parentheses

, 14

**Table 14:** Descriptive statistics for all sample (RLMS 2011-2017 respondents with second children born between 2004 and 2010 and aged 6-8 years, in households with two children), by urban/rural residence

Variable	All sample				Rural		Urban	
	Mean	SD	Min	Max	Mean	SD	Mean	SD
*Child characteristics*								
Age	6.979	0.812	6	8	6.977	0.81	6.98	0.814
Sex (male)	0.481		0	1	0.495		0.471	
Year	2013.618	1.889	2010	2018	2013.601	1.869	2013.63	1.904
In good/excellent health	0.772		0	1	0.808**		0.747**	
Health score (1-excellent, 5-bad)	2.204	0.512	1	5	2.159**	0.456	2.235**	0.546
Health problem in last 30d	0.345		0	1	0.309**		0.37**	
Num. of chronic conditions	0.299	0.635	0	5	0.205***	0.473	0.365***	0.721
Extracurricular study	14.462	54.406	0	720	3.769***	27.577	22.958***	67.458
Extracurricular arts	120.048	195.486	0	1920	97.954***	204.627	137.518***	186.346
Extracurricular sports	219.844	336.195	0	2200	233.692	353.284	208.893	322.055
Watching TV/on Internet	121.78	85.596	0	960	108.877***	64.015	131.983***	98.284
Used a personal computer	0.667		0	1	0.532***		0.774***	
Vacation with parent in 1yr	0.672		0	1	0.525***		0.776***	
Num. of excursions/exhibition visits in 1yr	4.382	5.556	0	35	2.064***	3.501	6.047***	6.134
Sees friends $>2$ times per week	0.623		0	1	0.658*		0.598*	
Received childcare in past 7 days	0.329		0	1	0.276***		0.365***	
Received childcare from a relative in past 7 days	0.178		0	1	0.161		0.189	
*Mother characteristics*								
Age	36.655	5.119	25	53	35.848***	5.185	37.227***	4.998
Urban	0.585		0	1	0		1	
Single parent	0.145		0	1	0.131		0.155	
In good/excellent health	0.482		0	1	0.514*		0.46*	
Higher education diploma	0.3		0	1	0.164***		0.396***	
Ethnicity other than Russian	0.122		0	1	0.164***		0.093***	
Poverty	0.415		0	1	0.461**		0.383**	
Alcohol cons. $>1$ time per week	0.036		0	1	0.023*		0.046*	
Mothers subjective life satisfaction (1-lowest, 5-highest)	3.39	1.08	1	5	3.352	1.149	3.417	1.029
*Household consumption*								
Vegetables/legumes	2.285	4.124	0	30	1.478***	3.891	2.867***	4.192
Fruit (fresh and canned)	3.65	3.903	0	26	2.964***	3.901	4.129***	3.835
Meat and poultry	3.228	3.497	0	30	2.819***	3.848	3.517***	3.197
Dairy	5.835	4.711	0	33.3	4.347***	4.565	6.875***	4.533
Vodka and liquors (in litres)	0.169	0.593	0	8	0.101***	0.448	0.217***	0.673
Refined sugar	1.388	2.265	0	20	1.871***	2.864	1.053***	1.657
Candy and high-sugar treats	1.679	1.513	0	12	1.923***	1.786	1.507***	1.259
Starches	10.231	9.217	0	69	11.989***	10.756	8.992***	7.729
Essential food items	9.555	6.225	0	47.344	8.991**	6.684	9.953**	5.851
Clothes	4.389	4.266	0	38.185	4.586	3.691	4.249	4.629
All basic expenditure	19.427	10.664	0.78	73.632	16.937***	9.316	21.189***	11.201
All non-essential expenditure	5.121	14.715	0	224.183	4.254	17.759	5.735	12.085
Purchase of durable goods	0.371		0	1	0.334**		0.396**	
Utilities	3.207	3.26	0	32.175	1.866***	1.937	4.156***	3.651
Essential services	2.277	3.645	0	58.767	1.494***	2.759	2.83***	4.071
*Saving, lending, and borrowing*								
Household income in last 30 days (in 2011 prices)	42.462	37.75	0.5	672.303	30.675***	20.854	50.808***	44.268
Household savings in last 30 days (in 2011 prices)	1.374	7.718	0	152.742	0.693***	3.513	1.855***	9.618
Savings will last several months	0.203		0	1	0.19		0.211	
Consumer loan in last 12 months	0.177		0	2	0.152*		0.195*	
Borrowed from a private individual in last 12 months	0.085		0	1	0.099		0.075	
Loan to a private individual in last 12 months	0.09		0	1	0.06***		0.111***	
Loan payments in last 30 days (in 2011 prices)	4.187	7.785	0	60	2.972***	5.21	5.047***	9.088
*Housing characteristics*								
Household owns home	0.889		0	1	0.894		0.885	
Hot water supply	0.571		0	1	0.219***		0.819***	
Central sewerage	0.621		0	1	0.254***		0.881***	
Central heating	0.586		0	1	0.198***		0.861***	
Number of observations	1047				434		613	

Stars denote p-values for t-tests on equality of means (non-binary variables)/chi-square tests on equality of proportions (for binary variables): ***− 1 % sign., **− 5% sign., *− 10% sign

, 15

**Table 15:** Correlation matrix for main variables, whole sample

Num variable		1	2	3	4	5	6	7	8	9	10	11	12	13	14
1	Age	1	0.048	0.355	$-$ 0.031	0.028	0.04	0.08	0.095	0.045	0.117	$-$ 0.006	0.032	0.059	0.166
2	Sex (male)	0.048	1	0.077	$-$ 0.053	0.039	0.024	0.001	0.209	0.037	0.047	$-$ 0.232	0.018	0.071	0.041
3	Year	0.355	0.077	1	0.038	$-$ 0.019	0.047	$-$ 0.075	0.067	0.048	0.022	$-$ 0.01	$-$ 0.071	0.018	$-$ 0.016
4	In good/excellent health	$-$ 0.031	$-$ 0.053	0.038	1	$-$ 0.899	$-$ 0.239	$-$ 0.359	$-$ 0.043	$-$ 0.158	0.04	0.042	0.061	0.006	$-$ 0.083
5	Health score (1-highest, 5$-$ lowest)	0.028	0.039	$-$ 0.019	$-$ 0.899	1	0.25	0.344	0.057	0.131	$-$ 0.039	$-$ 0.023	$-$ 0.073	0.015	0.077
6	Health problem in last 30d	0.04	0.024	0.047	$-$ 0.239	0.25	1	0.182	$-$ 0.039	0.041	0.067	0.005	0.006	0.09	0.06
7	Num. of chronic conditions	0.08	0.001	$-$ 0.075	$-$ 0.359	0.344	0.182	1	$-$ 0.076	0.096	0.03	0.06	$-$ 0.054	0.113	0.089
8	Bad/Satisfactory GPA	0.095	0.209	0.067	$-$ 0.043	0.057	$-$ 0.039	$-$ 0.076	1	0.021	$-$ 0.053	$-$ 0.111	$-$ 0.003	0	$-$ 0.056
9	School homework/assignments	0.045	0.037	0.048	$-$ 0.158	0.131	0.041	0.096	0.021	1	0.038	0.145	0.009	$-$ 0.081	0.023
10	Extracurricular study	0.117	0.047	0.022	0.04	$-$ 0.039	0.067	0.03	$-$ 0.053	0.038	1	0.076	$-$ 0.002	0.006	0.142
11	Extracurricular arts	$-$ 0.006	$-$ 0.232	$-$ 0.01	0.042	$-$ 0.023	0.005	0.06	$-$ 0.111	0.145	0.076	1	0.145	0.058	0.089
12	Extracurricular sports	0.032	0.018	$-$ 0.071	0.061	$-$ 0.073	0.006	$-$ 0.054	$-$ 0.003	0.009	$-$ 0.002	0.145	1	0.071	0.02
13	Watching TV/on Internet	0.059	0.071	0.018	0.006	0.015	0.09	0.113	0	$-$ 0.081	0.006	0.058	0.071	1	0.241
14	Used a personal computer	0.166	0.041	$-$ 0.016	$-$ 0.083	0.077	0.06	0.089	$-$ 0.056	0.023	0.142	0.089	0.02	0.241	1
15	Vacation with parent in 1yr	0.05	$-$ 0.073	0.028	$-$ 0.094	0.063	0.08	0.173	$-$ 0.163	0.156	0.099	0.176	$-$ 0.16	0.026	0.207
16	Num. of excursions/exhibition visits in 1yr	0.141	$-$ 0.018	0.1	$-$ 0.011	$-$ 0.04	0.127	0.142	$-$ 0.086	0.141	0.284	0.157	0.045	0.027	0.136
17	Sees friends $>2$ times per week	$-$ 0.049	0.053	$-$ 0.062	0.074	$-$ 0.077	$-$ 0.006	0.047	0.096	0.001	$-$ 0.083	$-$ 0.046	0.238	$-$ 0.079	$-$ 0.06
18	Received childcare in past 7 days	$-$ 0.035	$-$ 0.009	$-$ 0.017	0.009	$-$ 0.015	0.032	$-$ 0.03	$-$ 0.066	0.007	0.073	0.115	0.066	0.04	0.101
19	Received childcare from a relative in past 7 days	$-$ 0.046	0.015	0.023	0.011	$-$ 0.023	0.009	$-$ 0.002	$-$ 0.027	0.013	0.058	0.012	0.064	0.03	0.117
20	Mother’s age	0.038	$-$ 0.054	0.146	$-$ 0.062	0.064	0.058	0.047	$-$ 0.086	0.111	0.142	0.18	$-$ 0.064	0.078	0.083
21	Urban	0.083	$-$ 0.051	0.003	$-$ 0.053	0.031	0.111	0.119	0.023	$-$ 0.123	0.184	0.122	$-$ 0.027	0.152	0.252
22	Single parent	$-$ 0.035	0.085	$-$ 0.056	$-$ 0.006	0.022	0.009	$-$ 0.002	0.076	$-$ 0.058	$-$ 0.006	0.007	0.041	0.041	0.011
23	In good/excellent health m	$-$ 0.096	$-$ 0.001	$-$ 0.017	0.284	$-$ 0.295	$-$ 0.125	$-$ 0.153	0.021	$-$ 0.142	0.014	0.004	0.012	$-$ 0.02	$-$ 0.104
24	Higher education diploma	0.096	$-$ 0.092	0.168	$-$ 0.075	0.026	0.141	0.125	$-$ 0.14	0.023	0.22	0.173	$-$ 0.024	$-$ 0.034	0.157
25	Ethnicity other than Russian	$-$ 0.084	0.092	$-$ 0.022	0.121	$-$ 0.099	$-$ 0.042	$-$ 0.005	$-$ 0.047	$-$ 0.101	$-$ 0.108	$-$ 0.1	$-$ 0.111	$-$ 0.069	$-$ 0.195
26	Poverty	$-$ 0.003	0.069	$-$ 0.066	$-$ 0.088	0.099	0.007	0.021	0.169	0.064	$-$ 0.09	$-$ 0.078	0.019	0.202	$-$ 0.043
27	Alcohol cons. $>1$ time per week	0.064	0.012	0.017	0.012	$-$ 0.059	0.01	$-$ 0.006	$-$ 0.005	0.074	0.057	0.172	0.08	0.039	$-$ 0.008

Num variable		15	16	17	18	19	20	21	22	23	24	25	26	27	
1	Age	0.05	0.141	$-$ 0.049	$-$ 0.035	$-$ 0.046	0.038	0.083	$-$ 0.035	$-$ 0.096	0.096	$-$ 0.084	$-$ 0.003	0.064	
2	Sex (male)	$-$ 0.073	$-$ 0.018	0.053	$-$ 0.009	0.015	$-$ 0.054	$-$ 0.051	0.085	$-$ 0.001	$-$ 0.092	0.092	0.069	0.012	
3	Year	0.028	0.1	$-$ 0.062	$-$ 0.017	0.023	0.146	0.003	$-$ 0.056	$-$ 0.017	0.168	$-$ 0.022	$-$ 0.066	0.017	
4	In good/excellent health	$-$ 0.094	$-$ 0.011	0.074	0.009	0.011	$-$ 0.062	$-$ 0.053	$-$ 0.006	0.284	$-$ 0.075	0.121	$-$ 0.088	0.012	
5	Health score (1-highest, 5-lowest)	0.063	$-$ 0.04	$-$ 0.077	$-$ 0.015	$-$ 0.023	0.064	0.031	0.022	$-$ 0.295	0.026	$-$ 0.099	0.099	$-$ 0.059	
6	Health problem in last 30d	0.08	0.127	$-$ 0.006	0.032	0.009	0.058	0.111	0.009	$-$ 0.125	0.141	$-$ 0.042	0.007	0.01	
7	Num. of chronic conditions	0.173	0.142	0.047	$-$ 0.03	$-$ 0.002	0.047	0.119	$-$ 0.002	$-$ 0.153	0.125	$-$ 0.005	0.021	$-$ 0.006	
8	Bad/Satisfactory GPA	$-$ 0.163	$-$ 0.086	0.096	$-$ 0.066	$-$ 0.027	$-$ 0.086	0.023	0.076	0.021	$-$ 0.14	$-$ 0.047	0.169	$-$ 0.005	
9	School homework/assignments	0.156	0.141	0.001	0.007	0.013	0.111	$-$ 0.123	$-$ 0.058	$-$ 0.142	0.023	$-$ 0.101	0.064	0.074	
10	Extracurricular study	0.099	0.284	$-$ 0.083	0.073	0.058	0.142	0.184	$-$ 0.006	0.014	0.22	$-$ 0.108	$-$ 0.09	0.057	
11	Extracurricular arts	0.176	0.157	$-$ 0.046	0.115	0.012	0.18	0.122	0.007	0.004	0.173	$-$ 0.1	$-$ 0.078	0.172	
12	Extracurricular sports	$-$ 0.16	0.045	0.238	0.066	0.064	$-$ 0.064	$-$ 0.027	0.041	0.012	$-$ 0.024	$-$ 0.111	0.019	0.08	
13	Watching TV/on Internet	0.026	0.027	$-$ 0.079	0.04	0.03	0.078	0.152	0.041	$-$ 0.02	$-$ 0.034	$-$ 0.069	0.202	0.039	
14	Used a personal computer	0.207	0.136	$-$ 0.06	0.101	0.117	0.083	0.252	0.011	$-$ 0.104	0.157	$-$ 0.195	$-$ 0.043	$-$ 0.008	
15	Vacation with parent in 1yr	1	0.3	$-$ 0.139	0.022	0.07	0.174	0.235	$-$ 0.067	$-$ 0.077	0.299	$-$ 0.153	$-$ 0.047	$-$ 0.009	
16	Num. of excursions/exhibition visits in 1yr	0.3	1	$-$ 0.043	$-$ 0.008	$-$ 0.022	0.163	0.376	$-$ 0.044	$-$ 0.008	0.38	$-$ 0.153	$-$ 0.034	0.034	
17	Sees friends $>2$ times per week	$-$ 0.139	$-$ 0.043	1	0.019	0.007	$-$ 0.05	$-$ 0.111	0.037	0.071	$-$ 0.104	0.087	$-$ 0.04	0.022	
18	Received childcare in past 7 days	0.022	$-$ 0.008	0.019	1	0.747	$-$ 0.004	0.018	0.008	0.033	0.064	$-$ 0.077	$-$ 0.055	0.072	
19	Received childcare from a relative in past 7 days	0.07	$-$ 0.022	0.007	0.747	1	$-$ 0.077	0.037	$-$ 0.06	0.123	0.017	$-$ 0.082	$-$ 0.084	0.008	
20	Mother’s age	0.174	0.163	$-$ 0.05	$-$ 0.004	$-$ 0.077	1	0.172	$-$ 0.021	$-$ 0.123	0.229	$-$ 0.067	$-$ 0.105	$-$ 0.039	
21	Urban	0.235	0.376	$-$ 0.111	0.018	0.037	0.172	1	0.008	$-$ 0.072	0.21	$-$ 0.098	$-$ 0.021	0.069	
22	Single parent	$-$ 0.067	$-$ 0.044	0.037	0.008	$-$ 0.06	$-$ 0.021	0.008	1	$-$ 0.046	$-$ 0.077	$-$ 0.102	0.147	0.008	
23	In good/excellent health m	$-$ 0.077	$-$ 0.008	0.071	0.033	0.123	$-$ 0.123	$-$ 0.072	$-$ 0.046	1	$-$ 0.053	0.13	$-$ 0.147	$-$ 0.019	
24	Higher education diploma	0.299	0.38	$-$ 0.104	0.064	0.017	0.229	0.21	$-$ 0.077	$-$ 0.053	1	$-$ 0.174	$-$ 0.168	0.02	
25	Ethnicity other than Russian	$-$ 0.153	$-$ 0.153	0.087	$-$ 0.077	$-$ 0.082	$-$ 0.067	$-$ 0.098	$-$ 0.102	0.13	$-$ 0.174	1	$-$ 0.118	$-$ 0.075	
26	Poverty	$-$ 0.047	$-$ 0.034	$-$ 0.04	$-$ 0.055	$-$ 0.084	$-$ 0.105	$-$ 0.021	0.147	$-$ 0.147	$-$ 0.168	$-$ 0.118	1	$-$ 0.047	
27	Alcohol cons. $>1$ time per week	$-$ 0.009	0.034	0.022	0.072	0.008	$-$ 0.039	0.069	0.008	$-$ 0.019	0.02	$-$ 0.075	$-$ 0.047	1	

, 16

**Table 16:** Correlation matrix for main variables, whole sample (continued)

Num variable		28	29	30	31	32	33	34	35	36	37	38	39	40	41
28	Mothers subjective life satisfaction	1	0.241	0.034	0.161	$-$ 0.12	$-$ 0.05	$-$ 0.04	0.028	$-$ 0.04	0.041	0.098	0.195	0.031	$-$ 0.01
29	Household income in last 30 days	0.241	1	0.192	0.215	$-$ 0.07	$-$ 0.08	0.049	0.149	0.15	0.166	0.175	0.257	0.124	$-$ 0.11
30	Household savings in last 30 days	0.034	0.192	1	0.074	$-$ 0.04	$-$ 0.04	0.019	$-$ 0.04	$-$ 0.01	$-$ 0.01	$-$ 0.03	$-$ 0.02	0.034	0.029
31	Savings will last several months	0.161	0.215	0.074	1	$-$ 0.09	$-$ 0.13	$-$ 0	$-$ 0.01	0.029	0.025	0.106	0.053	0.054	0.001
32	Consumer loan in last 12 months	$-$ 0.12	$-$ 0.07	$-$ 0.04	$-$ 0.09	1	0.097	0.003	0.249	$-$ 0.03	0.015	0.014	0.06	$-$ 0.02	$-$ 0.04
33	Borrowed from a private individual in last 12 months	$-$ 0.05	$-$ 0.08	$-$ 0.04	$-$ 0.13	0.097	1	$-$ 0.01	0.043	$-$ 0.01	0.01	$-$ 0.03	$-$ 0.01	0.059	0.026
34	Loan to a private individual in last 12 months	$-$ 0.04	0.049	0.019	$-$ 0	0.003	$-$ 0.01	1	0.115	$-$ 0.01	0.161	$-$ 0.01	0.108	0.055	$-$ 0.04
35	Loan payments in last 30 days	0.028	0.149	$-$ 0.04	$-$ 0.01	0.249	0.043	0.115	1	0.009	0.026	0.103	0.111	0.041	$-$ 0.09
36	Vegetables/legumes	$-$ 0.04	0.15	$-$ 0.01	0.029	$-$ 0.03	$-$ 0.01	$-$ 0.01	0.009	1	0.101	0.171	0.198	0.021	0.007
37	Fruit (fresh and canned)	0.041	0.166	$-$ 0.01	0.025	0.015	0.01	0.161	0.026	0.101	1	0.182	0.26	0.051	$-$ 0.01
38	Meat and poultry	0.098	0.175	$-$ 0.03	0.106	0.014	$-$ 0.03	$-$ 0.01	0.103	0.171	0.182	1	0.384	0.338	0.024
39	Dairy	0.195	0.257	$-$ 0.02	0.053	0.06	$-$ 0.01	0.108	0.111	0.198	0.26	0.384	1	0.064	$-$ 0.09
40	Vodka and liquors (in litres)	0.031	0.124	0.034	0.054	$-$ 0.02	0.059	0.055	0.041	0.021	0.051	0.338	0.064	1	0.019
41	Refined sugar	$-$ 0.01	$-$ 0.11	0.029	0.001	$-$ 0.04	0.026	$-$ 0.04	$-$ 0.09	0.007	$-$ 0.01	0.024	$-$ 0.09	0.019	1
42	Candy and high-sugar treats	$-$ 0.04	0.049	0.001	0.026	0.037	0.102	0.178	0.029	0.029	0.214	0.12	0.201	0.005	0.048
43	Starches	0.005	$-$ 0.03	$-$ 0.07	$-$ 0.1	0.019	0.036	$-$ 0.04	$-$ 0.06	0.145	0.076	0.09	0.089	$-$ 0.02	0.406
44	Essential food items	0.16	0.317	0.007	0.163	0.01	$-$ 0.06	0.103	0.127	0.285	0.385	0.723	0.513	0.226	0.203
45	Clothes	0.093	0.358	0.062	0.078	$-$ 0.09	$-$ 0.06	0.115	$-$ 0	0.104	0.191	0.064	0.084	0.115	0.094
46	All basic expenditure	0.172	0.505	0.031	0.167	$-$ 0.04	$-$ 0.08	0.127	0.114	0.274	0.33	0.542	0.411	0.219	0.096
47	All non-essential expenditure	0.096	0.158	0.053	0.052	$-$ 0.06	$-$ 0.03	0.021	0.14	0.059	0.063	0.247	0.058	0.428	0.008
48	Purchase of durable goods	0.061	0.184	0.098	0.002	0.213	$-$ 0.02	0.097	0.124	0.061	0.109	0.115	0.195	0.184	$-$ 0.04
49	Utilities	0.102	0.356	0.01	0.09	$-$ 0.06	$-$ 0.04	0.032	0.064	0.136	0.016	0.22	0.178	0.057	$-$ 0.1
50	Essential services	0.045	0.319	$-$ 0.02	0.065	0.068	$-$ 0.03	0.049	0.117	0.125	0.136	0.166	0.137	0.126	$-$ 0.15
51	Household owns home	0.087	0.053	0.041	0.02	0.015	$-$ 0.03	0.05	0.122	$-$ 0.03	0.013	$-$ 0.03	$-$ 0.01	0.05	0.084
52	Hot water supply	0.08	0.272	0.013	0.07	0.087	$-$ 0.06	0.063	0.142	0.123	0.118	0.172	0.253	0.065	$-$ 0.26
53	Central sewerage	$-$ 0.06	0.251	0.007	$-$ 0.04	0.052	$-$ 0.02	0.053	0.164	0.109	0.137	0.202	0.297	0.046	$-$ 0.23
54	Central heating	$-$ 0.06	0.26	0.012	$-$ 0.04	0.086	$-$ 0.02	0.043	0.167	0.127	0.132	0.149	0.275	0.087	$-$ 0.26

Num variable		42	43	44	45	46	47	48	49	50	51	52	53	54	
28	Mothers subjective life satisfaction	$-$ 0.04	0.005	0.16	0.093	0.172	0.096	0.061	0.102	0.045	0.087	0.08	$-$ 0.06	$-$ 0.06	
29	Household income in last 30 days	0.049	$-$ 0.03	0.317	0.358	0.505	0.158	0.184	0.356	0.319	0.053	0.272	0.251	0.26	
30	Household savings in last 30 days	0.001	$-$ 0.07	0.007	0.062	0.031	0.053	0.098	0.01	$-$ 0.02	0.041	0.013	0.007	0.012	
31	Savings will last several months	0.026	$-$ 0.1	0.163	0.078	0.167	0.052	0.002	0.09	0.065	0.02	0.07	$-$ 0.04	$-$ 0.04	
32	Consumer loan in last 12 months	0.037	0.019	0.01	$-$ 0.09	$-$ 0.04	$-$ 0.06	0.213	$-$ 0.06	0.068	0.015	0.087	0.052	0.086	
33	Borrowed from a private individual in last 12 months	0.102	0.036	$-$ 0.06	$-$ 0.06	$-$ 0.08	$-$ 0.03	$-$ 0.02	$-$ 0.04	$-$ 0.03	$-$ 0.03	$-$ 0.06	$-$ 0.02	$-$ 0.02	
34	Loan to a private individual in last 12 months	0.178	$-$ 0.04	0.103	0.115	0.127	0.021	0.097	0.032	0.049	0.05	0.063	0.053	0.043	
35	Loan payments in last 30 days	0.029	$-$ 0.06	0.127	$-$ 0	0.114	0.14	0.124	0.064	0.117	0.122	0.142	0.164	0.167	
36	Vegetables/legumes	0.029	0.145	0.285	0.104	0.274	0.059	0.061	0.136	0.125	$-$ 0.03	0.123	0.109	0.127	
37	Fruit (fresh and canned)	0.214	0.076	0.385	0.191	0.33	0.063	0.109	0.016	0.136	0.013	0.118	0.137	0.132	
38	Meat and poultry	0.12	0.09	0.723	0.064	0.542	0.247	0.115	0.22	0.166	$-$ 0.03	0.172	0.202	0.149	
39	Dairy	0.201	0.089	0.513	0.084	0.411	0.058	0.195	0.178	0.137	$-$ 0.01	0.253	0.297	0.275	
40	Vodka and liquors (in litres)	0.005	$-$ 0.02	0.226	0.115	0.219	0.428	0.184	0.057	0.126	0.05	0.065	0.046	0.087	
41	Refined sugar	0.048	0.406	0.203	0.094	0.096	0.008	$-$ 0.04	$-$ 0.1	$-$ 0.15	0.084	$-$ 0.26	$-$ 0.23	$-$ 0.26	
42	Candy and high-sugar treats	1	0.215	0.372	0.159	0.273	0.01	0.106	$-$ 0.03	0.021	$-$ 0.02	$-$ 0.16	$-$ 0.08	$-$ 0.14	
43	Starches	0.215	1	0.362	0.092	0.23	$-$ 0.03	$-$ 0.02	$-$ 0.02	$-$ 0.06	0.059	$-$ 0.23	$-$ 0.22	$-$ 0.25	
44	Essential food items	0.372	0.362	1	0.238	0.801	0.234	0.182	0.263	0.236	0.02	0.144	0.173	0.11	
45	Clothes	0.159	0.092	0.238	1	0.624	0.184	0.236	0.099	0.184	0.089	$-$ 0.08	$-$ 0.05	$-$ 0.03	
46	All basic expenditure	0.273	0.23	0.801	0.624	1	0.257	0.232	0.58	0.5	$-$ 0.04	0.205	0.24	0.22	
47	All non-essential expenditure	0.01	$-$ 0.03	0.234	0.184	0.257	1	0.201	0.063	0.144	0.053	0.04	0.062	0.079	
48	Purchase of durable goods	0.106	$-$ 0.02	0.182	0.236	0.232	0.201	1	0.005	0.152	0.141	0.054	0.069	0.057	
49	Utilities	$-$ 0.03	$-$ 0.02	0.263	0.099	0.58	0.063	0.005	1	0.345	$-$ 0.29	0.368	0.355	0.38	
50	Essential services	0.021	$-$ 0.06	0.236	0.184	0.5	0.144	0.152	0.345	1	0.014	0.205	0.256	0.268	
51	Household owns home	$-$ 0.02	0.059	0.02	0.089	$-$ 0.04	0.053	0.141	$-$ 0.29	0.014	1	$-$ 0.04	$-$ 0.13	$-$ 0.16	
52	Hot water supply	$-$ 0.16	$-$ 0.23	0.144	$-$ 0.08	0.205	0.04	0.054	0.368	0.205	$-$ 0.04	1	0.651	0.705	
53	Central sewerage	$-$ 0.08	$-$ 0.22	0.173	$-$ 0.05	0.24	0.062	0.069	0.355	0.256	$-$ 0.13	0.651	1	0.82	
54	Central heating	$-$ 0.14	$-$ 0.25	0.11	$-$ 0.03	0.22	0.079	0.057	0.38	0.268	$-$ 0.16	0.705	0.82	1	

, 17

**Table 17:** Correlation matrix for main variables, whole sample (continued)

Num variable		1	2	3	4	5	6	7	8	9	10	11	12	13	14
28	Mothers subjective life satisfaction	0.066	$-$ 0.031	0.064	0.126	$-$ 0.17	$-$ 0.008	$-$ 0.066	$-$ 0.151	$-$ 0.016	0.066	0.078	0.056	0.017	$-$ 0.029
29	Household income in last 30 days	0.102	$-$ 0.158	0.087	$-$ 0.032	$-$ 0.002	0.02	0.055	$-$ 0.091	0.02	0.193	0.127	$-$ 0.036	0.143	0.118
30	Household savings in last 30 days	0.038	0.001	$-$ 0.014	0.034	$-$ 0.024	0.024	$-$ 0.001	$-$ 0.017	0.041	$-$ 0.027	$-$ 0.044	0.088	0.042	$-$ 0.007
31	Savings will last several months	$-$ 0.059	$-$ 0.095	$-$ 0.045	$-$ 0.092	0.037	$-$ 0.047	$-$ 0.047	$-$ 0.05	0.138	0.022	0.01	$-$ 0.071	$-$ 0.102	$-$ 0.068
32	Consumer loan in last 12 months	0.007	$-$ 0.11	$-$ 0.122	$-$ 0.136	0.147	0.09	0.135	$-$ 0.031	$-$ 0.046	$-$ 0.082	0.006	0.11	0.09	0.15
33	Borrowed from a private individual in last 12 months	0.027	0.085	0.016	$-$ 0.083	0.092	0.017	$-$ 0.03	0.163	$-$ 0.021	$-$ 0.049	$-$ 0.054	0.056	0.048	0.006
34	Loan to a private individual in last 12 months	0.064	0.099	$-$ 0.027	$-$ 0.074	0.068	0.184	0.111	$-$ 0.063	$-$ 0.025	0.091	$-$ 0.025	$-$ 0.01	0.045	0.113
35	Loan payments in last 30 days (in 2011 prices)	0.057	$-$ 0.089	0.039	$-$ 0.135	0.149	0.08	0.145	$-$ 0.079	$-$ 0.003	0.091	0.081	$-$ 0.061	0.106	0.112
36	Vegetables/legumes	0.011	$-$ 0.034	0.028	$-$ 0.086	0.039	0.075	0.04	0.002	0.055	0.083	0.024	0.091	$-$ 0.032	$-$ 0.023
37	Fruit (fresh and canned)	0	$-$ 0.042	$-$ 0.053	$-$ 0.022	0.009	$-$ 0.038	0.031	0.029	0.103	$-$ 0.003	$-$ 0.007	$-$ 0.025	$-$ 0.051	0.049
38	Meat and poultry	0.036	$-$ 0.039	0.016	$-$ 0.006	0.005	0.051	0.007	$-$ 0.039	0.105	$-$ 0.024	$-$ 0.012	$-$ 0.003	$-$ 0.034	0.061
39	Dairy	0.055	0.051	0.046	$-$ 0.123	0.06	0.055	0.172	$-$ 0.095	0.079	0.051	0.037	0.016	0.018	0.106
40	Vodka and liquors (in litres)	0.018	0.089	0.026	0.008	$-$ 0.009	$-$ 0.003	0.04	0.017	0.11	0.015	$-$ 0.012	0.026	0.114	0.077
41	Refined sugar	$-$ 0.028	$-$ 0.007	0.068	0.058	$-$ 0.036	$-$ 0.116	$-$ 0.084	$-$ 0.056	$-$ 0.045	$-$ 0.047	$-$ 0.131	0.036	$-$ 0.102	$-$ 0.155
42	Candy and high-sugar treats	0.052	0.049	0.076	$-$ 0.031	0.05	$-$ 0.005	0.039	0.067	0.015	$-$ 0.046	0.007	0.092	$-$ 0.053	0.071
43	Starches	$-$ 0.046	0.033	0.014	0.004	0.032	$-$ 0.05	$-$ 0.013	$-$ 0.014	$-$ 0.058	$-$ 0.04	$-$ 0.123	0.021	$-$ 0.045	$-$ 0.165
44	Essential food items	0.041	$-$ 0.073	0.045	$-$ 0.062	0.031	0.032	0.054	$-$ 0.117	0.121	0.039	$-$ 0.002	0.026	$-$ 0.053	0.069
45	Clothes	$-$ 0.024	$-$ 0.094	$-$ 0.108	$-$ 0.036	0.036	0.019	0.053	$-$ 0.082	0.01	0.066	0.046	0.051	0.001	0.032
46	All basic expenditure	0.029	$-$ 0.128	$-$ 0.016	$-$ 0.059	0.037	0.071	0.092	$-$ 0.151	0.064	0.103	0.058	0.035	0.049	0.134
47	All non-essential expenditure	$-$ 0.009	0.035	$-$ 0.037	0.045	$-$ 0.03	0.053	0.072	0	0.119	0.139	0.004	$-$ 0.027	0.108	0.111
48	Purchase of durable goods	0.021	$-$ 0.033	$-$ 0.072	$-$ 0.137	0.153	0.154	0.159	$-$ 0.028	0.085	0.053	0.075	0.051	0.143	0.189
49	Utilities	0.044	$-$ 0.093	0.019	0.006	$-$ 0.012	0.074	0.045	$-$ 0.107	$-$ 0.033	0.083	0.063	$-$ 0.019	0.167	0.15
50	Essential services	0.006	$-$ 0.089	$-$ 0.014	$-$ 0.058	0.048	0.12	0.137	$-$ 0.082	0.007	0.151	0.108	0.029	0.143	0.183
51	Household owns home	0.01	$-$ 0.057	0.01	$-$ 0.008	0.001	0.068	0.004	$-$ 0.068	0.032	0.031	$-$ 0.094	$-$ 0.055	0.044	0.021
52	Hot water supply	0.027	$-$ 0.103	$-$ 0.06	$-$ 0.012	$-$ 0.047	0.135	0.066	$-$ 0.026	$-$ 0.028	0.175	0.127	$-$ 0.021	0.13	0.238
53	Central sewerage	0.02	$-$ 0.08	$-$ 0.045	$-$ 0.035	0.003	0.056	0.083	$-$ 0.031	$-$ 0.049	0.175	0.125	$-$ 0.043	0.111	0.226
54	Central heating	0.001	$-$ 0.048	$-$ 0.068	$-$ 0.008	$-$ 0.014	0.095	0.065	0.047	$-$ 0.082	0.188	0.181	0.027	0.118	0.237

Num variable		15	16	17	18	19	20	21	22	23	24	25	26	27	28
28	Mothers subjective life satisfaction	0.102	0.149	0.075	$-$ 0.022	0.007	0.062	0.006	$-$ 0.231	0.221	0.184	0.11	$-$ 0.381	0.091	1
29	Household income in last 30 days	0.276	0.343	$-$ 0.087	$-$ 0.027	$-$ 0.075	0.199	0.298	$-$ 0.141	$-$ 0.11	0.378	$-$ 0.08	$-$ 0.1	0.246	0.241
30	Household savings in last 30 days	0.011	0.059	$-$ 0.044	$-$ 0.028	$-$ 0.01	0.037	0.064	$-$ 0.046	$-$ 0.059	$-$ 0.002	$-$ 0.042	0.065	0.137	0.034
31	Savings will last several months	0.05	0.169	$-$ 0.033	$-$ 0.015	$-$ 0.009	0.165	0.01	$-$ 0.096	0.011	0.118	0.039	$-$ 0.071	0.018	0.161
32	Consumer loan in last 12 months	0.01	$-$ 0.018	0.007	$-$ 0.029	$-$ 0.01	$-$ 0.036	0.09	0.079	$-$ 0.179	$-$ 0.067	$-$ 0.049	0.163	$-$ 0.079	$-$ 0.117
33	Borrowed from a private individual in last 12 months	$-$ 0.173	$-$ 0.089	0.042	0	0	0.002	$-$ 0.062	0.077	$-$ 0.065	$-$ 0.047	$-$ 0.027	0.027	0.053	$-$ 0.052
34	Loan to a private individual in last 12 months	0.038	0.176	$-$ 0.019	$-$ 0.028	$-$ 0.068	$-$ 0.02	0.073	$-$ 0.021	$-$ 0.023	0.113	0.024	0.038	$-$ 0.009	$-$ 0.042
35	Loan payments in last 30 days (in 2011 prices)	0.124	0.139	$-$ 0.123	$-$ 0.077	$-$ 0.027	0.063	0.148	$-$ 0.117	$-$ 0.039	0.179	$-$ 0.071	0.063	$-$ 0.009	0.028
36	Vegetables/legumes	0.086	0.157	$-$ 0.062	0	$-$ 0.079	0.038	0.161	$-$ 0.01	$-$ 0.09	0.143	$-$ 0.111	0.03	0.031	$-$ 0.035
37	Fruit (fresh and canned)	0.118	0.15	0.021	0.005	$-$ 0.035	$-$ 0.019	0.116	0.023	$-$ 0.037	0.097	$-$ 0.076	$-$ 0.023	$-$ 0.049	0.041
38	Meat and poultry	0.228	0.201	$-$ 0.046	0.027	0.019	0.074	0.104	$-$ 0.053	0.031	0.098	$-$ 0.02	$-$ 0.03	$-$ 0.015	0.098
39	Dairy	0.222	0.31	$-$ 0.042	$-$ 0.072	$-$ 0.066	0.054	0.28	$-$ 0.117	$-$ 0.046	0.168	$-$ 0.02	$-$ 0.018	$-$ 0.044	0.195
40	Vodka and liquors (in litres)	0.062	0.135	$-$ 0.055	$-$ 0.051	$-$ 0.034	0.054	0.047	$-$ 0.047	0.029	0.054	$-$ 0.063	$-$ 0.002	0.056	0.031
41	Refined sugar	$-$ 0.245	$-$ 0.167	0.059	$-$ 0.148	$-$ 0.084	$-$ 0.05	$-$ 0.232	$-$ 0.059	0.12	$-$ 0.143	0.281	$-$ 0.069	$-$ 0.022	$-$ 0.005
42	Candy and high-sugar treats	$-$ 0.056	0.009	0.046	$-$ 0.002	$-$ 0.048	$-$ 0.05	$-$ 0.165	0.029	$-$ 0.025	$-$ 0.013	$-$ 0.05	0.003	$-$ 0.002	$-$ 0.041
43	Starches	$-$ 0.141	$-$ 0.166	0.038	$-$ 0.133	$-$ 0.159	$-$ 0.072	$-$ 0.221	0.028	0.06	$-$ 0.158	0.226	$-$ 0.025	$-$ 0.036	0.005
44	Essential food items	0.197	0.257	$-$ 0.087	$-$ 0.052	$-$ 0.072	0.061	0.086	$-$ 0.023	0.047	0.137	0.013	$-$ 0.067	0.014	0.16
45	Clothes	$-$ 0.011	0.19	$-$ 0.021	0.012	$-$ 0.021	$-$ 0.056	$-$ 0.028	$-$ 0.06	$-$ 0.019	0.127	0.021	$-$ 0.121	0.038	0.093
46	All basic expenditure	0.219	0.351	$-$ 0.128	$-$ 0.023	$-$ 0.053	0.107	0.209	$-$ 0.014	0.005	0.218	0.014	$-$ 0.091	0.036	0.172
47	All non-essential expenditure	0.056	0.136	$-$ 0.008	0.031	0.062	0.027	0.011	$-$ 0.068	0.056	0.023	$-$ 0.019	0.025	0.041	0.096
48	Purchase of durable goods	0.085	0.173	0.059	0.061	0.047	0.009	0.1	$-$ 0.039	$-$ 0.134	0.123	$-$ 0.102	0.014	0.008	0.061
49	Utilities	0.218	0.218	$-$ 0.146	$-$ 0.03	$-$ 0.02	0.195	0.36	0.106	$-$ 0.016	0.133	0.014	0.007	0.01	0.102
50	Essential services	0.228	0.297	$-$ 0.121	0.068	0.023	0.182	0.308	$-$ 0.055	$-$ 0.05	0.244	$-$ 0.042	$-$ 0.016	0.05	0.045
51	Household owns home	0.041	0.047	$-$ 0.031	$-$ 0.116	$-$ 0.102	0.088	$-$ 0.032	$-$ 0.245	0.015	0.071	$-$ 0.123	$-$ 0.186	$-$ 0.043	0.087
52	Hot water supply	0.342	0.301	$-$ 0.113	$-$ 0.017	$-$ 0.025	0.204	0.562	$-$ 0.025	$-$ 0.083	0.211	$-$ 0.145	0.057	0.102	0.08
53	Central sewerage	0.275	0.316	$-$ 0.079	0.06	0.016	0.163	0.616	0.117	$-$ 0.113	0.197	$-$ 0.136	0.055	0.079	$-$ 0.059
54	Central heating	0.22	0.35	$-$ 0.084	0.019	0.006	0.135	0.631	0.162	$-$ 0.05	0.242	$-$ 0.158	0.062	0.092	$-$ 0.056

and 18.

**Table 18:** Correlation matrix for main variables, whole sample (continued)

Num variable		27	28	29	30	31	32	33	34	35	36	37	38	39	40
1	Age	0.064	0.066	0.102	0.038	$-$ 0.059	0.007	0.027	0.064	0.057	0.011	0	0.036	0.055	0.018
2	Sex (male)	0.012	$-$ 0.031	$-$ 0.158	0.001	$-$ 0.095	$-$ 0.11	0.085	0.099	$-$ 0.089	$-$ 0.034	$-$ 0.042	$-$ 0.039	0.051	0.089
3	Year	0.017	0.064	0.087	$-$ 0.014	$-$ 0.045	$-$ 0.122	0.016	$-$ 0.027	0.039	0.028	$-$ 0.053	0.016	0.046	0.026
4	In good/excellent health	0.012	0.126	$-$ 0.032	0.034	$-$ 0.092	$-$ 0.136	$-$ 0.083	$-$ 0.074	$-$ 0.135	$-$ 0.086	$-$ 0.022	$-$ 0.006	$-$ 0.123	0.008
5	Health score (1-highest, 5-lowest)	$-$ 0.059	$-$ 0.17	$-$ 0.002	$-$ 0.024	0.037	0.147	0.092	0.068	0.149	0.039	0.009	0.005	0.06	$-$ 0.009
6	Health problem in last 30d	0.01	$-$ 0.008	0.02	0.024	$-$ 0.047	0.09	0.017	0.184	0.08	0.075	$-$ 0.038	0.051	0.055	$-$ 0.003
7	Num. of chronic conditions	$-$ 0.006	$-$ 0.066	0.055	$-$ 0.001	$-$ 0.047	0.135	$-$ 0.03	0.111	0.145	0.04	0.031	0.007	0.172	0.04
8	Bad/Satisfactory GPA	$-$ 0.005	$-$ 0.151	$-$ 0.091	$-$ 0.017	$-$ 0.05	$-$ 0.031	0.163	$-$ 0.063	$-$ 0.079	0.002	0.029	$-$ 0.039	$-$ 0.095	0.017
9	School homework/assignments	0.074	$-$ 0.016	0.02	0.041	0.138	$-$ 0.046	$-$ 0.021	$-$ 0.025	$-$ 0.003	0.055	0.103	0.105	0.079	0.11
10	Extracurricular study	0.057	0.066	0.193	$-$ 0.027	0.022	$-$ 0.082	$-$ 0.049	0.091	0.091	0.083	$-$ 0.003	$-$ 0.024	0.051	0.015
11	Extracurricular arts	0.172	0.078	0.127	$-$ 0.044	0.01	0.006	$-$ 0.054	$-$ 0.025	0.081	0.024	$-$ 0.007	$-$ 0.012	0.037	$-$ 0.012
12	Extracurricular sports	0.08	0.056	$-$ 0.036	0.088	$-$ 0.071	0.11	0.056	$-$ 0.01	$-$ 0.061	0.091	$-$ 0.025	$-$ 0.003	0.016	0.026
13	Watching TV/on Internet	0.039	0.017	0.143	0.042	$-$ 0.102	0.09	0.048	0.045	0.106	$-$ 0.032	$-$ 0.051	$-$ 0.034	0.018	0.114
14	Used a personal computer	$-$ 0.008	$-$ 0.029	0.118	$-$ 0.007	$-$ 0.068	0.15	0.006	0.113	0.112	$-$ 0.023	0.049	0.061	0.106	0.077
15	Vacation with parent in 1yr	$-$ 0.009	0.102	0.276	0.011	0.05	0.01	$-$ 0.173	0.038	0.124	0.086	0.118	0.228	0.222	0.062
16	Num. of excursions/exhibitions in 1yr	0.034	0.149	0.343	0.059	0.169	$-$ 0.018	$-$ 0.089	0.176	0.139	0.157	0.15	0.201	0.31	0.135
17	Sees friends $>2$ times per week	0.022	0.075	$-$ 0.087	$-$ 0.044	$-$ 0.033	0.007	0.042	$-$ 0.019	$-$ 0.123	$-$ 0.062	0.021	$-$ 0.046	$-$ 0.042	$-$ 0.055
18	Childcare in past 7 days	0.072	$-$ 0.022	$-$ 0.027	$-$ 0.028	$-$ 0.015	$-$ 0.029	0	$-$ 0.028	$-$ 0.077	0	0.005	0.027	$-$ 0.072	$-$ 0.051
19	Childcare from a relative in past 7 days	0.008	0.007	$-$ 0.075	$-$ 0.01	$-$ 0.009	$-$ 0.01	0	$-$ 0.068	$-$ 0.027	$-$ 0.079	$-$ 0.035	0.019	$-$ 0.066	$-$ 0.034
20	Mother’s age	$-$ 0.039	0.062	0.199	0.037	0.165	$-$ 0.036	0.002	$-$ 0.02	0.063	0.038	$-$ 0.019	0.074	0.054	0.054
21	Urban	0.069	0.006	0.298	0.064	0.01	0.09	$-$ 0.062	0.073	0.148	0.161	0.116	0.104	0.28	0.047
22	Single parent	0.008	$-$ 0.231	$-$ 0.141	$-$ 0.046	$-$ 0.096	0.079	0.077	$-$ 0.021	$-$ 0.117	$-$ 0.01	0.023	$-$ 0.053	$-$ 0.117	$-$ 0.047
23	In good/excellent health m	$-$ 0.019	0.221	$-$ 0.11	$-$ 0.059	0.011	$-$ 0.179	$-$ 0.065	$-$ 0.023	$-$ 0.039	$-$ 0.09	$-$ 0.037	0.031	$-$ 0.046	0.029
24	Higher education diploma	0.02	0.184	0.378	$-$ 0.002	0.118	$-$ 0.067	$-$ 0.047	0.113	0.179	0.143	0.097	0.098	0.168	0.054
25	Ethnicity other than Russian	$-$ 0.075	0.11	$-$ 0.08	$-$ 0.042	0.039	$-$ 0.049	$-$ 0.027	0.024	$-$ 0.071	$-$ 0.111	$-$ 0.076	$-$ 0.02	$-$ 0.02	$-$ 0.063
26	Poverty	$-$ 0.047	$-$ 0.381	$-$ 0.1	0.065	$-$ 0.071	0.163	0.027	0.038	0.063	0.03	$-$ 0.023	$-$ 0.03	$-$ 0.018	$-$ 0.002
27	Alcohol cons. $>1$ time per week	1	0.091	0.246	0.137	0.018	$-$ 0.079	0.053	$-$ 0.009	$-$ 0.009	0.031	$-$ 0.049	$-$ 0.015	$-$ 0.044	0.056

Num variable		41	42	43	44	45	46	47	48	49	50	51	52	53	54
1	Age	$-$ 0.028	0.052	$-$ 0.046	0.041	$-$ 0.024	0.029	$-$ 0.009	0.021	0.044	0.006	0.01	0.027	0.02	0.001
2	Sex (male)	$-$ 0.007	0.049	0.033	$-$ 0.073	$-$ 0.094	$-$ 0.128	0.035	$-$ 0.033	$-$ 0.093	$-$ 0.089	$-$ 0.057	$-$ 0.103	$-$ 0.08	$-$ 0.048
3	Year	0.068	0.076	0.014	0.045	$-$ 0.108	$-$ 0.016	$-$ 0.037	$-$ 0.072	0.019	$-$ 0.014	0.01	$-$ 0.06	$-$ 0.045	$-$ 0.068
4	In good/excellent health	0.058	$-$ 0.031	0.004	$-$ 0.062	$-$ 0.036	$-$ 0.059	0.045	$-$ 0.137	0.006	$-$ 0.058	$-$ 0.008	$-$ 0.012	$-$ 0.035	$-$ 0.008
5	Health score (1-highest, 5-lowest)	$-$ 0.036	0.05	0.032	0.031	0.036	0.037	$-$ 0.03	0.153	$-$ 0.012	0.048	0.001	$-$ 0.047	0.003	$-$ 0.014
6	Health problem in last 30d	$-$ 0.116	$-$ 0.005	$-$ 0.05	0.032	0.019	0.071	0.053	0.154	0.074	0.12	0.068	0.135	0.056	0.095
7	Num. of chronic conditions	$-$ 0.084	0.039	$-$ 0.013	0.054	0.053	0.092	0.072	0.159	0.045	0.137	0.004	0.066	0.083	0.065
8	Bad/Satisfactory GPA	$-$ 0.056	0.067	$-$ 0.014	$-$ 0.117	$-$ 0.082	$-$ 0.151	0	$-$ 0.028	$-$ 0.107	$-$ 0.082	$-$ 0.068	$-$ 0.026	$-$ 0.031	0.047
9	School homework/assignments	$-$ 0.045	0.015	$-$ 0.058	0.121	0.01	0.064	0.119	0.085	$-$ 0.033	0.007	0.032	$-$ 0.028	$-$ 0.049	$-$ 0.082
10	Extracurricular study	$-$ 0.047	$-$ 0.046	$-$ 0.04	0.039	0.066	0.103	0.139	0.053	0.083	0.151	0.031	0.175	0.175	0.188
11	Extracurricular arts	$-$ 0.131	0.007	$-$ 0.123	$-$ 0.002	0.046	0.058	0.004	0.075	0.063	0.108	$-$ 0.094	0.127	0.125	0.181
12	Extracurricular sports	0.036	0.092	0.021	0.026	0.051	0.035	$-$ 0.027	0.051	$-$ 0.019	0.029	$-$ 0.055	$-$ 0.021	$-$ 0.043	0.027
13	Watching TV/on Internet	$-$ 0.102	$-$ 0.053	$-$ 0.045	$-$ 0.053	0.001	0.049	0.108	0.143	0.167	0.143	0.044	0.13	0.111	0.118
14	Used a personal computer	$-$ 0.155	0.071	$-$ 0.165	0.069	0.032	0.134	0.111	0.189	0.15	0.183	0.021	0.238	0.226	0.237
15	Vacation with parent in 1yr	$-$ 0.245	$-$ 0.056	$-$ 0.141	0.197	$-$ 0.011	0.219	0.056	0.085	0.218	0.228	0.041	0.342	0.275	0.22
16	Num. of excursions/exhibitions in 1yr	$-$ 0.167	0.009	$-$ 0.166	0.257	0.19	0.351	0.136	0.173	0.218	0.297	0.047	0.301	0.316	0.35
17	Sees friends $>2$ times per week	0.059	0.046	0.038	$-$ 0.087	$-$ 0.021	$-$ 0.128	$-$ 0.008	0.059	$-$ 0.146	$-$ 0.121	$-$ 0.031	$-$ 0.113	$-$ 0.079	$-$ 0.084
18	Childcare in past 7 days	$-$ 0.148	$-$ 0.002	$-$ 0.133	$-$ 0.052	0.012	$-$ 0.023	0.031	0.061	$-$ 0.03	0.068	$-$ 0.116	$-$ 0.017	0.06	0.019
19	Childcare from a relative in past 7 days	$-$ 0.084	$-$ 0.048	$-$ 0.159	$-$ 0.072	$-$ 0.021	$-$ 0.053	0.062	0.047	$-$ 0.02	0.023	$-$ 0.102	$-$ 0.025	0.016	0.006
20	Mother’s age	$-$ 0.05	$-$ 0.05	$-$ 0.072	0.061	$-$ 0.056	0.107	0.027	0.009	0.195	0.182	0.088	0.204	0.163	0.135
21	Urban	$-$ 0.232	$-$ 0.165	$-$ 0.221	0.086	$-$ 0.028	0.209	0.011	0.1	0.36	0.308	$-$ 0.032	0.562	0.616	0.631
22	Single parent	$-$ 0.059	0.029	0.028	$-$ 0.023	$-$ 0.06	$-$ 0.014	$-$ 0.068	$-$ 0.039	0.106	$-$ 0.055	$-$ 0.245	$-$ 0.025	0.117	0.162
23	In good/excellent health m	0.12	$-$ 0.025	0.06	0.047	$-$ 0.019	0.005	0.056	$-$ 0.134	$-$ 0.016	$-$ 0.05	0.015	$-$ 0.083	$-$ 0.113	$-$ 0.05
24	Higher education diploma	$-$ 0.143	$-$ 0.013	$-$ 0.158	0.137	0.127	0.218	0.023	0.123	0.133	0.244	0.071	0.211	0.197	0.242
25	Ethnicity other than Russian	0.281	$-$ 0.05	0.226	0.013	0.021	0.014	$-$ 0.019	$-$ 0.102	0.014	$-$ 0.042	$-$ 0.123	$-$ 0.145	$-$ 0.136	$-$ 0.158
26	Poverty	$-$ 0.069	0.003	$-$ 0.025	$-$ 0.067	$-$ 0.121	$-$ 0.091	0.025	0.014	0.007	$-$ 0.016	$-$ 0.186	0.057	0.055	0.062
27	Alcohol cons. $>1$ time per week	$-$ 0.022	$-$ 0.002	$-$ 0.036	0.014	0.038	0.036	0.041	0.008	0.01	0.05	$-$ 0.043	0.102	0.079	0.092

## Appendix 3

### Lowess Specifications

As mentioned in Sect. 4, although RDD models can produce unbiased treatment effect estimates due to near-complete randomisation around the cut-off threshold, this property depends on the correctness of the functional form of the trend before and after the intervention. While polynomials specifications with to the 3rd degree and a jump at the cut-off value are a very widespread approximation used in applied research, it is generally recommended to apply semi- and non-parametric methods to provide additional evidence supporting the robustness of obtained results.

It is worthwhile to note that the functional flexibility of Lowess comes at a cost. In the context of this study, the most important disadvantage lies in the lack of robust methods for deriving errors and confidence intervals. Neither window overlap (used in this study) nor bootstrap—the two popular derivation methods – can produce consistent standard errors without making restrictive model assumptions. Secondly, these methods cannot completely overcome the issue of choosing a functional form since point estimates in non-parametric models (such as Lowess and kernel regression) also rely on implicit assumptions relating, for example, to the degree of curve smoothing. Finally, this moderate advantage of increased flexibility also comes at the cost of having to deal with the curse of dimensionality. It manifests itself in a process whereby as the model complexity grows, for an increasingly high share of data/prediction points it becomes impossible to find sufficiently close matches among available observations for most kernel weighing functions. As a result, it severely restricts the number of covariates that can be included in models. Thus, it is generally recommended to view semi- and non-parametric estimation as a complement rather than a substitute for functional RDD. *(Lee and Lemieux, 2010)*

In this Appendix, I test local linear regression models (Lowess) of the form $y_{i} = f(birthtimeline_{i} + post2007_{i}) + \epsilon _{i}$ wherein, as explanatory variables, I include the birth timeline centred at January 2007 and having the month as the measurement unit, and the dummy *post*2007 for births in or after January 2007. Observation weights, provided by a tricubic kernel function, are computed with and without taking *post*2007 variable into account (i.e. “2d kernel" and “1d kernel" fit).

Non-parametric Lowess estimation results for the MC eligibility effect on various child and household outcomes are presented in Table 19 in Appendix 3. Overall, although the results are vastly statistically insignificant, the effect signs and sizes mirror the main findings of the parametric RDD. In particular, Lowess estimates validate a 0.1-0.2 reduction in the number of reported child chronic health conditions, which might be linked to improved housing conditions (i.e. a higher estimated prevalence of housing units with central heating and central sewerage in MC-eligible families). On the other hand, Lowess models also confirm the moderately negative impact of MC on some socialisation metrics. Of particular concern is an estimated 20-25 p.p. reduction in the share of children who reportedly saw their friends more than two times per week. Social isolation and loneliness are known to be significant risk factors for developing depression and anxiety both during periods of reduced social contact and afterwards (Loades et al., 2020).

As far as household financial behaviour is concerned, Lowess models render similar estimation results with regard to the observed increase in the average volume of monthly loan payments (2-3.5K Rub/65-120 USD), a higher share of MC-eligible households who borrow from private individuals, a reduced share of lending households, as well as a lower share of households with significant savings.

Lowess estimation depicts a similar consumption profile of MC-eligible families. In particular, MC-eligible families are found to spend more resources on essential food items (1.2-3K Rub/40-100 USD), utilities (0.7-1.3K Rub/25-40 USD), and less on clothing (1-1.5K/30-50 USD). The estimates for non-essential items and household savings made in the last 30 days tend to be of unstable magnitude, however. Finally, consistently negative coefficients for the consumption of strong liquors in models with different time windows and specifications provide tentative evidence for a decreased consumption of strong liquors by around 0.1-0.2 litres per week

**Table 19:** Lowess (1d and 2d kernel) regression discontinuity estimates of MC impact on the 2nd child outcomes and household consumption and diet (12, 24 and 36 months’ window at 1 January 2007 cut-off birth date)

Variable	RDD 12m 2d kernel	RDD 12m 1d kernel	RDD 24m 2d kernel	RDD 24m 1d kernel	RDD 36m 2d kernel	RDD 36m 1d kernel	Relative effect (%)
	(1)	(2)	(3)	(4)	(5)	(6)	
*Child health outcomes:*							
In good/excellent health	$-$ 0.073	$-$ 0.07	0.064	0.072	0.067	0.09	9.9
Health score (1-highest, 5$-$ lowest)	0.022	0.052	$-$ 0.101	$-$ 0.108	$-$ 0.109	$-$ 0.12	1.6
Num. of chronic conditions	$-$ 0.095	$-$ 0.071	$-$ 0.155	$-$ 0.206	$-$ 0.093	$-$ 0.195	$-$ 21
Health problem in last 30d	$-$ 0.021	0.085	0.003	0.025	$-$ 0.024	0.024	9.8
*Time spending:*							
Extracurricular study (in mins per week)	7.981	$-$ 12.305	13.877	6.846	11.719	11.753	$-$ 19.6
Extracurricular arts (in mins per week)	84.869	27.494	$-$ 11.048	12.64	$-$ 24.843	$-$ 15.433	40.8
Extracurricular sports (in mins per week)	105.53	$-$ 7.616	$-$ 52.826	79.271	$-$ 66.214	$-$ 26.644	17.9
Total productive time (in mins per week)	198.429	9.96	$-$ 52.388	97.224	$-$ 80.511	$-$ 32.377	24.7
Watching TV/on Internet (in mins per week)	42.674	45.108	8.656	20.005	9.313	10.976	37.7
Used a personal computer	0.275	0.289	0.121	0.21	0.084	0.141	41.5
*Well-being outcomes:*							
Vacation with parent in 1yr	0.185	0.19	$-$ 0.005	0.035	$-$ 0.115	0.037	25.6
Num. of excursions/exhibition visits in 1yr	$-$ 2.793	$-$ 3.356	$-$ 1.578	$-$ 2.497*	$-$ 1.145	$-$ 2.084*	$-$ 63.3
Sees friends $>2$ times per week	$-$ 0.245	$-$ 0.253	$-$ 0.23*	$-$ 0.174	$-$ 0.17	$-$ 0.223**	$-$ 37.5
Received childcare in past 7 days	0.066	$-$ 0.064	$-$ 0.078	$-$ 0.051	$-$ 0.082	$-$ 0.087	0.3
Received childcare from a relative in past 7 days	$-$ 0.003	$-$ 0.089	$-$ 0.088	$-$ 0.118	$-$ 0.093	$-$ 0.121	$-$ 24.2
Mothers subjective life satisfaction (1$-$ lowest, 5$-$ highest)	0.3	0	0.14	0.189	$-$ 0.045	0.225	4.4
*Household diet:*							
Vegetables/legumes (in kg)	$-$ 1.159	$-$ 1.94	$-$ 0.179	0.092	0.192	0.008	$-$ 68.1
Fruit (fresh and canned) (in kg)	$-$ 1.026	$-$ 0.053	$-$ 0.056	$-$ 0.536	$-$ 0.008	$-$ 0.193	$-$ 14.7
Meat and poultry (in kg)	$-$ 0.261	$-$ 1.238	0.047	$-$ 0.31	0.014	$-$ 0.011	$-$ 25.5
Dairy (in kg)	2.052	2.144	0.344	1.415	$-$ 0.132	0.804	34.2
Vodka and liquors (in litres)	$-$ 0.189	$-$ 0.084	$-$ 0.043	$-$ 0.093	$-$ 0.085	$-$ 0.051	$-$ 77.2
Refined sugar (in kg)	0.399	0.751	0.105	0.604	$-$ 0.11	0.392	44.8
Candy and high-sugar treats (in kg)	$-$ 0.016	0.246	0.179	0.127	0.054	0.247	7.4
Starches (in kg)	$-$ 1.258	2.743	1.638	0.824	2.115	1.302	8.3
*Household spending, by category:*							
Essential food items (in Rub)	2.094	3.59	1.785	2.121	1.272	2.331*	31.7
Clothes (in Rub)	$-$ 1.516	$-$ 0.786	$-$ 1.268	$-$ 1.699	$-$ 1.224	$-$ 1.533	$-$ 23.4
Utilities (in Rub)	0.703	0.704	1.079	1.265	0.807	1.374*	22.2
Essential services (in Rub)	$-$ 0.182	$-$ 1.167	0.09	$-$ 0.251	$-$ 0.032	$-$ 0.101	$-$ 27.4
All basic expenditure (in Rub)	1.099	2.341	1.686	1.436	0.823	2.071	8.8
All non-essential expenditure (in Rub)	2.818	3.122	1.462	0.076	0.277	0.631	61.8
Purchase of durable goods (in Rub)	0.101	0.306	$-$ 0.042	$-$ 0.025	$-$ 0.086	$-$ 0.036	49
*Saving, borrowing, and lending:*							
Household savings in last 30 days (in 2011 prices)	0.139	$-$ 0.677	0.142	$-$ 0.435	$-$ 0.289	$-$ 0.162	$-$ 15.3
Savings will last several months	$-$ 0.019	0.104	$-$ 0.035	$-$ 0.114	$-$ 0.034	$-$ 0.074	19.8
Loan payments in last 30 days (in 2011 prices)	3.204	3.227	2.55	3.084*	2.169	2.911*	84.2
Consumer loan in last 12 months	0.118	0.185	0.019	0.044	0.026	0.034	89.1
Borrowed from a private individual in last 12 months	0.154	0.068	0.113	0.129*	0.114*	0.119*	181.3
Loan to a private individual in last 12 months	$-$ 0.131	$-$ 0.067	$-$ 0.044	$-$ 0.067	$-$ 0.026	$-$ 0.05	$-$ 90.3
*Housing characteristics:*							
Household owns home	$-$ 0.048	$-$ 0.048	0.069	$-$ 0.043	0.032	0.028	$-$ 5.7
Hot water supply	0.136	0.216	0.015	0.076	$-$ 0.009	0.06	30.1
Central sewerage	0.193	0.208	0.044	0.157	$-$ 0.037	0.124	31.7
Central heating	0.151	0.118	0.063	0.173	0.027	0.138	23
Num. of observations	328	328	727	727	1047	1047	

***− 1 % sign., **− 5% sign., *− 10% sign

## 3.2 Additional Regression Tables

See Tables 20

**Table 20:** Regression discontinuity models on 2nd child health outcomes (36 months’ window at 1 January 2007 cut-off birth date)

	Health score (1-excellent 5-bad)		Good/excellent health (self-reported)		N. of chronic cond.		Health problem in 30d	
	RDD12m	RDD36m	RDD12m	RDD36m	RDD12m	RDD36m	RDD12m	RDD36m
*Post*	$-$ 0.004	$-$ 0.047	$-$ 0.032	0.024	$-$ 0.124	$-$ 0.336	$-$ 0.002	0.001
	(0.113)	(0.149)	(0.103)	(0.117)	(0.135)	(0.167)**	(0.113)	(0.138)
Child’s sex (male)		$-$ 0.01		0		0.048		0.027
		(0.032)		(0.025)		(0.039)		(0.029)
*Mother’s characteristics*								
Age		0.007		$-$ 0.002		0.001		$-$ 0.001
		(0.04)		(0.002)		(0.004)		(0.003)
Higher education		$-$ 0.018		$-$ 0.038		0.034		0.045
		(0.0365)		(0.036)		(0.045)		(0.034)
Ethnicity other than Russian		$-$ 0.084		0.042		0.043		$-$ 0.059
		(0.048)*		(0.039)		(0.062)		(0.046)
In good excellent health		$-$ 0.305		0.256		$-$ 0.197		$-$ 0.103
		(0.031)***		(0.025)***		( 0.04)***		(0.03)***
*Household characteristics*								
Single parent		0.067		$-$ 0.045		0.062		$-$ 0.006
		(0.044)		(0.036)		(0.057)**		(0.043)
Poverty		0.061		$-$ 0.063		0.045		0.04
		(0.032)*		(0.026)**		(0.023)		(0.031)
Urban		0.051		$-$ 0.042		0.178		0.0125
		(0.033)		(0.027)		(0.043***		(0.032)
Intercept	2.273	$-$ 8.822	0.714	10.277	0.468	$-$ 2.21	0.372	$-$ 3.323
	(0.087)***	(13.724)	(0.08)***	(10.72)	(0.127)***	(16.84)	(0.088)***	(12.731)
*Other controls*	NO	YES	NO	YES	NO	YES	NO	YES
*Linear trend*	YES	YES	YES	YES	YES	YES	YES	YES
*Quadratic trend*	NO	YES	NO	YES	NO	YES	NO	YES
*Cubic trend*	NO	YES	NO	NO	YES	NO	YES	NO
*RLMS year controls*	YES	YES	YES	YES	YES	YES	YES	YES
N. observations	328	1047	328	1047	328	1047	328	1047
R-squared	0.015	0.037	0.013	0.133	0.018	0.065	0.01	0.046
R-squared adjusted	$-$ 0.003	0.016	$-$ 0.005	0.11	0.000	0.041	$-$ 0.008	0.022

***− 1 % sign., **− 5% sign., *− 10% sign. Coefficient std. errors s are given in parentheses under the coefficient. Error terms are clustered at regional level

, 21

**Table 21:** Regression discontinuity estimates of impact of MC on 2nd child educational and time spending outcome (12, 24 and 36 months’ window at 1 January 2007 cut-off birth date)

Variable	RDD 12m	RDD 12m	RDD 24m	RDD 24m	RDD 36m	RDD 36m	Relative effect size (%)
	(1)	(2)	(3)	(4)	(5)	(6)	
In good/excellent health	$-$ 0.114	0.036	$-$ 0.012	0.076	$-$ 0.072	0.052	$-$ 5.30
	(0.128)	(0.121)	(0.136)	(0.125)	(0.143)	(0.139)	
Health score (1-highest, 5$-$ lowest)	0.11	$-$ 0.033	$-$ 0.026	$-$ 0.112	0.093	$-$ 0.02	1.70
	(0.13)	(0.122)	(0.146)	(0.136)	(0.154)	(0.153)	
Num. of chronic conditions	$-$ 0.056	$-$ 0.164	$-$ 0.176	$-$ 0.261	$-$ 0.04	$-$ 0.154	$-$ 25.10
	(0.208)	(0.232)	(0.215)	(0.223)	(0.225)	(0.232)	
Health problem in last 30d	0.037	$-$ 0.012	$-$ 0.029	$-$ 0.107	0.063	0.007	3.70
	(0.164)	(0.162)	(0.178)	(0.169)	(0.185)	(0.179)	
School homework/assignments (in mins per week)	$-$ 37.411	$-$ 39.969	$-$ 74.437	$-$ 75.957	$-$ 29.748	$-$ 38.63	$-$ 19.80
	(77.298)	(84.377)	(77.37)	(79.278)	(80.658)	(78.951)	
Extracurricular study (in mins per week)	20.003	28.084	5.872	9.85	21.444	18.231	145.40
	(16.029)	(20.526)	(12.824)	(13.923)	(17.928)	(18.778)	
Extracurricular arts (in mins per week)	68.93	79.43	26.187	40.437	36.5	67.752	56.80
	(60.264)	(70.031)	(67.284)	(66.822)	(66.115)	(66.226)	
Watching TV/on Internet (in mins per week)	16.098	1.9	25.157	14.42	41.916	30.41	7.70
	(28.098)	(30.058)	(28.877)	(29.309)	(28.369)	(29.118)	
Extracurricular sports (in mins per week)	105.323	86.548	22.823	$-$ 2.557	33.033	40.664	31.90
	(146.318)	(126.887)	(145.95)	(142.597)	(151.11)	(147.974)	
Productive time (in mins per week)	180.317	192.396	37.873	38.16	78.251	118.912	41.60
	(149.671)	(140.828)	(155.893)	(163.229)	(166.031)	(167.612)	
Vacation with parent in 1yr	0.357	0.297	0.391	0.363	0.465	0.386	45.30
	(0.15)**	(0.14)**	(0.171)**	(0.156)**	(0.185)**	(0.17)**	
Num. of excursions/exhibition visits in 1yr	$-$ 4.088	$-$ 4.522	$-$ 3.677	$-$ 3.973	$-$ 2.412	$-$ 4.284	$-$ 82.20
	(2.65)	(2.517)*	(2.823)	(2.556)	(2.896)	(2.721)	
Sees friends $>2$ times per week	0.052	0.072	0.019	0.037	$-$ 0.082	$-$ 0.052	9.10
	(0.165)	(0.167)	(0.179)	(0.179)	(0.185)	(0.186)	
Received childcare in past 7 days	0.061	0.065	0.001	0.025	0.015	0.033	27.10
	(0.149)	(0.15)	(0.156)	(0.152)	(0.167)	(0.163)	
Received childcare from a relative in past 7 days	$-$ 0.065	$-$ 0.047	$-$ 0.114	$-$ 0.078	$-$ 0.1	$-$ 0.068	$-$ 31.40
	(0.126)	(0.12)	(0.132)	(0.124)	(0.138)	(0.128)	
Mothers subjective life satisfaction (1$-$ lowest, 5$-$ highest)	$-$ 0.041	0.181	0.077	0.218	0.347	0.546	2
	(0.382)	(0.369)	(0.394)	(0.379)	(0.412)	(0.41)	
Vegetables/legumes (in kg)	$-$ 2.029	$-$ 2.105	$-$ 2.523	$-$ 2.858	$-$ 2.74	$-$ 3.32	$-$ 80.10
	(1.879)	(2.052)	(2.029)	(2.177)	(2.022)	(2.215)	
Fruit (fresh and canned) (in kg)	$-$ 1.412	$-$ 1.315	$-$ 0.721	$-$ 0.629	$-$ 1.199	$-$ 1.364	$-$ 37.70
	(1.236)	(1.287)	(1.328)	(1.354)	(1.443)	(1.456)	
Meat and poultry (in kg)	$-$ 0.689	$-$ 1.658	$-$ 0.261	$-$ 1.029	$-$ 0.732	$-$ 1.038	$-$ 42.90
	(1.028)	(1.008)	(1.128)	(1.114)	(1.152)	(1.138)	
Dairy (in kg)	3.267	1.915	3.216	1.984	2.553	1.448	44.30
	(1.693)*	(1.491)	(1.853)*	(1.628)	(1.865)	(1.684)	
Vodka and liquors (in litres)	0.042	0.003	0.121	0.062	0.178	0.136	20.70
	(0.156)	(0.143)	(0.167)	(0.161)	(0.187)	(0.193)	
Refined sugar (in kg)	$-$ 0.073	0.104	0.436	0.448	0.779	1.35	1.40
	(0.646)	(0.657)	(0.722)	(0.726)	(0.744)	(0.746)*	
Candy and high-sugar treats (in kg)	0.374	0.528	0.596	0.544	0.534	0.414	30
	(0.415)	(0.43)	(0.472)	(0.495)	(0.506)	(0.524)	
Starches (in kg)	0.469	0.303	1.59	0.637	$-$ 0.41	1.177	4.30
	(2.608)	(2.382)	(2.961)	(2.79)	(3.015)	(2.705)	
*Age controls*	NO	YES	YES	YES	YES	YES	
*Other controls*	NO	YES	NO	YES	NO	YES	
*Linear trend*	YES	YES	YES	YES	YES	YES	
*Quadratic trend*	NO	NO	YES	YES	YES	YES	
*Cubic trend*	NO	NO	NO	NO	YES	YES	
*RLMS year controls*	YES	YES	YES	YES	YES	YES	
Num. obs.	163	163	345	345	482	482	

***− 1 % sign., **− 5% sign., *− 10% sign. Coefficient std. errors are given in parentheses under the coefficient. Error terms are clustered at the regional level. Relative effect sizes are calculated relative to sample means in MC-ineligible families, based on average effects in models with a 12-month bandwidth

, 22

**Table 22:** Regression discontinuity estimates of impact of MC on 2nd child educational and time spending outcome (12, 24 and 36 months’ window at 1 January 2007 cut-off birth date)

Variable	RDD 12m	RDD 12m	RDD 24m	RDD 24m	RDD 36m	RDD 36m	Relative effect size (%)
	(1)	(2)	(3)	(4)	(5)	(6)	
Clothes (in Rub)	$-$ 0.673	$-$ 0.834	0.06	$-$ 0.246	$-$ 0.023	$-$ 0.228	$-$ 16.30
	(1.171)	(1.086)	(1.364)	(1.256)	(1.403)	(1.283)	
Essential food items (in Rub)	2.172	0.853	4.129	2.742	4.027	3.665	17
	(2.011)	(1.759)	(2.284)*	(2.146)	(2.365)*	(2.298)	
Utilities (in Rub)	0.409	0.059	0.411	0.089	1.305	0.968	6.80
	(1.268)	(1.237)	(1.568)	(1.426)	(1.735)	(1.658)	
Essential services (in Rub)	0.078	$-$ 0.13	$-$ 0.068	$-$ 0.369	0.475	0.051	$-$ 1.10
	(1.249)	(1.122)	(1.169)	(1.132)	(1.151)	(1.115)	
All basic expenditure (in Rub)	1.987	$-$ 0.052	4.531	2.215	5.784	4.457	5
	(3.775)	(3.255)	(4.103)	(3.582)	(4.268)	(3.933)	
All non-essential expenditure (in Rub)	$-$ 0.275	0.972	1.401	1.471	3.761	2.572	6.60
	(2.266)	(4.154)	(2.44)	(2.592)	(2.473)	(2.805)	
Purchase of durable goods (in Rub)	$-$ 0.092	$-$ 0.143	$-$ 0.011	$-$ 0.058	0.062	0.009	$-$ 29.60
	(0.166)	(0.175)	(0.177)	(0.177)	(0.184)	(0.187)	
Sh. Essential food items	0.015	0.017	0.076	0.082	0.083	0.127	5.60
	(0.077)	(0.071)	(0.087)	(0.084)	(0.091)	(0.085)	
Sh. Clothes	$-$ 0.055	$-$ 0.016	$-$ 0.055	$-$ 0.022	$-$ 0.055	$-$ 0.01	$-$ 24.30
	(0.048)	(0.05)	(0.049)	(0.044)	(0.05)	(0.043)	
Sh. Non-essential expenditure	$-$ 0.014	0.047	0.013	0.028	$-$ 0.016	$-$ 0.037	10.10
	(0.09)	(0.159)	(0.08)	(0.094)	(0.139)	(0.167)	
Sh. Savings	$-$ 0.015	$-$ 0.006	$-$ 0.013	$-$ 0.019	$-$ 0.024	$-$ 0.039	$-$ 27.40
	(0.033)	(0.035)	(0.035)	(0.035)	(0.035)	(0.037)	
Sh. Loan payments	0.104	0.105	0.104	0.113	0.135	0.137	118.90
	(0.054)*	(0.056)*	(0.063)*	(0.069)	(0.077)*	(0.085)	
Sh. Utilities	$-$ 0.003	$-$ 0.011	$-$ 0.008	$-$ 0.006	$-$ 0.005	$-$ 0.001	$-$ 7.90
	(0.032)	(0.031)	(0.034)	(0.032)	(0.033)	(0.032)	
Sh. Essential services	$-$ 0.042	$-$ 0.04	$-$ 0.046	$-$ 0.039	$-$ 0.039	$-$ 0.031	$-$ 59.80
	(0.039)	(0.04)	(0.041)	(0.043)	(0.043)	(0.044)	
Sh. Basic expenditure	$-$ 0.086	$-$ 0.05	$-$ 0.033	0.015	$-$ 0.015	0.085	$-$ 11.50
	(0.131)	(0.124)	(0.139)	(0.129)	(0.148)	(0.133)	
Household savings in last 30 days (in 2011 prices)	0.424	1.04	1.239	0.51	0.689	$-$ 0.768	25.50
	(1.878)	(2.319)	(1.839)	(1.776)	(1.603)	(1.624)	
Savings will last several months	$-$ 0.073	$-$ 0.051	$-$ 0.039	$-$ 0.038	0.02	0.008	$-$ 42.80
	(0.122)	(0.12)	(0.138)	(0.138)	(0.151)	(0.151)	
Loan payments in last 30 days (in 2011 prices)	7.127	6.259	5.766	5.037	6.737	5.763	220.40
	(3.839)*	(3.568)*	(3.877)	(3.505)	(3.645)*	(3.433)*	
Borrowed from a private individual in last 12 months	0.249	0.215	0.271	0.276	0.209	0.206	282.30
	(0.101)**	(0.102)**	(0.107)**	(0.105)***	(0.111)*	(0.114)*	
Loan to a private individual in last 12 months	0.015	0.011	0.035	0.043	$-$ 0.004	0	11.70
	(0.137)	(0.135)	(0.146)	(0.144)	(0.148)	(0.146)	
Household owns home	$-$ 0.026	$-$ 0.03	0.047	0.061	0.046	0.029	$-$ 3.50
	(0.119)	(0.113)	(0.13)	(0.126)	(0.137)	(0.136)	
Hot water supply	0.197	0.062	0.165	0.136	0.256	0.185	23.60
	(0.178)	(0.133)	(0.189)	(0.138)	(0.194)	(0.149)	
Central sewerage	0.191	0.091	0.202	0.203	0.279	0.249	23.40
	(0.179)	(0.093)	(0.188)	(0.104)*	(0.191)	(0.112)**	
Central heating	0.215	0.091	0.196	0.161	0.26	0.202	27.20
	(0.175)	(0.112)	(0.186)	(0.112)	(0.191)	(0.117)*	
*Age controls*	NO	YES	YES	YES	YES	YES	
*Other controls*	NO	YES	NO	YES	NO	YES	
*Linear trend*	YES	YES	YES	YES	YES	YES	
*Quadratic trend*	NO	NO	YES	YES	YES	YES	
*Cubic trend*	NO	NO	NO	NO	YES	YES	
*RLMS year controls*	YES	YES	YES	YES	YES	YES	
Num. obs.	163	163	345	345	482	482	

***− 1 % sign., **− 5% sign., *− 10% sign. Coefficient std. errors are given in parentheses under the coefficient. Error terms are clustered at the regional level. Relative effect sizes are calculated relative to sample means in MC-ineligible families, based on average effects in models with a 12-month bandwidth

, 23

**Table 23:** Regression discontinuity estimates of the MC impact on household expenditures, expressed as a share of self-disclosed household income earned in the last 30 days (12, 24 and 36 months’ window at 1 January 2007 cut-off birth date)

Variable	RDD 12m	RDD 12m	RDD 12m	RDD 24m	RDD 24m	RDD 36m	RDD 36m
	(1)	(2)	(3)	(4)	(5)	(6)	(7)
Sh. Essential food items	0.072	0.074	0.038	0.078	0.057	0.129	0.129
	(0.065)	(0.064)	(0.061)	(0.07)	(0.066)	(0.079)	(0.074)*
Sh. Clothes	$-$ 0.057	$-$ 0.054	$-$ 0.034	$-$ 0.076	$-$ 0.053	$-$ 0.044	$-$ 0.021
	(0.038)	(0.038)	(0.038)	(0.044)*	(0.039)	(0.051)	(0.047)
Sh. Non-essential expenditure	0.012	0.017	0.043	0.082	0.094	0.067	0.054
	(0.055)	(0.055)	(0.064)	(0.056)	(0.068)	(0.091)	(0.105)
Sh. Savings	0.003	0.004	0.025	0.004	0.016	0.007	0.008
	(0.043)	(0.043)	(0.061)	(0.045)	(0.05)	(0.046)	(0.05)
Sh. Loan payments	0.079	0.081	0.075	0.057	0.067	0.097	0.092
	(0.036)**	(0.036)**	(0.037)**	(0.043)	(0.042)	(0.052)*	(0.053)*
Sh. Utilities	0.031	0.03	0.027	0.029	0.04	0.05	0.054
	(0.026)	(0.026)	(0.026)	(0.028)	(0.028)	(0.039)	(0.036)
Sh. Essential services	$-$ 0.007	$-$ 0.007	$-$ 0.007	$-$ 0.013	$-$ 0.011	$-$ 0.007	$-$ 0.003
	(0.024)	(0.024)	(0.024)	(0.026)	(0.027)	(0.03)	(0.03)
Sh. Basic expenditure	0.039	0.043	0.024	0.017	0.035	0.127	0.158
	(0.1)	(0.1)	(0.093)	(0.111)	(0.1)	(0.133)	(0.118)
*Age controls*	NO	YES	YES	YES	YES	YES	YES
*Other controls*	NO	NO	YES	NO	YES	NO	YES
*Linear trend*	YES	YES	YES	YES	YES	YES	YES
*Quadratic trend*	NO	NO	NO	YES	YES	YES	YES
*Cubic trend*	NO	NO	NO	NO	NO	YES	YES
*RLMS year controls*	YES	YES	YES	YES	YES	YES	YES
Num. obs.	328	328	328	727	727	1047	1047

***− 1 % sign., **− 5% sign., *− 10% sign. Coefficient std. errors are given in parentheses under the coefficient. Error terms are clustered at regional level

, 24

**Table 24:** Regression discontinuity estimates of the MC impact on the 2nd child, by child gender (12-, 24-, and 36-month window around the cut-off birth date of 1 January 2007)

Outcome	Female						Male					
	RDD12m	RDD12m	RDD12m	RDD24m	RDD24m	RDD36m	RDD12m	RDD12m	RDD12m	RDD24m	RDD24m	RDD36m
	(1)	(2)	(3)	(4)	(5)	(6)	(7)	(8)	(9)	(10)	(11)	(12)
In good/excellent health	0.158	0.158	0.184	0.098	0.225	0.199	$-$ 0.208	$-$ 0.194	$-$ 0.024	0.063	0.09	$-$ 0.024
	(0.097)	(0.098)	(0.111)*	(0.083)	(0.111)**	(0.126)	(0.152)	(0.154)	(0.161)	(0.115)	(0.154)	(0.167)
Health score (1-highest, 5-lowest)	$-$ 0.218	$-$ 0.217	$-$ 0.221	$-$ 0.139	$-$ 0.319	$-$ 0.241	0.189	0.183	$-$ 0.017	$-$ 0.133	$-$ 0.076	0.054
	(0.117)*	(0.119)*	(0.124)*	(0.098)	(0.132)**	(0.153)	(0.157)	(0.162)	(0.165)	(0.123)	(0.163)	(0.177)
Num. of chronic conditions	$-$ 0.412	$-$ 0.405	$-$ 0.4	$-$ 0.163	$-$ 0.538	$-$ 0.485	0.173	0.15	0.107	0.109	$-$ 0.194	$-$ 0.06
	(0.139)***	(0.139)***	(0.144)***	(0.122)	(0.156)***	(0.185)***	(0.222)	(0.236)	(0.305)	(0.176)	(0.284)	(0.282)
Health problem in last 30d	0.17	0.168	0.143	0.059	0.193	0.252	$-$ 0.025	0.008	0.036	$-$ 0.117	$-$ 0.196	$-$ 0.045
	(0.161)	(0.162)	(0.161)	(0.11)	(0.17)	(0.194)	(0.205)	(0.206)	(0.209)	(0.13)	(0.218)	(0.244)
Extracurricular study (in mins per week)	3.206	$-$ 0.199	8.856	6.659	13.753	15.156	15.235	29.289	18.342	18.482	27.372	7.326
	(10.908)	(9.926)	(11.77)	(8.553)	(10.158)	(11.825)	(21.55)	(26.564)	(25.945)	(15.615)	(20.657)	(23.961)
Extracurricular arts (in mins per week)	162.388	151.307	183.056	54.92	204.001	241.799	$-$ 44.279	$-$ 34.223	$-$ 94.449	7.518	$-$ 70.867	$-$ 50.42
	(68.057)**	(60.564)**	(59.433)***	(50.891)	(65.426)***	(69.899)***	(61.055)	(62.072)	(96.079)	(49.495)	(79.156)	(75.335)
Watching TV/on Internet (in mins per week)	11.526	13.166	$-$ 8.482	$-$ 10.101	1.855	10.798	$-$ 0.616	2.753	$-$ 11.507	24.022	25.61	$-$ 0.483
	(24.245)	(24.75)	(30.369)	(21.061)	(27.285)	(30.482)	(36.203)	(37.387)	(46.357)	(27.473)	(39.912)	(42.378)
Extracurricular sports (in mins per week)	71.131	57.835	45.124	$-$ 66.802	$-$ 20.178	54.03	$-$ 97.538	$-$ 140.131	$-$ 139.03	$-$ 165.456	$-$ 198.727	$-$ 81.786
	(99.642)	(96.027)	(105.578)	(82.063)	(102.318)	(108.249)	(194.881)	(198.18)	(204.563)	(150.22)	(209.978)	(222.188)
Used a personal computer	0.126	0.094	0.079	$-$ 0.014	0.192	0.272	0.275	0.306	0.286	0.121	0.454	0.266
	(0.175)	(0.184)	(0.184)	(0.126)	(0.176)	(0.197)	(0.28)	(0.264)	(0.244)	(0.18)	(0.239)*	(0.248)
Vacation with parent in 1yr	$-$ 0.222	$-$ 0.232	$-$ 0.158	$-$ 0.278	0.023	0.022	0.315	0.33	0.412	0.027	0.405	0.522
	(0.176)	(0.171)	(0.164)	(0.111)**	(0.167)	(0.188)	(0.19)*	(0.177)*	(0.161)**	(0.134)	(0.192)**	(0.21)**
Num. of excursions/exhibition visits in 1yr	$-$ 5.997	$-$ 6.074	$-$ 5.205	$-$ 1.967	$-$ 3.523	$-$ 4.616	0.687	1.193	0.266	1.363	1.233	$-$ 0.196
	(2.419)**	(2.405)**	(2.366)**	(1.465)	(2.478)	(2.894)	(1.831)	(1.801)	(1.763)	(1.308)	(1.817)	(2.083)
Sees friends $>2$ times per week	$-$ 0.044	$-$ 0.046	$-$ 0.039	$-$ 0.114	$-$ 0.228	$-$ 0.272	$-$ 0.407	$-$ 0.451	$-$ 0.431	$-$ 0.135	$-$ 0.448	$-$ 0.376
	(0.17)	(0.171)	(0.177)	(0.113)	(0.179)	(0.201)	(0.181)**	(0.178)**	(0.197)**	(0.124)	(0.186)**	(0.208)*
Received childcare in past 7 days	$-$ 0.116	$-$ 0.115	$-$ 0.044	$-$ 0.182	$-$ 0.218	$-$ 0.185	0.277	0.261	0.179	0.163	0.242	0.326
	(0.131)	(0.133)	(0.143)	(0.095)*	(0.145)	(0.166)	(0.178)	(0.181)	(0.185)	(0.118)	(0.175)	(0.196)*
Received childcare from a relative in past 7 days	$-$ 0.139	$-$ 0.138	$-$ 0.154	$-$ 0.129	$-$ 0.244	$-$ 0.168	0.083	0.069	0.068	0.047	0.145	0.273
	(0.101)	(0.1)	(0.109)	(0.074)*	(0.12)**	(0.137)	(0.13)	(0.131)	(0.141)	(0.094)	(0.141)	(0.144)*
Mothers subjective life satisfaction (1-lowest, 5-highest)	0.188	0.187	0.433	$-$ 0.019	0.415	0.431	$-$ 0.381	$-$ 0.353	0.031	$-$ 0.219	0.51	0.687
	(0.381)	(0.383)	(0.377)	(0.25)	(0.37)	(0.409)	(0.388)	(0.371)	(0.383)	(0.236)	(0.373)	(0.419)
*Age controls*	NO	YES	YES	YES	YES	YES	NO	YES	YES	YES	YES	YES
*Other controls*	NO	NO	YES	NO	YES	YES	NO	NO	YES	NO	NO	YES
*Linear trend*	YES	YES	YES	YES	YES	YES	YES	YES	YES	YES	YES	YES
*Quadratic trend*	NO	NO	NO	NO	NO	YES	NO	NO	NO	NO	NO	YES
*Cubic trend*	NO	NO	NO	NO	NO	YES	NO	NO	NO	NO	NO	YES
*RLMS year controls*	YES	YES	YES	YES	YES	YES	YES	YES	YES	YES	YES	YES
Num. obs.	173	173	173	390	390	543	155	155	155	337	337	504

***− 1 % sign., **− 5% sign., *− 10% sign. Coefficient std. errors are given in parentheses under the coefficient. Errors are clustered at regional level

, 25

**Table 25:** Regression discontinuity estimates of the MC impact on the 2nd child, by rural/urban residence status (12-, 24-, and 36-month window around the cut-off birth date of 1 January 2007)

Outcome	Rural						Urban					
	RDD12m	RDD12m	RDD12m	RDD24m	RDD24m	RDD36m	RDD12m	RDD12m	RDD12m	RDD24m	RDD24m	RDD36m
	(1)	(2)	(3)	(4)	(5)	(6)	(7)	(8)	(9)	(10)	(11)	(12)
In good/excellent health	0.202	0.212	0.269	0.123	0.325	0.252	$-$ 0.145	$-$ 0.144	0.032	0.009	0.044	$-$ 0.095
	(0.144)	(0.146)	(0.15)*	(0.105)	(0.142)**	(0.168)	(0.117)	(0.119)	(0.128)	(0.089)	(0.128)	(0.144)
Health score (1-highest, 5$-$ lowest)	$-$ 0.198	$-$ 0.21	$-$ 0.305	$-$ 0.228	$-$ 0.332	$-$ 0.21	0.103	0.103	$-$ 0.075	$-$ 0.015	$-$ 0.108	0.085
	(0.151)	(0.154)	(0.159)*	(0.115)**	(0.151)**	(0.176)	(0.13)	(0.13)	(0.135)	(0.099)	(0.148)	(0.166)
Num. of chronic conditions	$-$ 0.371	$-$ 0.362	$-$ 0.526	$-$ 0.421	$-$ 0.636	$-$ 0.522	0.045	0.051	$-$ 0.317	0.194	$-$ 0.349	$-$ 0.315
	(0.21)*	(0.211)*	(0.203)**	(0.157)***	(0.202)***	(0.223)**	(0.179)	(0.184)	(0.234)	(0.138)	(0.223)	(0.24)
Health problem in last 30d	$-$ 0.076	$-$ 0.087	$-$ 0.054	$-$ 0.153	$-$ 0.219	$-$ 0.049	0.155	0.154	0.141	0.06	0.103	0.226
	(0.196)	(0.197)	(0.229)	(0.125)	(0.213)	(0.241)	(0.151)	(0.15)	(0.148)	(0.103)	(0.161)	(0.177)
Extracurricular study (in mins per week)	27.399	24.04	27.997	$-$ 2.923	6.28	5.465	$-$ 4.655	$-$ 8.097	8.149	23.739	32.751	22.544
	(21.316)	(18.301)	(25.573)	(5.211)	(7.832)	(9.31)	(12.449)	(14.578)	(17.45)	(13.396)*	(18.946)*	(20.748)
Extracurricular arts (in mins per week)	27.918	20.401	11.771	$-$ 19.292	44.087	82.958	39.826	50.158	66.532	18.518	72.898	73.116
	(48.236)	(47.028)	(60.021)	(45.699)	(58.688)	(67.753)	(66.746)	(66.515)	(77.93)	(52.717)	(71.999)	(75.195)
Watching TV/on Internet (in mins per week)	3.423	2.425	30.967	$-$ 7.695	25.421	32.464	19.036	23.79	8.935	17.973	10.882	8.884
	(32.342)	(31.897)	(34.709)	(21.75)	(31.783)	(36.061)	(25.271)	(25.129)	(29.292)	(23.699)	(29.298)	(31.878)
Extracurricular sports (in mins per week)	110.186	118.651	$-$ 33.864	$-$ 110.289	$-$ 84.205	39.199	92.673	92.443	73.597	$-$ 66.013	24.823	46.951
	(142.219)	(147.922)	(167.649)	(122.515)	(155.083)	(169.461)	(136.188)	(137.897)	(154.761)	(95.841)	(139.957)	(150.026)
Used a personal computer	$-$ 0.158	$-$ 0.125	$-$ 0.044	$-$ 0.08	0.072	0.042	0.245	0.235	0.29	0.077	0.271	0.235
	(0.237)	(0.225)	(0.222)	(0.156)	(0.217)	(0.252)	(0.174)	(0.17)	(0.177)	(0.121)	(0.175)	(0.19)
Vacation with parent in 1yr	$-$ 0.078	$-$ 0.087	$-$ 0.209	$-$ 0.277	0.244	0.195	0.055	0.053	0.119	0.003	0.157	0.285
	(0.214)	(0.216)	(0.238)	(0.139)**	(0.211)	(0.246)	(0.137)	(0.138)	(0.143)	(0.092)	(0.156)	(0.171)*
Num. of excursions/exhibition visits in 1yr	$-$ 2.146	$-$ 2.244	$-$ 2.758	$-$ 1.03	$-$ 1.91	$-$ 2.468	$-$ 5.696	$-$ 5.715	$-$ 3.845	$-$ 0.593	$-$ 2.094	$-$ 3.983
	(1.12)*	(1.12)**	(1.097)**	(0.753)	(1.166)	(1.349)*	(2.558)**	(2.554)**	(2.786)	(1.585)	(2.992)	(3.308)
Sees friends $>2$ times per week	$-$ 0.129	$-$ 0.145	$-$ 0.163	$-$ 0.054	$-$ 0.166	$-$ 0.172	$-$ 0.304	$-$ 0.3	$-$ 0.26	$-$ 0.223	$-$ 0.403	$-$ 0.477
	(0.192)	(0.181)	(0.193)	(0.122)	(0.183)	(0.216)	(0.153)**	(0.154)*	(0.166)	(0.107)**	(0.17)**	(0.18)***
Received childcare in past 7 days	0.165	0.173	0.042	$-$ 0.016	0.096	0.076	$-$ 0.022	$-$ 0.021	$-$ 0.05	$-$ 0.023	$-$ 0.095	$-$ 0.001
	(0.157)	(0.154)	(0.152)	(0.113)	(0.151)	(0.184)	(0.151)	(0.153)	(0.162)	(0.101)	(0.166)	(0.186)
Received childcare from a relative in past 7 days	$-$ 0.064	$-$ 0.052	$-$ 0.154	$-$ 0.15	$-$ 0.077	$-$ 0.049	$-$ 0.025	$-$ 0.025	$-$ 0.019	0.007	$-$ 0.061	0.117
	(0.14)	(0.135)	(0.134)	(0.098)	(0.139)	(0.155)	(0.118)	(0.118)	(0.122)	(0.078)	(0.126)	(0.14)
Mothers subjective life satisfaction (1-lowest, 5-highest)	0.565	0.595	0.315	0.109	0.737	0.59	$-$ 0.219	$-$ 0.229	0.107	$-$ 0.198	0.365	0.556
	(0.426)	(0.413)	(0.425)	(0.285)	(0.405)*	(0.463)	(0.328)	(0.321)	(0.323)	(0.207)	(0.338)	(0.376)
*Age controls*	NO	YES	YES	YES	YES	YES	NO	YES	YES	YES	YES	YES
*Other controls*	NO	NO	YES	NO	YES	YES	NO	NO	YES	NO	NO	YES
*Linear trend*	YES	YES	YES	YES	YES	YES	YES	YES	YES	YES	YES	YES
*Quadratic trend*	NO	NO	NO	NO	NO	YES	NO	NO	NO	NO	NO	YES
*Cubic trend*	NO	NO	NO	NO	NO	YES	NO	NO	NO	NO	NO	YES
*RLMS year controls*	YES	YES	YES	YES	YES	YES	YES	YES	YES	YES	YES	YES
Num. obs.	127	127	127	332	332	434	138	138	138	395	395	613

***− 1 % sign., **− 5% sign., *− 10% sign. Coefficient std. errors are given in parentheses under the coefficient. Errors are clustered at regional level

,26

**Table 26:** Regression discontinuity estimates of the MC impact on the 2nd child, by rural/urban residence status (12-, 24-, and 36-month window around the cut-off birth date of 1 January 2007), continued

Outcome	Rural						Urban					
	RDD12m	RDD12m	RDD12m	RDD24m	RDD24m	RDD36m	RDD12m	RDD12m	RDD12m	RDD24m	RDD24m	RDD36m
	(1)	(2)	(3)	(4)	(5)	(6)	(7)	(8)	(9)	(10)	(11)	(12)
Essential food items (in Rub)	$-$ 1.187	$-$ 1.316	$-$ 2.929	$-$ 0.836	$-$ 0.167	$-$ 0.435	2.087	2.021	1.72	2.12	3.884	5.238
	(2.483)	(2.552)	(2.441)	(1.751)	(2.761)	(3.283)	(1.712)	(1.627)	(1.73)	(1.26)*	(1.938)**	(2.087)**
Clothes (in Rub)	$-$ 1.082	$-$ 1.034	$-$ 1.3	$-$ 0.722	$-$ 1.081	$-$ 0.643	$-$ 2.645	$-$ 2.662	$-$ 1.56	$-$ 1.049	$-$ 0.786	$-$ 1.305
	(1.177)	(1.18)	(1.086)	(0.867)	(1.245)	(1.442)	(1.243)**	(1.234)**	(1.103)	(0.978)	(1.337)	(1.415)
Utilities (in Rub)	$-$ 0.502	$-$ 0.647	$-$ 0.832	$-$ 0.799	$-$ 0.311	$-$ 0.893	1.604	1.574	1.991	1.205	3.067	2.854
	(0.709)	(0.563)	(0.674)	(0.427)*	(0.693)	(0.817)	(1.462)	(1.425)	(1.42)	(0.97)	(1.586)*	(1.775)
Essential services (in Rub)	$-$ 2.079	$-$ 2.14	$-$ 2.411	$-$ 1.5	$-$ 1.603	$-$ 1.914	1.589	1.579	1.767	0.932	1.156	1.736
	(1.053)*	(1.051)**	(1.122)**	(0.649)**	(1.189)	(1.404)	(1.239)	(1.23)	(1.038)*	(0.967)	(1.251)	(1.222)
All basic expenditure (in Rub)	$-$ 4.851	$-$ 5.136	$-$ 7.471	$-$ 3.857	$-$ 3.162	$-$ 3.885	2.634	2.512	3.918	3.207	7.322	8.523
	(3.453)	(3.492)	(3.22)**	(2.445)	(3.538)	(4.3)	(3.554)	(3.345)	(3.186)	(2.5)	(3.458)**	(3.773)**
All non-essential expenditure (in Rub)	$-$ 0.779	$-$ 0.553	3.109	$-$ 3.59	2.535	0.473	$-$ 0.698	$-$ 0.695	0.275	3.027	5.723	5.341
	(2.131)	(2.193)	(4.187)	(4.069)	(2.897)	(3.603)	(2.303)	(2.315)	(2.661)	(2.188)	(3.132)*	(3.165)*
Purchase of durable goods (in Rub)	$-$ 0.174	$-$ 0.19	$-$ 0.284	$-$ 0.205	$-$ 0.129	$-$ 0.139	0.088	0.089	0.119	$-$ 0.023	0.164	0.166
	(0.179)	(0.183)	(0.195)	(0.122)*	(0.172)	(0.2)	(0.16)	(0.161)	(0.169)	(0.109)	(0.177)	(0.196)
Sh. Essential food items	0.129	0.123	0.046	$-$ 0.016	0.075	0.174	0.048	0.048	0.025	0.105	0.078	0.118
	(0.133)	(0.131)	(0.118)	(0.089)	(0.129)	(0.154)	(0.06)	(0.06)	(0.064)	(0.052)**	(0.066)	(0.071)*
Sh. Clothes	$-$ 0.038	$-$ 0.036	0.003	$-$ 0.028	$-$ 0.072	0.047	$-$ 0.065	$-$ 0.064	$-$ 0.054	$-$ 0.017	$-$ 0.039	$-$ 0.057
	(0.077)	(0.079)	(0.065)	(0.055)	(0.082)	(0.102)	(0.035)*	(0.036)*	(0.027)*	(0.023)	(0.031)	(0.035)
Sh. Non-essential expenditure	0.035	0.042	0.163	$-$ 0.171	0.099	$-$ 0.087	$-$ 0.01	$-$ 0.01	$-$ 0.018	0.062	0.063	0.048
	(0.111)	(0.111)	(0.166)	(0.147)	(0.118)	(0.275)	(0.067)	(0.067)	(0.088)	(0.041)	(0.064)	(0.065)
Sh. Savings	0.025	0.027	0.084	0.033	0.018	0.011	$-$ 0.012	$-$ 0.012	$-$ 0.006	0.016	0.006	0.006
	(0.102)	(0.103)	(0.16)	(0.069)	(0.11)	(0.118)	(0.009)	(0.009)	(0.013)	(0.022)	(0.022)	(0.021)
Sh. Loan payments	0.029	0.031	0.041	$-$ 0.01	0.022	0.033	0.113	0.114	0.127	0.105	0.098	0.128
	(0.05)	(0.05)	(0.056)	(0.045)	(0.08)	(0.102)	(0.052)**	(0.052)**	(0.057)**	(0.04)***	(0.054)*	(0.066)*
Sh. Utilities	$-$ 0.023	$-$ 0.026	$-$ 0.016	$-$ 0.021	$-$ 0.029	$-$ 0.041	0.064	0.065	0.063	0.054	0.09	0.108
	(0.03)	(0.028)	(0.024)	(0.02)	(0.032)	(0.035)	(0.039)	(0.039)	(0.04)	(0.025)**	(0.043)**	(0.054)**
Sh. Essential services	$-$ 0.067	$-$ 0.068	$-$ 0.083	$-$ 0.041	$-$ 0.051	$-$ 0.043	0.031	0.031	0.028	0.032	0.014	0.028
	(0.049)	(0.05)	(0.053)	(0.026)	(0.052)	(0.066)	(0.022)	(0.022)	(0.018)	(0.021)	(0.028)	(0.022)
Sh. Basic expenditure	0.001	$-$ 0.007	$-$ 0.05	$-$ 0.106	$-$ 0.077	0.137	0.078	0.08	0.063	0.175	0.143	0.197
	(0.191)	(0.191)	(0.159)	(0.129)	(0.19)	(0.237)	(0.107)	(0.108)	(0.097)	(0.085)**	(0.098)	(0.117)*
*Age controls*	NO	YES	YES	YES	YES	YES	NO	YES	YES	YES	YES	YES
*Other controls*	NO	NO	YES	NO	YES	YES	NO	NO	YES	NO	NO	YES
*Linear trend*	YES	YES	YES	YES	YES	YES	YES	YES	YES	YES	YES	YES
*Quadratic trend*	NO	NO	NO	NO	NO	YES	NO	NO	NO	NO	NO	YES
*Cubic trend*	NO	NO	NO	NO	NO	YES	NO	NO	NO	NO	NO	YES
*RLMS year controls*	YES	YES	YES	YES	YES	YES	YES	YES	YES	YES	YES	YES
Num. obs.	127	127	127	332	332	434	138	138	138	395	395	613

***− 1 % sign., **− 5% sign., *− 10% sign. Coefficient std. errors are given in parentheses under the coefficient. Error terms are clustered at regional level

, 27

**Table 27:** Regression discontinuity estimates of the MC impact on the 2nd child, by rural/urban residence status (12-, 24-, and 36-month window around the cut-off birth date of 1 January 2007), continued

Outcome	Rural						Urban					
	RDD12m	RDD12m	RDD12m	RDD24m	RDD24m	RDD36m	RDD12m	RDD12m	RDD12m	RDD24m	RDD24m	RDD36m
	(1)	(2)	(3)	(4)	(5)	(6)	(7)	(8)	(9)	(10)	(11)	(12)
Vegetables/legumes (in kg)	$-$ 1.983	$-$ 1.962	$-$ 2.332	$-$ 0.261	$-$ 2.023	$-$ 3.655	$-$ 0.081	$-$ 0.096	0.02	0.179	$-$ 0.031	$-$ 0.304
	(3.204)	(3.219)	(3.343)	(1.646)	(3.302)	(4.013)	(1.254)	(1.229)	(1.242)	(0.937)	(1.544)	(1.499)
Fruit (fresh and canned) (in kg)	$-$ 1.892	$-$ 1.906	$-$ 2.182	$-$ 1.933	$-$ 2.455	$-$ 3.856	0.002	$-$ 0.013	0.195	1.297	1.761	1.57
	(2.041)	(2.035)	(2.189)	(1.058)*	(1.727)	(1.976)*	(0.963)	(0.951)	(1.018)	(0.658)**	(1.091)	(1.255)
Meat and poultry (in kg)	$-$ 2.508	$-$ 2.649	$-$ 3.877	$-$ 1.198	$-$ 1.589	$-$ 2.766	0.655	0.675	0.016	0.46	0.896	1.345
	(1.078)**	(1.071)**	(1.078)***	(0.798)	(1.225)	(1.412)*	(0.814)	(0.787)	(0.998)	(0.677)	(0.986)	(1.032)
Dairy (in kg)	1.573	1.355	0.083	$-$ 0.537	0.299	$-$ 0.158	1.04	1.027	0.776	$-$ 0.994	1.355	0.962
	(1.639)	(1.648)	(1.694)	(1.129)	(1.568)	(1.866)	(1.415)	(1.41)	(1.473)	(1.054)	(1.608)	(1.754)
Vodka and liquors (in litres)	$-$ 0.007	$-$ 0.009	$-$ 0.01	$-$ 0.028	0.14	0.131	$-$ 0.046	$-$ 0.048	$-$ 0.072	$-$ 0.117	$-$ 0.124	$-$ 0.112
	(0.05)	(0.048)	(0.052)	(0.065)	(0.112)	(0.113)	(0.202)	(0.201)	(0.208)	(0.125)	(0.239)	(0.279)
Refined sugar (in kg)	0.359	0.465	0.083	$-$ 0.52	0.336	0.645	0.571	0.572	0.456	0.109	0.628	1.128
	(1.02)	(1.037)	(1.135)	(0.746)	(1.074)	(1.143)	(0.423)	(0.425)	(0.441)	(0.305)	(0.445)	(0.515)**
Candy and high-sugar treats (in kg)	$-$ 0.43	$-$ 0.388	$-$ 0.11	$-$ 0.102	$-$ 0.222	$-$ 0.194	0.588	0.585	0.23	0.102	0.614	0.51
	(0.676)	(0.661)	(0.735)	(0.467)	(0.783)	(0.883)	(0.431)	(0.432)	(0.501)	(0.292)	(0.547)	(0.607)
Starches (in kg)	0.036	0.021	0.423	1.269	0.212	1.367	0.627	0.689	$-$ 0.969	1.529	$-$ 1.16	$-$ 1.62
	(3.811)	(3.818)	(3.461)	(2.453)	(3.422)	(3.862)	(2.067)	(2.064)	(2.186)	(1.544)	(2.394)	(2.486)
Household savings in last 30 days (in 2011 prices)	$-$ 0.247	$-$ 0.2	0.674	$-$ 0.14	$-$ 0.22	$-$ 0.552	$-$ 0.653	$-$ 0.683	$-$ 0.319	0.335	1.829	1.699
	(2.183)	(2.196)	(3.273)	(1.417)	(2.362)	(2.53)	(0.8)	(0.838)	(1.14)	(2.187)	(1.759)	(1.952)
Savings will last several months	$-$ 0.384	$-$ 0.383	$-$ 0.429	$-$ 0.136	$-$ 0.273	$-$ 0.402	0.047	0.05	0.157	0.082	0.09	0.241
	(0.177)**	(0.182)**	(0.179)**	(0.105)	(0.176)	(0.211)*	(0.13)	(0.127)	(0.121)	(0.089)	(0.127)	(0.139)*
Loan payments in last 30 days (in 2011 prices)	$-$ 0.027	$-$ 0.013	0.318	$-$ 0.332	$-$ 1.213	$-$ 1.938	5.501	5.52	7.524	5.015	5.704	7.184
	(1.538)	(1.562)	(1.674)	(1.222)	(1.545)	(1.894)	(3.055)*	(3.085)*	(3.166)**	(2.068)**	(3.007)*	(3.33)**
Consumer loan in last 12 months	$-$ 0.23	$-$ 0.224	$-$ 0.187	$-$ 0.119	$-$ 0.186	$-$ 0.08	0.163	0.166	0.163	0.163	0.069	0.123
	(0.133)*	(0.134)*	(0.145)	(0.09)	(0.151)	(0.174)	(0.116)	(0.114)	(0.13)	(0.089)*	(0.137)	(0.145)
Borrowed from a private individual in last 12 months	0.288	0.294	0.323	0.204	0.139	0.168	0.164	0.165	0.166	0.099	0.142	0.092
	(0.11)***	(0.111)***	(0.116)***	(0.073)***	(0.111)	(0.127)	(0.067)**	(0.067)**	(0.078)**	(0.054)*	(0.083)*	(0.089)
Loan to a private individual in last 12 months	$-$ 0.278	$-$ 0.28	$-$ 0.287	$-$ 0.21	$-$ 0.219	$-$ 0.282	0.042	0.042	0.063	0.101	$-$ 0.007	0.057
	(0.136)**	(0.136)**	(0.141)**	(0.077)***	(0.143)	(0.168)*	(0.093)	(0.093)	(0.094)	(0.069)	(0.096)	(0.105)
Household owns home	0.093	0.104	0.067	0.095	0.245	0.168	$-$ 0.143	$-$ 0.143	$-$ 0.13	0	$-$ 0.09	$-$ 0.094
	(0.121)	(0.116)	(0.123)	(0.09)	(0.136)*	(0.153)	(0.119)	(0.119)	(0.112)	(0.076)	(0.115)	(0.13)
Hot water supply	$-$ 0.247	$-$ 0.272	$-$ 0.287	$-$ 0.22	$-$ 0.16	$-$ 0.182	0.191	0.191	0.23	0.122	0.257	0.265
	(0.159)	(0.149)*	(0.157)*	(0.101)**	(0.162)	(0.187)	(0.134)	(0.126)	(0.132)*	(0.084)	(0.145)*	(0.158)*
Central sewerage	0.099	0.088	0.113	$-$ 0.227	0.418	0.415	0.08	0.079	0.174	0.056	0.18	0.178
	(0.09)	(0.082)	(0.105)	(0.084)***	(0.109)***	(0.135)***	(0.058)	(0.058)	(0.077)**	(0.058)	(0.09)**	(0.085)**
Central heating	0.249	0.235	0.255	0.064	0.319	0.357	$-$ 0.035	$-$ 0.035	0.029	0.012	0.051	0.073
	(0.103)**	(0.093)**	(0.118)**	(0.086)	(0.113)***	(0.129)***	(0.082)	(0.082)	(0.097)	(0.068)	(0.098)	(0.099)
*Age controls*	NO	YES	YES	YES	YES	YES	NO	YES	YES	YES	YES	YES
*Other controls*	NO	NO	YES	NO	YES	YES	NO	NO	YES	NO	NO	YES
*Linear trend*	YES	YES	YES	YES	YES	YES	YES	YES	YES	YES	YES	YES
*Quadratic trend*	NO	NO	NO	NO	NO	YES	NO	NO	NO	NO	NO	YES
*Cubic trend*	NO	NO	NO	NO	NO	YES	NO	NO	NO	NO	NO	YES
*RLMS year controls*	YES	YES	YES	YES	YES	YES	YES	YES	YES	YES	YES	YES
Num. obs.	127	127	127	332	332	434	138	138	138	395	395	613

***− 1 % sign., **− 5% sign., *− 10% sign. Coefficient std. errors are given in parentheses under the coefficient. Error terms are clustered at regional level

, 28

**Table 28:** Regression discontinuity estimates of the MC impact on the 2nd child, by poverty status (12-, 24-, and 36-month window around the cut-off birth date of 1 January 2007)

Outcome	Poor						Not poor					
	RDD12m	RDD12m	RDD12m	RDD24m	RDD24m	RDD36m	RDD12m	RDD12m	RDD12m	RDD24m	RDD24m	RDD36m
	(1)	(2)	(3)	(4)	(5)	(6)	(7)	(8)	(9)	(10)	(11)	(12)
In good/excellent health	0.0066	0.0212	0.0644	0.108	0.0353	$-$ 0.0037	$-$ 0.061	$-$ 0.065	0.025	0.042	0.064	$-$ 0.016
	(0.1779)	(0.1797)	(0.1828)	(0.1143)	(0.188)	(0.2124)	(0.114)	(0.117)	(0.109)	(0.087)	(0.113)	(0.122)
Health score (1$-$ highest, 5$-$ lowest)	$-$ 0.0612	$-$ 0.0687	$-$ 0.0926	$-$ 0.1757	$-$ 0.054	0.0406	0.069	0.079	$-$ 0.023	$-$ 0.068	$-$ 0.112	0.013
	(0.1822)	(0.1828)	(0.1832)	(0.1206)	(0.194)	(0.2214)	(0.128)	(0.131)	(0.121)	(0.099)	(0.134)	(0.144)
Num. of chronic conditions	$-$ 0.2178	$-$ 0.2158	$-$ 0.4303	0.0059	$-$ 0.2581	$-$ 0.4285	$-$ 0.073	$-$ 0.059	$-$ 0.147	$-$ 0.145	$-$ 0.372	$-$ 0.091
	(0.2768)	(0.2904)	(0.3644)	(0.1728)	(0.328)	(0.3406)	(0.173)	(0.174)	(0.172)	(0.125)	(0.188)**	(0.214)
Health problem in last 30d	0.0327	0.0477	0.0558	$-$ 0.1211	$-$ 0.0882	0.0671	0.131	0.103	0.068	$-$ 0.004	0.022	0.109
	(0.2106)	(0.2157)	(0.2295)	(0.1309)	(0.2234)	(0.2497)	(0.149)	(0.144)	(0.145)	(0.1)	(0.159)	(0.181)
Extracurricular study (in mins per week)	37.3355	33.3723	48.6479	$-$ 2.2727	14.9601	14.0649	11.181	5.069	13.094	26.527	26.569	15.199
	(31.1664)	(26.4059)	(40.4226)	(9.861)	(16.7294)	(17.0451)	(16.649)	(17.08)	(19.331)	(15.384)*	(19.936)	(20.605)
Extracurricular arts (in mins per week)	$-$ 7.5109	11.4326	9.1834	36.3554	41.953	70.7786	92.277	78.107	78.37	$-$ 12.421	100.453	144.127
	(96.9943)	(93.7509)	(112.6079)	(66.084)	(106.9688)	(101.3895)	(63.512)	(60.909)	(54.907)	(45.369)	(58.3)*	(70.617)**
Watching TV/on Internet (in mins per week)	52.6445	64.8348	38.8695	34.9119	86.9164	102.8151	$-$ 19.844	$-$ 20.689	$-$ 26.043	$-$ 8.787	$-$ 16.844	$-$ 38.531
	(44.7298)	(43.0051)	(48.0096)	(31.0222)	(49.18)*	(50.9862)**	(25.436)	(25.851)	(27.084)	(20.527)	(24.019)	(28.932)
Extracurricular sports (in mins per week)	337.6495	334.6388	321.659	$-$ 104.6229	138.5243	215.3093	26.412	40.121	68.092	$-$ 84.049	8.884	80.841
	(167.9499)**	(172.4352)*	(197.3872)	(135.8868)	(186.39)	(192.6782)	(150.452)	(153.409)	(143.845)	(96.712)	(153.017)	(170.905)
Used a personal computer	0.1629	0.2324	0.2497	0.0508	0.3766	0.1583	0.121	0.076	0.113	0.031	0.169	0.245
	(0.2661)	(0.2635)	(0.2641)	(0.1683)	(0.2689)	(0.2902)	(0.186)	(0.194)	(0.169)	(0.13)	(0.177)	(0.195)
Vacation with parent in 1yr	0.0845	0.1299	$-$ 0.1064	$-$ 0.1055	0.31	0.246	0.019	$-$ 0.024	0.012	$-$ 0.074	0.181	0.32
	(0.2127)	(0.2234)	(0.2424)	(0.1392)	(0.2481)	(0.277)	(0.145)	(0.145)	(0.129)	(0.098)	(0.141)	(0.151)**
Num. of excursions/exhibition visits in 1yr	$-$ 5.4124	$-$ 5.1314	$-$ 6.8211	$-$ 1.987	$-$ 4.0069	$-$ 6.0198	$-$ 2.502	$-$ 2.762	$-$ 0.888	0.239	$-$ 0.056	$-$ 0.684
	(2.8068)*	(2.9814)*	(3.0906)**	(1.6057)	(3.1785)	(3.5698)*	(2.31)	(2.362)	(2.074)	(1.487)	(2.194)	(2.546)
Sees friends $>2$ times per week	$-$ 0.0831	$-$ 0.1172	$-$ 0.0786	$-$ 0.0579	$-$ 0.2561	$-$ 0.3076	$-$ 0.33	$-$ 0.28	$-$ 0.258	$-$ 0.186	$-$ 0.368	$-$ 0.362
	(0.2094)	(0.2099)	(0.2181)	(0.1318)	(0.2223)	(0.2501)	(0.152)**	(0.154)*	(0.157)	(0.106)*	(0.165)**	(0.181)**
Received childcare in past 7 days	0.2747	0.2765	0.17	0.0806	0.2618	0.2914	$-$ 0.008	0.019	0.089	$-$ 0.072	$-$ 0.11	0.035
	(0.1657)*	(0.1674)	(0.1858)	(0.1113)	(0.1748)	(0.1934)	(0.146)	(0.147)	(0.146)	(0.101)	(0.149)	(0.168)
Received childcare from a relative in past 7 days	$-$ 0.1022	$-$ 0.0893	$-$ 0.0956	$-$ 0.0698	$-$ 0.0135	0.0546	0.045	0.053	0.054	$-$ 0.017	$-$ 0.092	0.05
	(0.1388)	(0.1407)	(0.1541)	(0.0945)	(0.1573)	(0.1669)	(0.119)	(0.119)	(0.128)	(0.081)	(0.127)	(0.141)
Mothers subjective life satisfaction (1$-$ lowest, 5$-$ highest)	$-$ 0.02	0.0258	$-$ 0.3591	0.121	0.4737	0.4595	0.445	0.424	0.499	0.173	0.638	0.737
	(0.4509)	(0.4495)	(0.4387)	(0.3038)	(0.4497)	(0.4947)	(0.272)	(0.272)	(0.28)*	(0.186)	(0.303)**	(0.34)**
*Age controls*	NO	YES	YES	YES	YES	YES	NO	YES	YES	YES	YES	YES
*Other controls*	NO	NO	YES	NO	YES	YES	NO	NO	YES	NO	NO	YES
*Linear trend*	YES	YES	YES	YES	YES	YES	YES	YES	YES	YES	YES	YES
*Quadratic trend*	NO	NO	NO	NO	NO	YES	NO	NO	NO	NO	NO	YES
*Cubic trend*	NO	NO	NO	NO	NO	YES	NO	NO	NO	NO	NO	YES
*RLMS year controls*	YES	YES	YES	YES	YES	YES	YES	YES	YES	YES	YES	YES
Num. obs.	138	138	138	285	285	433	191	191	191	445	445	614

***− 1 % sign., **− 5% sign., *− 10% sign. Coefficient std. errors are given in parentheses under the coefficient. Errors are clustered at regional level

, 29

**Table 29:** Regression discontinuity estimates of the MC impact on the 2nd child, by poverty status (12-, 24-, and 36-month window around the cut-off birth date of 1 January 2007), continued

outcome	Poor						Not poor					
	RDD12m	RDD12m	RDD12m	RDD24m	RDD24m	RDD36m	RDD12m	RDD12m	RDD12m	RDD24m	RDD24m	RDD36m
	(1)	(2)	(3)	(4)	(5)	(6)	(7)	(8)	(9)	(10)	(11)	(12)
Clothes (in Rub)	$-$ 1.7789	$-$ 1.7777	$-$ 1.0839	$-$ 2.2581	$-$ 0.838	$-$ 1.38	$-$ 2.088	$-$ 2.035	$-$ 0.865	0.15	$-$ 1.18	$-$ 1.956
	(1.3694)	(1.3674)	(1.3559)	(1.0034)**	(1.372)	(1.5881)	(1.193)*	(1.177)*	(0.992)	(0.957)	(1.224)	(1.301)
Essential food items (in Rub)	1.0572	0.9217	$-$ 0.0479	0.3049	5.245	4.6662	0.611	0.157	0.518	1.255	0.309	0.596
	(2.4443)	(2.4699)	(2.7762)	(1.7382)	(2.6082)**	(2.7757)*	(1.682)	(1.644)	(1.63)	(1.272)	(1.956)	(2.13)
Utilities (in Rub)	0.9633	1.5029	0.9532	0.5538	2.1643	0.9021	1.387	0.868	1.467	0.466	2.016	2.036
	(0.9091)	(0.9653)	(1.209)	(0.8023)	(0.8962)**	(0.955)	(1.581)	(1.398)	(1.285)	(0.916)	(1.524)	(1.766)
Essential services (in Rub)	1.5512	1.6052	0.2608	$-$ 0.2205	0.2479	0.5795	$-$ 0.139	$-$ 0.278	0.132	$-$ 0.095	0.281	0.26
	(1.9101)	(1.7711)	(1.3386)	(1.4211)	(1.8395)	(2.0228)	(0.889)	(0.838)	(0.731)	(0.503)	(0.796)	(0.901)
All basic expenditure (in Rub)	1.7928	2.252	0.0822	$-$ 1.6199	6.8192	4.7678	$-$ 0.229	$-$ 1.288	1.252	1.775	1.426	0.935
	(3.9745)	(3.8331)	(4.1489)	(2.8806)	(3.7192)*	(4.0971)	(3.699)	(3.396)	(2.95)	(2.453)	(3.367)	(3.767)
All non$-$ essential expenditure (in Rub)	$-$ 0.0212	0.3564	$-$ 0.7065	3.8163	6.5401	5.3965	$-$ 0.193	0.218	2.834	0.768	4.877	5.073
	(1.91)	(1.8057)	(2.1042)	(3.1352)	(5.5393)	(5.1307)	(2.384)	(2.368)	(2.673)	(2.925)	(2.862)*	(3.398)
Purchase of durable goods (in Rub)	$-$ 0.2397	$-$ 0.2611	$-$ 0.1934	$-$ 0.2142	0.0884	$-$ 0.1678	0.134	0.119	0.09	0.028	0.078	0.195
	(0.2119)	(0.2139)	(0.2279)	(0.1346)	(0.2276)	(0.2508)	(0.155)	(0.157)	(0.151)	(0.108)	(0.159)	(0.182)
Sh. Essential food items	0.0117	$-$ 0.0349	0.0195	0.0699	0.1306	0.2212	0.041	0.055	0.024	0.008	$-$ 0.052	0.005
	(0.1247)	(0.123)	(0.1331)	(0.0957)	(0.1295)	(0.1401)	(0.051)	(0.053)	(0.051)	(0.036)	(0.062)	(0.072)
Sh. Clothes	$-$ 0.1574	$-$ 0.1803	$-$ 0.1239	$-$ 0.0873	$-$ 0.1301	$-$ 0.0719	$-$ 0.001	0.012	0.001	0.02	$-$ 0.027	$-$ 0.009
	(0.0874)*	(0.0975)*	(0.092)	(0.0586)	(0.0953)	(0.1177)	(0.037)	(0.038)	(0.036)	(0.026)	(0.039)	(0.043)
Sh. Non$-$ essential expenditure	$-$ 0.0014	0.0017	$-$ 0.0037	0.1248	0.1439	0.1327	0.016	0.041	0.083	$-$ 0.06	0.111	0.04
	(0.0754)	(0.071)	(0.0876)	(0.0634)**	(0.1178)	(0.111)	(0.075)	(0.078)	(0.091)	(0.091)	(0.096)	(0.165)
Sh. Savings	$-$ 0.0884	$-$ 0.0986	$-$ 0.099	$-$ 0.0769	$-$ 0.0898	$-$ 0.105	0.07	0.077	0.099	0.094	0.075	0.071
	(0.0542)	(0.0555)*	(0.0559)*	(0.043)*	(0.0574)	(0.067)	(0.083)	(0.091)	(0.106)	(0.055)*	(0.097)	(0.096)
Sh. Loan payments	0.0641	0.0602	0.1486	0.0741	0.1117	0.0911	0.08	0.09	0.097	0.04	0.092	0.139
	(0.0555)	(0.0552)	(0.0748)**	(0.0507)	(0.0658)*	(0.0788)	(0.052)	(0.052)*	(0.052)*	(0.037)	(0.06)	(0.075)*
Sh. Utilities	0.0388	0.0488	0.0326	0.0541	0.0655	0.035	0.019	0.012	0.006	0.005	0.008	0.041
	(0.0541)	(0.0607)	(0.0567)	(0.0362)	(0.0568)	(0.0597)	(0.023)	(0.022)	(0.022)	(0.015)	(0.025)	(0.04)
Sh. Essential services	$-$ 0.0166	$-$ 0.018	$-$ 0.0335	0.0173	$-$ 0.0436	$-$ 0.0075	0.005	0.004	0.001	$-$ 0.008	0.006	0.006
	(0.062)	(0.0619)	(0.0551)	(0.0369)	(0.0684)	(0.0794)	(0.017)	(0.017)	(0.018)	(0.011)	(0.02)	(0.023)
Sh. Basic expenditure	$-$ 0.1234	$-$ 0.1844	$-$ 0.1053	0.054	0.0223	0.1769	0.063	0.083	0.032	0.025	$-$ 0.065	0.043
	(0.2077)	(0.2209)	(0.2266)	(0.1482)	(0.2201)	(0.2549)	(0.081)	(0.083)	(0.079)	(0.058)	(0.092)	(0.116)
*Other controls*	NO	NO	YES	NO	NO	YES	NO	NO	YES	NO	NO	YES
*Linear trend*	YES	YES	YES	YES	YES	YES	YES	YES	YES	YES	YES	YES
*Quadratic trend*	NO	YES	YES	NO	YES	YES	NO	YES	YES	NO	YES	YES
*Cubic trend*	NO	YES	YES	NO	YES	YES	NO	YES	YES	NO	YES	YES
*RLMS year controls*	NO	YES	YES	NO	YES	YES	NO	YES	YES	NO	YES	YES
Num. obs.	138	138	138	285	285	433	191	191	191	445	445	614

***− 1 % sign., **− 5% sign., *− 10% sign. Coefficient std. errors are given in parentheses under the coefficient. Error terms are clustered at regional level

and 30.

**Table 30:** Regression discontinuity estimates of the MC impact on the 2nd child, by poverty status (12-, 24-, and 36-month window around the cut-off birth date of 1 January 2007), continued

Outcome	Poor						Not poor					
	RDD12m	RDD12m	RDD12m	RDD24m	RDD24m	RDD36m	RDD12m	RDD12m	RDD12m	RDD24m	RDD24m	RDD36m
	(1)	(2)	(3)	(4)	(5)	(6)	(7)	(8)	(9)	(10)	(11)	(12)
Vegetables/legumes (in kg)	$-$ 2.0073	$-$ 2.2061	$-$ 3.5106	0.2373	$-$ 1.9054	$-$ 3.9988	$-$ 0.856	$-$ 0.967	$-$ 0.673	$-$ 0.47	$-$ 0.832	$-$ 1.027
	(3.5704)	(3.5881)	(3.4709)	(1.8537)	(3.5841)	(4.2621)	(1.016)	(1.012)	(1.02)	(0.76)	(1.351)	(1.348)
Fruit (fresh and canned) (in kg)	$-$ 1.735	$-$ 1.7488	$-$ 2.5126	$-$ 0.1355	$-$ 0.7516	$-$ 1.4453	0.029	0.003	0.372	0.327	0.59	0.015
	(1.661)	(1.6759)	(1.6594)	(0.9859)	(1.7191)	(2.0692)	(1.303)	(1.34)	(1.431)	(0.754)	(1.286)	(1.444)
Meat and poultry (in kg)	0.8895	1.0638	0.1003	0.9501	2.4623	2.1039	$-$ 1.08	$-$ 1.24	$-$ 1.217	$-$ 0.665	$-$ 1.005	$-$ 1.085
	(1.2201)	(1.2756)	(1.6803)	(0.9134)	(1.4162)*	(1.4483)	(0.893)	(0.857)	(0.856)	(0.631)	(0.985)	(1.078)
Dairy (in kg)	2.9377	3.0913	0.9523	0.2546	3.1233	1.8824	1.142	0.911	0.725	$-$ 1.117	0.148	0.085
	(1.6553)*	(1.6857)*	(1.6579)	(1.389)	(1.8103)*	(1.9258)	(1.484)	(1.478)	(1.346)	(1.017)	(1.525)	(1.676)
Vodka and liquors (in litres)	0.13	0.1527	0.1729	0.1347	0.2707	0.164	$-$ 0.12	$-$ 0.154	$-$ 0.147	$-$ 0.229	$-$ 0.116	$-$ 0.106
	(0.089)	(0.0887)*	(0.086)**	(0.0893)	(0.151)*	(0.1882)	(0.188)	(0.183)	(0.181)	(0.126)*	(0.209)	(0.236)
Refined sugar (in kg)	$-$ 0.3796	$-$ 0.4175	$-$ 0.5214	$-$ 0.2943	0.4334	0.6467	0.691	0.864	0.631	0.042	$-$ 0.199	0.24
	(0.9256)	(0.9322)	(0.8303)	(0.5125)	(0.8843)	(1.0275)	(0.676)	(0.67)	(0.632)	(0.514)	(0.68)	(0.732)
Candy and high-sugar treats (in kg)	$-$ 0.1731	$-$ 0.2136	0.2454	$-$ 0.4438	0.3024	0.2368	0.354	0.313	0.324	0.245	0.456	0.46
	(0.8606)	(0.8553)	(0.8603)	(0.5233)	(0.9589)	(1.0866)	(0.369)	(0.376)	(0.372)	(0.27)	(0.501)	(0.553)
Starches (in kg)	$-$ 3.3089	$-$ 3.8639	$-$ 3.9807	$-$ 0.2426	1.0641	2.0358	2.169	2.473	0.537	2.292	$-$ 2.799	$-$ 3.309
	(3.1586)	(3.1857)	(3.0127)	(2.0674)	(3.2915)	(3.6141)	(2.686)	(2.707)	(2.316)	(1.843)	(2.74)	(2.976)
Household savings in last 30 days (in 2011 prices)	$-$ 2.81	$-$ 3.8511	$-$ 4.1967	$-$ 5.3926	$-$ 3.3396	$-$ 3.1641	0.792	0.602	1.509	3.061	2.184	1.089
	(1.985)	(2.3407)	(2.8175)	(4.0401)	(2.3863)	(2.3108)	(1.659)	(1.899)	(2.25)	(1.541)**	(2.45)	(2.443)
Savings will last several months	$-$ 0.1034	$-$ 0.1129	$-$ 0.0734	$-$ 0.1299	$-$ 0.1426	$-$ 0.1806	$-$ 0.09	$-$ 0.078	$-$ 0.043	0.128	0.006	0.033
	(0.1694)	(0.1752)	(0.164)	(0.1001)	(0.1755)	(0.1979)	(0.138)	(0.132)	(0.124)	(0.096)	(0.134)	(0.153)
Loan payments in last 30 days (in 2011 prices)	0.7173	1.5585	3.4575	2.2032	1.8664	0.7789	5.468	5.288	5.892	3.675	5.897	7.255
	(1.9137)	(2.1187)	(2.5132)	(1.6858)	(2.3304)	(2.5688)	(3.267)*	(3.129)*	(2.97)**	(2.037)*	(3.067)*	(3.596)**
Consumer loan in last 12 months	$-$ 0.1018	$-$ 0.0855	0.0044	$-$ 0.1073	$-$ 0.0246	$-$ 0.0816	0.084	0.089	0.082	0.145	0.07	0.153
	(0.1721)	(0.1748)	(0.1943)	(0.119)	(0.201)	(0.2189)	(0.11)	(0.109)	(0.107)	(0.083)*	(0.127)	(0.138)
Borrowed from a private individual in last 12 months	0.1942	0.1898	0.1537	0.0963	$-$ 0.0056	0.0618	0.184	0.193	0.207	0.138	0.167	0.104
	(0.1)*	(0.1018)*	(0.11)	(0.0812)	(0.1148)	(0.1205)	(0.069)***	(0.07)***	(0.074)***	(0.051)***	(0.076)**	(0.082)
Loan to a private individual in last 12 months	$-$ 0.1824	$-$ 0.1769	$-$ 0.1772	$-$ 0.0648	$-$ 0.13	$-$ 0.1353	0.005	0.004	0.003	0.015	$-$ 0.06	$-$ 0.056
	(0.1444)	(0.1433)	(0.1462)	(0.0774)	(0.153)	(0.1822)	(0.103)	(0.101)	(0.104)	(0.069)	(0.113)	(0.128)
Household owns home	0.0067	0.0117	$-$ 0.0596	0.0699	0.207	0.1064	$-$ 0.116	$-$ 0.111	$-$ 0.13	0.037	0.003	$-$ 0.062
	(0.1703)	(0.174)	(0.1682)	(0.1094)	(0.1679)	(0.1895)	(0.101)	(0.099)	(0.093)	(0.065)	(0.109)	(0.126)
Hot water supply	0.1328	0.1571	$-$ 0.0939	$-$ 0.0572	0.0771	$-$ 0.0132	0.024	$-$ 0.026	$-$ 0.001	0.055	0.072	0.144
	(0.2189)	(0.226)	(0.219)	(0.1412)	(0.2207)	(0.2385)	(0.159)	(0.153)	(0.121)	(0.103)	(0.135)	(0.153)
Central sewerage	0.4545	0.5289	0.1703	0.0192	0.4875	0.3875	0.032	$-$ 0.039	0.06	$-$ 0.061	0.176	0.228
	(0.2029)**	(0.1936)***	(0.1065)	(0.1298)	(0.1474)***	(0.1487)***	(0.151)	(0.146)	(0.065)	(0.102)	(0.1)*	(0.108)**
Central heating	0.4184	0.4848	0.1328	0.0463	0.2561	0.1846	0.009	$-$ 0.046	0.057	0.051	0.091	0.167
	(0.2061)**	(0.2005)**	(0.1612)	(0.1311)	(0.149)*	(0.1534)	(0.151)	(0.149)	(0.079)	(0.103)	(0.099)	(0.107)
*Age controls*	NO	YES	YES	YES	YES	YES	NO	YES	YES	YES	YES	YES
*Other controls*	NO	NO	YES	NO	YES	YES	NO	NO	YES	NO	NO	YES
*Linear trend*	YES	YES	YES	YES	YES	YES	YES	YES	YES	YES	YES	YES
*Quadratic trend*	NO	NO	NO	NO	NO	YES	NO	NO	NO	NO	NO	YES
*Cubic trend*	NO	NO	NO	NO	NO	YES	NO	NO	NO	NO	NO	YES
*RLMS year controls*	YES	YES	YES	YES	YES	YES	YES	YES	YES	YES	YES	YES
Num. obs.	138	138	138	285	285	433	191	191	191	445	445	614

***− 1 % sign., **− 5% sign., *− 10% sign. Coefficient std. errors are given in parentheses under the coefficient. Error terms are clustered at regional level

See Figs. 1, 2 and 3.

## Appendix 4: Models for Covariates

See Tables 31

**Table 31:** Lowess estimates of sample differences at the MC cut-off between mothers interviewed in RLMS 2010–2017 who gave the 2nd childbirth before and after January 2007 (12, 24 and 36 months’ window around 1 January 2007)

Variable	RDD 12m 2d kernel	RDD 12m 1d kernel	RDD 24m 2d kernel	RDD 24m 1d kernel	RDD 36m 2d kernel	RDD 36m 1d kernel
	(1)	(2)	(3)	(4)	(5)	(6)
*Mother’s characteristics*						
Age	0.368	0.723	$-$ 0.658	0.994	$-$ 0.84	0.314
Single parent	0.072	0.015	0.001	0.081	$-$ 0.009	0.036
Higher education	0.088	$-$ 0.102	$-$ 0.019	$-$ 0.029	$-$ 0.008	$-$ 0.029
In good/excellent health	$-$ 0.305	$-$ 0.506**	$-$ 0.038	$-$ 0.13	0.04	$-$ 0.075
Alcohol consumption $>1$ times per week	$-$ 0.003	0.003	$-$ 0.028	$-$ 0.051	$-$ 0.027	$-$ 0.043
*Household characteristics*						
Income per household member	0.106	0.093	$-$ 2.423	$-$ 2.265	$-$ 3.157*	$-$ 2.398
Self-identified poverty	0.283	0.212	0.18	0.245**	0.223**	0.186*
Num. of observations	328	328	727	727	1047	1047

***− 1 % sign., **− 5% sign., *− 10% sign

and 32.

**Table 32:** Regression discontinuity estimates of sample differences at the MC cut-off between mothers interviewed in RLMS 2010–2017 who gave the 2nd childbirth before and after January 2007 (12, 24 and 36 months’ window at 1 January 2007 cut-off birth date)

Variable	RDD 12m	RDD 12m	RDD 24m	RDD 24m	RDD 36m	RDD 36m
	(1)	(2)	(3)	(4)	(5)	(6)
*Mother’s characteristics*						
Age	1.886	1.51	$-$ 0.184	1.261	0.541	2.247
	(1.18)	(1.24)	(0.825)	(1.289)	(1.017)	(1.433)
Single parent	0.062	0.058	0	0.03	0.021	0.049
	(0.098)	(0.098)	(0.066)	(0.102)	(0.081)	(0.114)
Higher education	0.038	0.012	0.028	$-$ 0.029	0.006	0.067
	(0.099)	(0.102)	(0.073)	(0.109)	(0.089)	(0.12)
In good/excellent health	0.019	$-$ 0.018	0.073	0.004	0.148	$-$ 0.129
	(0.12)	(0.125)	(0.083)	(0.13)	(0.103)	(0.146)
Alcohol consumption $>1$ times per week	$-$ 0.053	$-$ 0.033	$-$ 0.008	$-$ 0.037	$-$ 0.036	$-$ 0.021
	(0.037)	(0.032)	(0.028)	(0.036)	(0.03)	(0.036)
*Household characteristics*						
Income per household member	$-$ 0.684	$-$ 1.079	$-$ 2.718	$-$ 1.791	$-$ 3.173	0.58
	(2.21)	(2.19)	(1.635)*	(1.903)	(1.769)*	(1.939)
Self-identified poverty	0.17	0.157	0.201	$-$ 0.037	0.172	$-$ 0.03
	(0.118)	(0.123)	(0.082)**	(0.127)	(0.103)*	(0.144)
*Child age controls*	NO	YES	YES	YES	YES	YES
*Linear trend*	YES	YES	YES	YES	YES	YES
*Quadratic trend*	NO	NO	NO	YES	YES	YES
*Cubic trend*	NO	NO	NO	NO	NO	YES
*RLMS year controls*	NO	YES	YES	YES	YES	YES
Num. obs.	328	328	727	727	1047	1047

***− 1 % sign., **− 5% sign., *− 10% sign. Coefficient std. errors are given in parentheses under the coefficient. Error terms are clustered at regional level

See Figs. 4 and 5.

## Appendix 5: Models for Covariates, RDD Effects in August 2007

See Table 33.

**Table 33:** Regression discontinuity estimates of sample differences between mothers who gave a 2nd childbirth before and after August 2007 (12- and 24-month window at 1 August 2007)

Variable	RDD 12m	RDD 12m	RDD 24m	RDD 24m	LOWESS 12m	LOWESS 24m
	(1)	(2)	(3)	(4)	(5)	(6)
*Mother’s characteristics*						
Age	$-$ 0.398	$-$ 0.548	$-$ 0.093	$-$ 0.304	1.389	$-$ 0.311
	(1.104)	(1.118)	(0.823)	(1.149)		
Single parent	$-$ 0.077	$-$ 0.091	$-$ 0.033	$-$ 0.073	$-$ 0.144	$-$ 0.05
	(0.06)	(0.059)	(0.051)	(0.062)		
Higher education	$-$ 0.207	$-$ 0.183	$-$ 0.198	$-$ 0.225	$-$ 0.269*	$-$ 0.216*
	(0.097)**	(0.097)*	(0.071)***	(0.102)**		
In good/excellent health	$-$ 0.006	$-$ 0.006	0.083	0	0.032	0.029
	(0.106)	(0.107)	(0.079)	(0.114)		
Alcohol consumption $>1$ times per week	0.108	0.106	0.054	0.112	0.121**	0.077*
	(0.046)**	(0.049)**	(0.033)*	(0.052)**		
*Household characteristics*						
Income per household member	2.379	2.647	0.548	2.717	2.409	1.083
	(1.713)	(1.739)	(1.175)	(1.83)		
Self$-$ identified poverty	$-$ 0.002	$-$ 0.024	$-$ 0.111	0.039	$-$ 0.103	$-$ 0.028
	(0.106)	(0.107)	(0.078)	(0.114)		
*Child age controls*	NO	YES	YES	YES		
*Linear trend*	YES	YES	YES	YES		
*Quadratic trend*	NO	NO	NO	YES		
*Cubic trend*	NO	NO	NO	NO		
*RLMS year controls*	NO	YES	YES	YES		
Num. obs.	373	373	730	730	373	730

***− 1 % sign., **− 5% sign., *− 10% sign. Coefficient std. errors are given in parentheses under the coefficient. Error terms are clustered at regional level in parametric RDDs

## Footnotes

1 Urban is defined as living in a city or in a regional centre, in accordance with the RLMS statistical classification
